# Novel paradigm enables accurate monthly gestational screening to prevent congenital toxoplasmosis and more

**DOI:** 10.1371/journal.pntd.0011335

**Published:** 2024-05-28

**Authors:** Ying Zhou, Karen Leahy, Andrew Grose, Joseph Lykins, Maryam Siddiqui, Nicole Leong, Perpetua Goodall, Shawn Withers, Kevin Ashi, Stephen Schrantz, Vera Tesic, Ana Precy Abeleda, Kathleen Beavis, Fatima Clouser, Mahmoud Ismail, Monica Christmas, Raphael Piarroux, Denis Limonne, Emmanuelle Chapey, Sylvie Abraham, Isabelle Baird, Juliette Thibodeau, Kenneth M. Boyer, Elizabeth Torres, Shannon Conrey, Kanix Wang, Mary Allen Staat, Nancy Back, Coralie L’Ollivier, Caroline Mahinc, Pierre Flori, Jorge Gomez-Marin, Francois Peyron, Sandrine Houzé, Martine Wallon, Rima McLeod

**Affiliations:** 1 Departments of Ophthalmology and Visual Science, The University of Chicago, Chicago, Illinois, United States of America; 2 Department of Obstetrics and Gynecology, The University of Chicago, Chicago, Illinois, United States of America; 3 Pritzker School of Medicine, Division of Infectious Diseases, The University of Chicago, Chicago, Illinois, United States of America; 4 Chicago Medicine, The University of Chicago, Chicago, Illinois, United States of America; 5 Department of Pediatrics, Division of Infectious Diseases, The University of Chicago, Chicago, Illinois, United States of America; 6 Department of Medicine, The University of Chicago, Chicago, Illinois, United States of America; 7 Department of Pathology, The University of Chicago, Chicago, Illinois, United States of America; 8 LDBIO Diagnostics, Lyon, France; 9 Institut des agents infectieux, Hôpital de la Croix-Rousse, Lyon, France; 10 Laboratory of Parasitologie, Bichat-Claude Bernard Hôpital, Paris, France; 11 The College, The University of Chicago, Chicago, Illinois, United States of America; 12 Global Health Center, The University of Chicago, Chicago, Illinois, United States of America; 13 Department of Pediatrics, Division of Infectious Diseases, Rush Presbyterian Hospital and Medical Center, Chicago, Illinois, United States of America; 14 Group of Molecular Parasitology (GEPAMOL), Center of Biomedical Research, Faculty of Health Sciences, University of Quindio, Armenia (Quindio), Colombia; 15 University of Cincinnati and Cincinnati Children’s Hospital Medical Center, University of Cincinnati, Cincinnati, Ohio, United States of America; 16 Carl H. Lindner College of Business, The University of Cincinnati, Cincinnati, Ohio, United States of America; 17 Centre National de Référence Toxoplasmose—Pôle Sérologie, Hôpitaux Universitaires de Strasbourg, Strasbourg, France; 18 IHU-Méditerranée Infection, Assistance Publique Hôpitaux de Marseille (AP-HM), Marseille, France; Aix Marseille University, IRD, AP-HM, SSA, VITROME, IHU Méditerranée, Marseille, France; 19 Parasitology and Mycology Laboratory, Pôle de Biologie-Pathologie, University Hospital of Saint Etienne, Saint Etienne, France; Universidade Federal de Minas Gerais, BRAZIL

## Abstract

**Background:**

Congenital toxoplasmosis is a treatable, preventable disease, but untreated causes death, prematurity, loss of sight, cognition and motor function, and substantial costs worldwide.

**Objectives:**

We asked whether high performance of an Immunochromatographic-test (ICT) could enable accurate, rapid diagnosis/treatment, establishing new, improved care-paradigms at point-of-care and clinical laboratory.

**Methods:**

Data were obtained in 12 studies/analyses addressing: 1-feasibility/efficacy; 2-false-positives; 3-acceptability; 4-pink/black-line/all studies; 5-time/cost; 6-Quick-Information/Limit-of-detection; 7, 8-acute;-chronic; 9-epidemiology; 10-ADBio; 11,12-Commentary/Cases/Chronology.

**Findings:**

ICT was compared with gold-standard or predicate-tests. Overall, ICT performance for 1093 blood/4967 sera was 99.2%/97.5% sensitive and 99.0%/99.7% specific. However, in clinical trial, FDA-cleared-predicate tests initially caused practical, costly problems due to false-positive-IgM results. For 58 persons, 3/43 seronegative and 2/15 chronically infected persons had false positive IgM predicate tests. This caused substantial anxiety, concerns, and required costly, delayed confirmation in reference centers. Absence of false positive ICT results contributes to solutions: Lyon and Paris France and USA Reference laboratories frequently receive sera with erroneously positive local laboratory IgM results impeding patient care. Therefore, thirty-two such sera referred to Lyon’s Reference laboratory were ICT-tested. We collated these with other earlier/ongoing results: 132 of 137 USA or French persons had false-positive local laboratory IgM results identified correctly as negative by ICT. Five false positive ICT results in Tunisia and Marseille, France, emphasize need to confirm positive ICT results with Sabin-Feldman-Dye-test or western blot. Separate studies demonstrated high performance in detecting acute infections, meeting FDA, CLIA, WHO REASSURED, CEMark criteria and patient and physician satisfaction with monthly-gestational-ICT-screening.

**Conclusions/significance:**

This novel paradigm using ICT identifies likely false positives or raises suspicion that a result is truly positive, rapidly needing prompt follow up and treatment. Thus, ICT enables well-accepted gestational screening programs that facilitate rapid treatment saving lives, sight, cognition and motor function. This reduces anxiety, delays, work, and cost at point-of-care and clinical laboratories.

**Trial registration:**

NCT04474132, https://clinicaltrials.gov/study/NCT04474132

ClinicalTrials.gov

## Author summary

Toxoplasmosis is a major health burden for developed and developing countries, causing damage to eyes and brain, loss of life and substantial societal costs. Prompt diagnosis in gestational screening programs enables treatment, thereby relieving suffering, and leading to > 14-fold cost savings for care. Herein, we demonstrate that using an ICT that meets WHO REASSURED-criteria identifying persons with/without antibody to *Toxoplasma gondii* in sera and whole blood with high sensitivity and specificity, is feasible to use in USA clinical practice. We find this new approach can help to obviate the problem of detection of false positive anti-*T*. *gondii* IgM results for those without IgG antibodies to *T*. *gondii* when this occurs in present, standard of care, predicate USA FDA cleared available assays. Thus, this accurate test facilitates gestational screening programs and a global initiative to diagnose and thereby prevent and treat *T*. *gondii* infection. This minimizes likelihood of false positives (IgG and/or IgM) while maintaining maximum sensitivity. When isolated IgM antibodies are detected, it is necessary to confirm and when indicated continue follow up testing in ~2 weeks to establish seroconversion. Presence of a positive ICT makes it likely that IgM is truly positive and a negative ICT makes it likely that IgM will be a false positive without infection. These results create a new, enthusiastically-accepted, precise paradigm for rapid diagnosis and validation of results with a second-line test. This helps eliminate alarm and anxiety about false-positive results, while expediting needed treatment for true positive results and providing back up distinguishing false positive tests.

## Introduction

### Background, what is known

*Toxoplasma gondii*, infects approximately half of all persons with 16 million people infected congenitally. Congenital toxoplasmosis (CT) causes loss of life, sight, cognitive and motor function [1–5]. In 2013 the World Health Organization (WHO) estimated there are up to 190,100 new cases of CT and 1.20 million disability-adjusted life years each year globally [4–7]. Disease burden is particularly high in Latin America and certain populations in the US and elsewhere with high exposure. Almost all untreated congenitally infected persons develop manifestations [1–17]. In addition to considerable progress toward definitive cure and prevention of *Toxoplasma* infection with novel potential medicines and vaccines [6,7], a critical part of eliminating the disease and reducing suffering and disease burden of CT requires prompt recognition of seroconversion and expeditious, early treatment of the acutely infected pregnant women with available, effective medicines [3,6–9]. Screening monthly, beginning before or near conception to one month post-partum for development of antibody to the parasite in previously seronegative women can enable treatment to prevent trans-placental transmission of newly acquired maternal *Toxoplasma* infection or treat the fetus to prevent sequelae [3,7–17]. France, Austria, Slovenia, Colombia, Panama, Brazil, Argentina and Morocco have or are developing screening programs [8,10,11] but the United States does/has not [3,6,7,8,12–14]. Actual and artificially inflated costs to make profit are potential barriers [1,13–45], even though cost benefit analyses all have found substantial cost savings and benefits with routine testing [13–18]. Introduction of prenatal screening tests that fulfill the WHO REASSURED criteria (Real-time connectivity, Ease of use, Affordable, Sensitive, Specific, User-friendly, Rapid, Robust, Equipment-free, Deliverable) can improve benefit [13–18]. False positive results using currently available commercial test kits for anti-*Toxoplasma* IgM compound problems [19–22,38]. In 1998, the United States Food and Drug Administration (FDA) mandated that a positive result for acute infection (IgM) with a non-reference laboratory (NRL) test should be confirmed at the Palo Alto Medical Foundation /Remington Specialty *Toxoplasma* Serology Laboratory (PAMF-TSL) [22]. However, this can and often does lead to associated delays, and causes concern for patients and their physicians. Substantial costs (more than $800 per panel of tests, which may require repeat testing, in the USA with additional problems for this exceptional testing from insurance denials and capitation of obstetrical health care) have been arguments against screening programs [14]. Therefore, NRL tests with high specificity and low cost are needed. A recently developed lateral immunochromatography test with antigen on beads that captures IgG and /or IgM antibodies, *Toxoplasma* ICT IgG-IgM test (LDBIO Diagnostic, Lyon, France, hereafter called ICT) is a promising candidate NRL test that satisfies REASSURED criteria [23–5]. Nonetheless, positive results influencing clinical care still require clinical reference laboratory confirmation.

### Gaps in knowledge

Gaps include whether while meeting World Health Organization REASSURED criteria the ICT could achieve the specific regulatory requirements for European CE Mark in Europe and USA dual 510K FDA clearance and Clinical Laboratory Improvements Amendments (CLIA) waiver. For the dual FDA clearance and CLIA waiver, this process required at least 240 matrix tests of whole blood and serum with half from seropositive persons. It was essential that 1/3 of the data be obtained from the USA from at least 3 sites and 3 testers with at least 5 seropositive persons for each tester. Limit of detection and Quick Information studies with 9 testers to determine that this test could be easily utilized with written instructions by untrained professionals (not lay users) were other gaps. Other unanswered questions were determining safety and efficacy in practice at the point of care and in the laboratory, patient and provider satisfaction, applicability in Middle-Low income countries, utility in epidemiologic studies, and whether and how this could be integrated into clinical practice easily in the flow of usual clinical care. Whether the ICT could function comparably to current commercial and reference laboratory tests for those with low, mid-range and high antibody levels, or whether other illnesses interfered with the test also were critical questions to consider.

### Hypotheses

Hypotheses were that **s**tudying the ICT would provide data that meet all the specific requirements for CE mark and dual 510 K FDA clearance and CLIA waiver while demonstrating that the ICT would have: 1) Real-time connectivity, **E**ase of specimen collection, and be Affordable, **S**ensitive, **S**pecific, User friendly, **R**apid, **R**obust, **E**quipment free, and **D**eliverable to end users. In meeting these REASSURED criteria, results would enable screening for acquisition of *Toxoplasma gondii* infection during gestation in a manner faster and more accurately than other commercially available tests; This testing would be: 2) less costly than other commercial tests; 3) preferred by patients and clinicians to other test protocols; and 4) useful for diagnosing infection in North and Latin America, Europe, and North Africa.

### Objectives

In 12 sequential studies and analyses we aimed to learn how well the ICT met WHO REASSURED criteria and guidelines for achieving FDA clearance and CLIA waiver in the USA and CE Mark in Europe. This work also was designed to determine whether it is feasible to use the ICT, maintaining its high performance, with users in multiple clinical circumstances and settings. We also intended to determine acceptability of the use of the ICT to patients, families, and front-line health care personnel. In so doing we intended to learn how the ICT also might be used optimally in the clinic and in the laboratory for rapid back up testing to address whether false positive results in standard commercial tests might have occurred. If these criteria were met, this test could help to enable serologic screening to detect, diagnose and thereby promptly treat to prevent this infection and its adverse sequelae providing an improved paradigm for care to prevent or ameliorate the manifestations of this infection. When we encountered difficulties in the first 6 tests with false positive IgM results with the commercial, predicate comparator compared with gold standard tests in our feasibility clinical trial, but not the ICT, we were faced with the unanticipated constraint of cost of positive confirmation of a number of tests at PAMF-TSL, and recognition that this type of cost from the frequent false positive IgM results could de-rail screening programs in the United States. Given the true negative ICT results when compared with gold standard tests in our setting, we queried whether the ICT could be part of a paradigm to rule-out false positive IgM results with NRL test, both at the point-of-care and in the hospital clinical laboratory. Further, we tested samples with the ICT that were suspected false positives from local laboratories that had been referred to reference laboratories.

### Implications of unexpected findings leading to next steps

Our unanticipated findings of false positive NRL commercial predicate tests led us to identify a solution for our clinical problem: We placed these data in the context of practical clinical problems we encountered and collated our results with ongoing and reported similar studies to define whether this could be an approach helpful in addressing false positive predicate NRL test results. Solving this problem emphasized how the ICT can be used in screening programs to benefit pregnant women and their families, creating a new model to approach the problem of need for accurate screening and of false positive NRL tests. This highly accurate test may help enable screening for acquisition of *T*. *gondii* in gestation [23–39], as well as being useful in other settings, and thereby contribute to saving sight, cognition, motor function and lives and improve quality of life [1–10,12–15,17,24,35].

## Methods

### Ethics

The ongoing UCMC study, under the name “Prevention of CT: Feasibility of prenatal screening using point-of-care tests,” is conducted with ethical standards for human experimentation established in the Declaration of Helsinki. Research received approval from the University of Chicago Institutional Review Board (University of Chicago IRB Protocols 20–0442, 19–0505, 21–1446, 8793, 8794, 8795, 8796, 8797, 8798, 16708A and met the standards of the Health Insurance Portability and Accountability Act. Results were or will be reviewed by/discussed with the Data Safety Monitoring Board. Informed consents were obtained from subjects in accordance with the University of Chicago Institutional Review Board and the National Institutes of Health guidelines. No subjects are under age 18 years in the University of Chicago Clinical Trial. All participants provided informed, written consent prior to their study participation. All studies were performed in accordance with the Declaration of Helsinki.

Samples from the Lyon Reference laboratory of the University hospital were anonymized in this analysis. Testing in Colombia was performed in accordance with local Ethics Committees approvals and guidelines.

Stored sera from Cincinnati was approved by the IRB for future testing for a wide range of pathogens.

### Overall goals

These methods were structured to meet our initial specific objectives to determine the performance of the ICT. When we found high performance in initial studies objectives were then to carry out studies to assess whether the test met WHO REASSURED criteria for a point of care test, to meet all CE Mark, FDA and CLIA testing requirements, and to determine feasibility of implementation of use of ICT in multiple “real-life” clinical settings.

### Design

We performed a series of studies with a lateral immunochromatography test (ICT) we previously found met WHO REASSURED criteria with serum and whole blood from a finger prick. This new work performed herein included prospective real time studies of feasibility and acceptability in the USA, France, and Colombia. In so doing, we discovered paradigm shifting approaches and utility, proving our original hypotheses and extending beyond these hypotheses. These studies used methods that follow. **Box 1** lists and provides a “roadmap” to and milestones for 12 studies/analyses in this decades-long work. An overview of methods follows:

### Study 1: Feasibility clinical trial study

#### Overall

**Fig 1:**
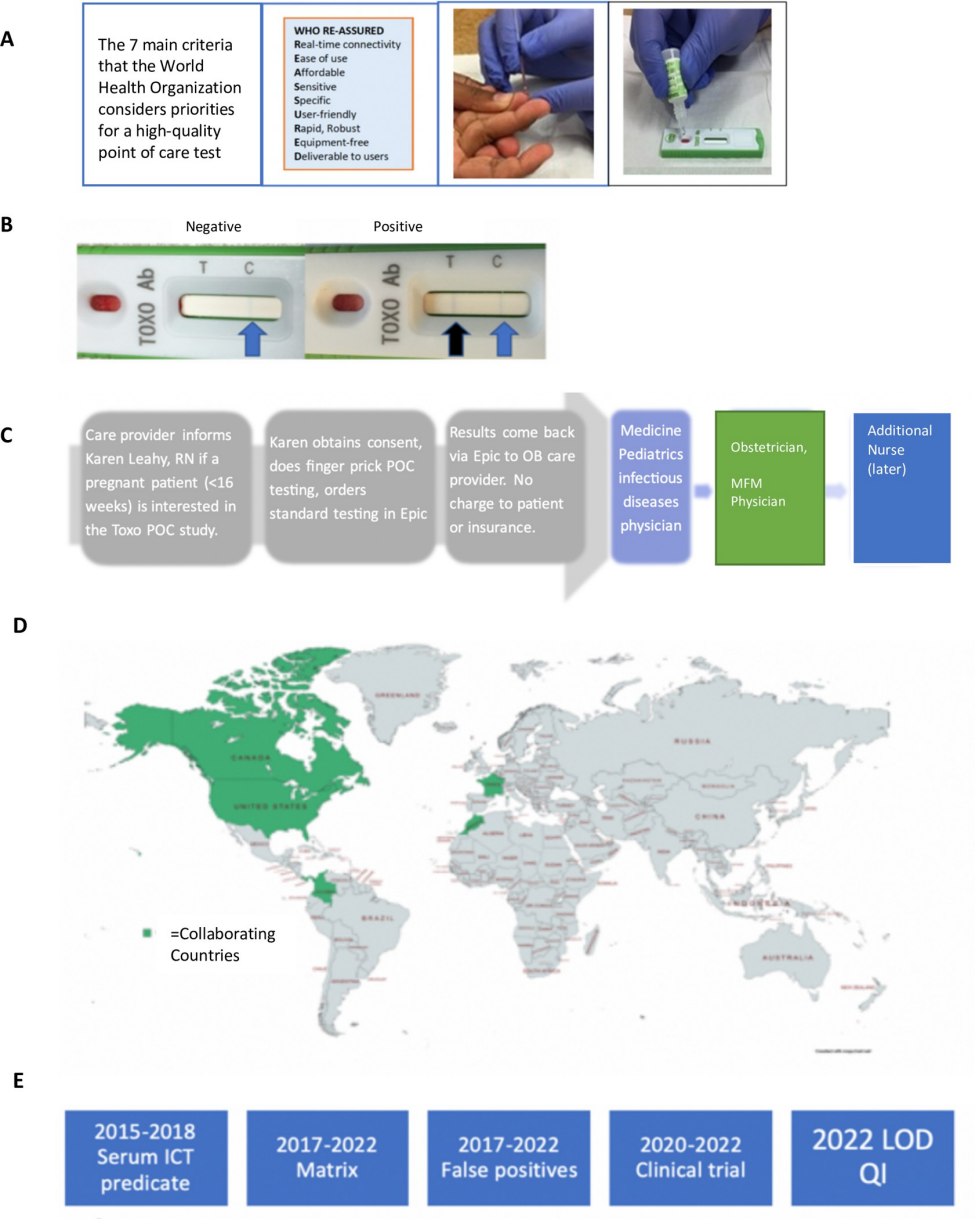
Design, “Roadmap” and Context for Study 1 Performed herein. Photograph of hands and kit from [23] open access license. Map produced in Carta. From [8]. Open Access License.

The design of this Study 1 (Clinical Trials.gov number NCT04474132) and how it is related to earlier work is shown in **Fig 1A–1E.** To begin to implement a reasonable, feasible, low cost workflow for USA gestational screening programs, which has currently and historically been problematic (please see **S1 Commentary** showing those problems for care), a series of 12 studies described herein were performed. A formal clinical trial feasibility study at the University of Chicago Medical Center (UCMC) began in July 2020. The goal was to evaluate a sufficient number of verifiable ICT results to complete the FDA (510(k) clearance and CLIA regulations waiver process. This study involves comparing results of the ICT to an already cleared serum test, also called predicate test (Bio-Rad Bioplex 2200 Toxo Enzyme Immunoassay). Serologic samples for the UCMC feasibility study 1 (ongoing as of February 2024) were obtained from 41 pregnant women, 40 undergoing regular prenatal appointments at the UCMC (23 in first trimester, 12 in second trimester, four in third-trimester) and from seventeen non-pregnant volunteers. Each participant’s whole blood and sera were tested with ICT; Participants’ sera were also tested at the UCMC’s CLIA-approved Clinical Laboratory, which uses an automated Bio-Rad Bioplex 2200 Toxo Enzyme Immunoassay as its FDA-cleared standard predicate test to detect IgG and IgM anti-*Toxoplasma* antibodies. For this ICT feasibility clinical trial, there were three testers in 3 different sites in the University. All discrepant results between ICT and predicate were sent to Remington Specialty Laboratory- PAMF-TSF or Lyon Reference laboratory for confirmation immediately using a panel of tests described elsewhere [27,37]. Specifically, as the FDA guidelines specified, this was carried out to determine whether this new ICT could be used successfully within formal, usually structured, clinical care systems in a University outpatient obstetrical and in-field practice settings. It was compared with the simultaneously performed FDA cleared, standard, predicate test. The ICT with whole blood was read by three readers independently and with sera by two independent readers also at the same times. Three different healthcare providers performed the point of care (POC) test. Each provider tested a minimum of five sero-positive persons. Results were entered into the University hospital EPIC medical record, standard clinical laboratory, and Clinical Research Redcap systems of the University of Chicago Hospitals with ICT results also documented by smart phone photograph.

**Box 1. Roadmap and Milestones Create a Flow Chart of Studies, Analyses, Content, Supporting Figures, Boxes, and Tables.**

**Table:** 

Study No.	Topic	Figures	Boxes	Tables
**1**	Feasibility Clinical Trial	1, 2A	1, 2	1, 2, A^a^
**2**	False positives a-Lyon, b-Paris	2B, 2C	1, 2	1, B^a^ and C^a^
**3**	Acceptability Monthly screening, USA	4	1, 2	1, C^a^, 3
**4**	Pink/Black, All studies analysis, Novel Paradigm	3	1, 2	2, 3, 4
**5**	Time/Cost Analysis		1	4
**6**	Quick Information/Limits of Detection (LOD)	2F, 2G	1, 3	
**7**	Acute Infection Colombia, USA	2E	1, 2	5
**8**	Additional Chronic USA		2	
**9**	Epidemiology studies	5	2	
**10**	False positive/negative AD Bio, Colombia, USA	2D	1, 2	5, 6
**11**	Commentary: Case summaries		A^a^	
**12**	Commentary: Chronology/Context	A^a^, B^a^	1, 2	1^b^, 2 ^b^, 6
Summary	Summary of Results on Roadmap	6	1	

^a^in S1 Commentary;

^b^Line data Links in Tables 1 and 2

This was tested to determine whether this novel, simple test could perform well even during a time with the constraints posed by the SARS CoV-2 pandemic. As the FDA clearance, CLIA waiver and Institutional Review Board (IRB) processes specified must occur, this was a formal Clinical Trial included in Clinical Trials.gov NCT04474132, that would mimic ways the test would be used to identify serologic positivity or seroconversion during pregnancy in the University of Chicago Medicine practice or in other clinical settings that would be suitable to apply for dual FDA clearance and CLIA waiver. This was to formally determine whether this test could perform well and be feasible and easy to use within a clinical trial in a standard obstetrical and other care and research settings in accordance with FDA and CLIA requirements. We were expected to use a standard, comparator, “predicate” test that the FDA had already cleared for use with serum in the USA to detect and confirm presence of antibodies to *T*. *gondii*. There were only three such cleared tests in the USA and we selected the test our clinical laboratory utilized regularly, herein designated “predicate comparator test” so it could exactly replicate the academic practice setting in accordance with FDA/CLIA guidelines. The test cost was lowered for the clinical trial from $650 for the IgG and IgM clinical laboratory test to $13 to facilitate the study. The cost of the predicate testing was paid for by the Seed Fund Award from the Susan and Richard Kiphart Family Foundation, the A.K. Thrasher Children’s Charity and other charitable sources of funds. US Reference laboratory confirmation of test results could not be subsidized and remained over $1600 for two consecutive tests with a full “adult panel” when a positive IgM test was identified. Back up testing was performed for two patients in the Remington *Toxoplasma* Specialty Reference Laboratory and also was performed in the Lyon reference laboratory without charge using Abbott ELISA IgM/IgG. In August 2022 the Abbott IgG reagents also became FDA cleared.

#### Whole blood sample testing protocol for ICT

For each ICT, ~30 μL of whole blood was collected using a 60μl glass micro hematocrit tube filled to half full (by visual estimate) and placed into the well of the device. After placing four drops of diluent buffer in the well and waiting for 20 minutes, each ICT test yielded results that were interpreted by the tester and two additional readers independently reading photographs of the results. All individuals who interpreted results were blinded to the Bio-Rad IgG and IgM test results. Test interpreters determined whether the ICT result was positive (black, positive line and blue, positive control line) or negative (absence of the black, positive line and presence of blue, positive control line). According to the test Instructions for Users (IFUs) its performances are 97.5% sensitivity and 99.7% specificity when used with whole blood.

#### Predicate test protocol

*Toxoplasma* IgG or IgM (Bio-Rad) (Bioplex 2200 automated assay) detects IgG or IgM antibodies against *T*. *gondii* via capture of IgM in solid phase. Microplate wells coated with anti-human antibody chains, a mixture of antigens and monoclonal anti-*T*. *gondii* antibody labeled with peroxidase, the conjugate, are used. Values ranging from 0.8 to 1 were “equivocal”, values > 1 were considered positive for IgM. These tests were used in accordance with the manufacturer’s instructions. When the first false positive IgM tests results were detected Bio-Rad validated proper functioning of the test materials and automated machine at UCMC and re-calibrated and re-standardized the machine. This did not eliminate the false positive results which seemed to be associated with the reagents and system intrinsically.

#### Confirmation of discrepant results for Clinical Trial

All discrepant results between ICT and predicate were sent to Remington Specialty Laboratory- PAMF-TSF or Lyon Reference laboratory for confirmation immediately using a panel of tests described elsewhere [27,37].

### Study 2a: Samples from Lyon Reference Laboratory that had been referred when reported/referred by local laboratories with erroneous positive IgM

A set of 32 samples obtained at the Parasitology Laboratory of the University Hospital of Lyon, France (Institut des agents infectieux, Hôpital de la Croix-Rousse, Lyon, France) were selected for being reported as false positive with at least one first-line, NRL automated assay and confirmed to be negative by a panel of additional tests in the laboratory including Abbott Architect G, M, Vidas G and Bio-Rad Platelia IgM ELISAs. They were in some cases additionally tested at LDBIO Diagnostics using ICT and WB ToxoII IgG which is a gold standard in France [40–45].

### Study 2b. Testing of other samples at Hôpital Bichat, Paris

A total of 558 US serum samples that had been tested in a one week time period at the University of Chicago Medicine Clinical Laboratory, then stored for one week, that would otherwise be discarded were tested at Hôpital Bichat, Paris (Bichat-Claude Bernard Hôpital, Laboratory of Parasitologie, Paris, France [30]). Another set of French samples also was tested at Hôpital Bichat in Paris. Results are being presented in detail in a separate report describing a variety of tests from Hôpital Bichat [30]. Twenty-two false positive NRL IgG and 6 false positive NRL IgM samples were tested in this part of the study. Follow-up without seroconversion and other reference tests were used to confirm that positive results were false positives.

### Study 3: Testing of US Samples in a study to Determine Feasibility and Acceptability of fingerstick in monthly US gestational screening program 2017–2022

#### Practice setting and patient recruitment

This separate study was to determine whether this ICT testing could be performed monthly for pregnant women in an academic obstetrical setting in the USA and whether it would be acceptable for patients and their physicians. This study took place in the outpatient Obstetrics and Gynecology Practice at an urban academic medical center between September, 2017 and September 2018. Patients were identified at their first outpatient obstetrical visit, between 8–12 weeks gestation, by their primary obstetrical care provider. Patients not infrequently attended their obstetrical visit with their partners. They were provided an educational pamphlet [33] and were able to ask any questions. Patients then were offered an opportunity to participate in the monthly screening study and if they wished to do so to sign an informed consent. If the patient indicated interest in participating in the study, voluntary consent was obtained by the research team. All patients who were asked expressed interest and willingness to participate. Original intent of the study was to follow 20 women to term with monthly testing through the sixth week post-partum obstetrical visit.

#### Testing

Each month, at the patients’ regularly scheduled appointment or shortly thereafter the patient was tested with the whole blood-variant *Toxoplasma* ICT IgG-IgM POC test. Methods for testing have been discussed in our previous work [23] and above. Serum was tested with another high-functioning test, i.e., with the ARCHITECT-(Abbott, United States), and /or as an automated enzyme-linked fluorescent immunoassay (ELFA, (VIDAS ImmunoDiagnostic Assay System, Biomerieux, France)) and/or Western Blot-Toxo-IgG and IgM systems (LDBIO diagnostics) performed in Lyon, France and/or Quindio Colombia Reference Laboratories [10,25]. One hundred fifty-five total tests of each type, i.e., ICT on serum and blood and reference lab G and M tests in Lyon were performed. Eighty-eight of these were also tested with Vidas IgG and IgM in Quindio. We also tested an additional 25 participants in the National Collaborative Congenital Toxoplasmosis Study (NCCCTS) and our other studies during this time frame who wanted to participate.

#### Provider participation and patient satisfaction surveys

Providers joined the study as collaborators following an Obstetrics Department Grand Rounds and Obstetrics Sectional Educational informational meeting for those who missed the Grand Rounds. Both informational meetings were presented by RMc. Providers were given the same educational pamphlet that their patients also received. All had the opportunity to ask questions of RMc and learned about various related educational resources [1–3,6,7,32–35,38,46].

As described above under “Patient Recruitment”, providers then mentioned the study to their patients. At the initial and subsequent visits the medical student (JL), Maternal Fetal Medicine Nurse (KL), or PI (RMc) obtained the samples after coordinating with the patient and practitioner at the time of a subsequent monthly obstetrical visit. Providers were told the results they could discuss with their patients.

Surveys, designed to assess patient satisfaction with the gestational screening program were created to use at the end of the study. Responses were based on a 5-point Likert scale, ranging from “strongly disagree” to “strongly agree.” There was also an opportunity for free response regarding strengths and potential areas of improvement for the screening program. The details of the questions are in the results section. Surveys were provided by the research nurse or others working in the study to the study participants at the 6-week postpartum visit or shortly before this visit. Contact with provision of the brief questionnaire was missed for five study participants at the 6-week postpartum visit. All five were asked and two of those five completed the questionnaire at a later time. Surveys were anonymized, so correlation of survey data to individual respondents was not possible. As part of this intent-to-study, as above, we had enrolled 22 pregnant women, and 19 continued monthly. In the Lyon, France reference laboratory, the 155 samples were tested with Abbott ELISA IgG/IgM. When Abbott Architect (France) IgG/IgM had either an IgG or IgM that was positive, backup testing was performed with VIDAS in Lyon laboratory, and LDBIO Western Blot IgG/IgM (IgM performed for three tests at LDBio). In the Quindío, Colombia reference laboratory that uses VIDAS family (Immuno Diagnostic Assay System) as an automated enzyme-linked fluorescent immunoassay (ELFA) test, the last 88 of the 155 samples were tested in parallel.

Additionally, results of evaluations presented in Medical/Scientific congress that were known by the authors including those in submission to another journal was added to the totals in this analysis.

### Study and Analysis 4. Comparison of pink bead used earlier and black bead used in later studies and herein (Study 4) and Collation of Earlier Testing, Bibliographical search, and development of novel paradigm (Analysis 4)

#### Study 4

A comparison of the performance of the pink line/red bead test kits used in early studies and black bead test kits were performed to determine that the performance of the test kits using these different beads were comparable: 1074 serum sample were tested with the pink line/ red bead and black line test kits and Abbott Architect in Lyon (N = 200; Wallon), Marseille (N = 200; L'Ollivier), Ste Etienne (N = 374; Mahinc, Flori), LDBIO, (N = 300; RP). Any discrepancies were resolved with Western blot. This is part of the formal CE Mark, French Reference Laboratories, and FDA/CLIA file submissions of line data that also were reviewed in Chicago.

#### Analysis 4

We collated results of all of our earlier work, both published already [23–27,29–31,35] and other separate studies ongoing at present on related topics that were published separately concurrently [30], and our current work herein. Our earlier work and some of the work presented herein was used to apply for European CE mark which was granted in October 2020. Now that the ICT is CE marked and available in Europe, to determine whether we had overlooked any other study we were not otherwise aware of, we performed a bibliographical search on Pubmed using terms for evaluations of the *Toxoplasma* ICT IgG-IgM test. This was to make certain that we had included all reported tests in our analysis. Only English literature was reviewed. For evaluations found, full text was retrieved and searched for potential false positive samples. Additionally, results of evaluations presented in congress that were known by the authors including those in submission to other journals were added to the totals in this analysis. Earlier and ongoing studies and reports were arranged chronologically and the total collated data are reported herein.

#### Summary diagram of novel algorithm the work presented herein develops

Difficulties we encountered initially in our clinical trial inspired organizing the algorithm we created to prevent problems like those we had to address.

#### Study/analysis 5: Time cost analysis

We found a number of approaches including reference laboratory tests with varying costs, ease of use, and considered whether they meet WHO REASSURED criteria. Advantages and disadvantages of those approaches are summarized in tabular format including a time cost analysis.

#### Study 6: Evaluation of instructional materials for ICT with whole blood at point of care in limit of detection/ quality of instructions (LOD/QI) study in accordance with FDA/CLIA guidelines

The following study was performed to determine the precision of the ICT with samples at the limit of detection and whether never experienced testers could read, understand, perform and interpret instructional material for use of the ICT with whole blood.

### Sample preparation

Samples were prepared in accordance with FDA/CLIA requirements and guidance for instructional material for CLIA waiver for a point of care test (Recommendations for Clinical Laboratory Improvement Amendments of 1988 [CLIA] Waiver Applications for Manufacturers of *In Vitro* Diagnostic Devices ([version of January 30, 2008 –in force and updated in 2020]). A serum sample that had *T*.*gondii* antibody, that was positive at the limits of detection for the *Toxoplasma* ICT IgG-IgM test was used as positive sample. The sample was prepared according to CLSI EP12-A2 User Protocol for Evaluation of Qualitative Test Performance guideline, with the objective of obtaining a sample that would be positive around 95% of the time (defined as positive between 36 to 39 times out of 40 trials, as per guideline instruction). The sample used was a citrate-based plasma obtained from the *Etablissement Français du Sang* (the French national blood bank) with both IgG and IgM for Toxoplasmosis (38 UI/ml of IgG and 63.89 ratio for IgM according to Roche *Toxoplasma* kits) prior dilution into seronegative heparinized blood obtained from venipuncture of a known seronegative person the day prior to dilution. The selected dilution was the 90^th^ (1:89 ratio) as the test was read positive 37 times by an untrained user and 39 by an experienced user at that dilution (LDBIO Diagnostics). Assuming linearity of dosage for the Roche kit, the IgG titer of the final sample was therefore 0.42 UI/ml, and 0.71 ratio for IgM, both below the threshold of 3 and 1 for the Roche IgG and IgM kit, respectively.

Knowing that this limit of detection was determined to occur when this positive serum was diluted 1:89 with whole blood, serum or plasma from a seronegative donor, the study proceeded as follows: Prior to being tested by nine testers, the whole blood from another seronegative donor in Chicago was confirmed to be negative with ICT from fingerstick. Thirty ml of whole blood was drawn from this second seronegative donor by venipuncture and placed into three ten ml tubes with EDTA anticoagulant and gently mixed by repeatedly inverting the tubes. This blood was divided into two parts, one part was to be used to create the negative samples and the second part was to be used to create the “limits of detection positive” sample. Half the blood from the seronegative donor was diluted 89:1 with the previously tested positive serum and gently mixed to create the “positive at the limits of detection whole blood sample”. The ability of an experienced tester to distinguish the negative and positive samples was tested with the ICT and was confirmed. Two hundred microliters of the negative whole blood was placed into each of sixty-three 1.5 ml Eppendorf tubes used in this study for the negative samples. Two hundred microliters of the “positive at the limits of detection whole blood sample” was placed in each of sixty-three 1.5 ml Eppendorf tubes. The samples were assigned and labeled with random numbers between 1 and 14. The code was known only to the scientist who diluted the samples and prepared the labeled tubes and created the unknowns to be tested. A set of unknown blood samples labeled from #1 to #14 was prepared for each of the nine testers along with ICT cassettes labeled with the number between 1 and 14. Each set had the testers initials on each cassette. Each tester had the same samples labeled 1 to 14. The limits of detection, “Quick Instructions” (“QI”) study was then performed as follows: Nine testers were identified:

### Testers

The testers were asked to read and sign the informed consent if they concurred. They were asked to read the information (Quick information, QI) document and complete the top of the data sheet documenting they had read the QI and indicate if they believed they understood its content. They also answered questions about their educational and other ICT experience. The cassettes with initials and numbers along with capillary tubes, laboratory coat, gloves, and data sheet were provided in a work space separate from other testers and with guidance from the written instructions. Informed consent, and signature into a study log were obtained.

Testers were three practicing physicians, three nurse/nurse practitioners, two medical students and one medical resident. They were not experienced with this type of ICT using whole blood or this cassette. None worked as a certified laboratory technician. They were selected to reflect categories of potential users of this test who were unfamiliar with and unskilled with this test. Each tester took the University of Chicago blood-borne pathogens and universal precautions training to work with whole blood, and their competence in understanding and using this material currently was formally documented for the study, as was IRB-required. The ability of three groups of testers with different clinical roles to read the instructions, to perform the test in accordance with the instruction, and to distinguish negative and positive results at the pre-established limits of detection were evaluated.

### Test and analysis

The ability of three groups of testers with different clinical roles to read the instructions, to perform the test in accordance with the instruction, and to distinguish negative and positive results at the pre-established limits of detection were evaluated. This was done to determine whether the instructional material was adequate to teach unskilled users in three different practice type settings. Familiarity with universal precautions and biosafety instruction precautions was documented as described above. The fourteen samples, half positive and half negative were tested by each tester in a blinded manner. The tester and one other reader read the tests. The testers read the cassette at 20–30 minutes after sample followed by buffer were applied to the cassette, and recorded their interpretations on a data sheet. Photographs of the cassettes were taken using smart phones with photography in an area with some natural light. The photographs were provided to the tester to read in a blinded manner. One of the testers was a second reader for 7 of the photograph sets and a laboratory scientist was the photograph reader for the remaining two tester’s photographs including the tester who was the photographer and second reader for the first 7 testers/readers. A skilled physician scientist with familiarity with the test was a third independent photograph reader. The tester, another unskilled reader (other than the laboratory reader who was experienced), and experienced reader all independently read the photographs of the cassette in a blinded manner and their results were recorded. Data from the data sheet then were analyzed.

#### Study 7. Detection of acute infection and seronegatvity in Quindio, Colombia by using ICT

Sera from 22 patients who had recently acquired their *T*. *gondii* infection in Quindio Colombia and 12 patients who were seronegative had sera that were tested with Vidas ELIFA IgG, IgM and with ICT in their Quindio Reference laboratory.

#### Study 8. Additional NCCCTS patients and their families had testing with ICT while at follow up visits in Chicago to add data to determine that antibody present for many years is still detectable by ICT

Between March and December 2018 20 participants in the NCCCTS whose serologic status was known from earlier reference laboratory testing and family members traveled to Chicago. Number of tests were 20 seropositive and 8 family members or other controls for separate studies such as multimodal neuroimaging studies were found to be seronegative but did not have other reference laboratory testing. Time from acquisition of infection was noted, in a similar approach to earlier studies of Begeman et al [24] and Lykins et al [23].

#### Study 9. Use of ICT for Epidemiologic study in Cincinnati

Sera and demographic data from a maternal-infant cohort in Cincinnati were available for 265 women; 264 had data on the variables of interest. Variables of interest included residential address (longitude and latitude), age, education, race, income and pet ownership as part of the original cohort study. Sera were tested with ICT and if positive then were tested with IgM and IgG western blots at LDBIO. A logistic regression model on the results for the 264 samples was used to estimate the ICT *Toxoplasma* infection positive status by including independent variables such as, maternal age, marital status, Neighborhood Deprivation Score (a higher value means more deprived and missing values are extrapolated from 5 nearest neighbors), latitude, longitude, race, i.e., White or not, and an interaction term between maternal age and Marital status.

#### Study 10. Evaluation of lateral chromatography test AdBio that detects anti-*T*. *gondii* IgM and IgG separately

To determine whether a USA made immunochromatography test (called ADBio) would function as well as the ICT or whether samples from Colombia that had very high performance with ICT would function as well with a different USA made test, an additional set of known IgM positive or IgM negative samples was tested. The tests that were used were the VIDAS (Quindío, Colombia) test and another commercially available but not FDA cleared or CLIA waived test that detects *Toxoplasma* specific IgM in the Colombian Reference laboratory. A total of 147 serum samples were included, selected from the biobank of past studies at the University of Quindío in Armenia, Quindío, Colombia. All samples were previously tested using the reference test VIDAS (VIDAS Toxo-IgG Avidity kit; BioMérieux, Marcy-l’Etoile, France). Samples were divided into the following three groups as defined by VIDAS testing: (1) IgG negative and IgM negative (n = 65), (2) IgG positive and IgM negative (n = 55), and (3) IgG positive and IgM positive (n = 27). These groups corresponded to seronegative samples, chronic *Toxoplasma* infection, and acute *Toxoplasma* infection, respectively. VIDAS TOXO IgM (TXM) assay, is an enzyme-linked fluorescent immunoassay -ELFA- (Biomerieux, Marcy-l’Étoile, France) performed in an automated instrument. In VIDAS IgM test, a value is generated for each sample by forming a ratio from the relative fluorescence of the sample to that of the calibrator or a stored calibrator result ("stored standard"). Test values from patient and control samples are compared to a set of thresholds stored in the computer. The thresholds and interpretations are given as follows according to the values of the cutoff index (COI): <0,8 COI = non-reactive; 0,8≤ COI <1,0 = gray zone and ≥1,0 COI = reactive. The AdBio Test (CTK-Biotech, San Diego, CA) uses lateral-flow immunochromatography with a test and control band and goes further with separate bands to differentiate between *Toxoplasma*-IgG and *Toxoplasma-*IgM antibodies. Tests were performed and results were read according to manufacturer guidelines and confirmed by three investigators. Sensitivity, specificity, positive predicted values (PPVs), and negative predicted values (NPVs) were calculated against VIDAS IgG and IgM test results, respectively, using the Vassar Stats Clinical Calculator (http://vassarstats.net/clin1.html).

#### Analysis 11: Representative Case Summaries illustrative of practical clinical problems where solutions are needed and potential utility of ICT

Representative case vignettes with concepts they illustrate were collected and were summarized to illustrate impact and need of this method and its historical context (as illustrated in “**Studies 2a, b, and 10”; Box A in S1 Commentary**). These brief case summaries are from The National Collaborative Chicago-Based Congenital Toxoplasmosis Study and Consultations to the Toxoplasmosis Center and Toxoplasmosis Research Institute. They illustrate representative, frequent clinical problems incurred from false positive IgM tests. Representative examples of benefit and novel utility of ICT in addressing this problem are also presented. A case summary also presents use of ICT for early detection confirming pre-conception infection when sequential samples obtained in the context of *in vitro* fertilization (IVF) were available. Commentary about screening programs and their absence in the USA further place our findings in an historical perspective in S1 Commentary.

## Results

### Study 1, Feasibility clinical trial study performed exactly as the test would be used in practice, 2020 to 2021, demonstrates feasibility, identifies false positive predicate test results and develops new paradigm to help to obviate that problem

A flow chart showing the design and historical context of this study is in **Fig 1**.

**Fig 2:**
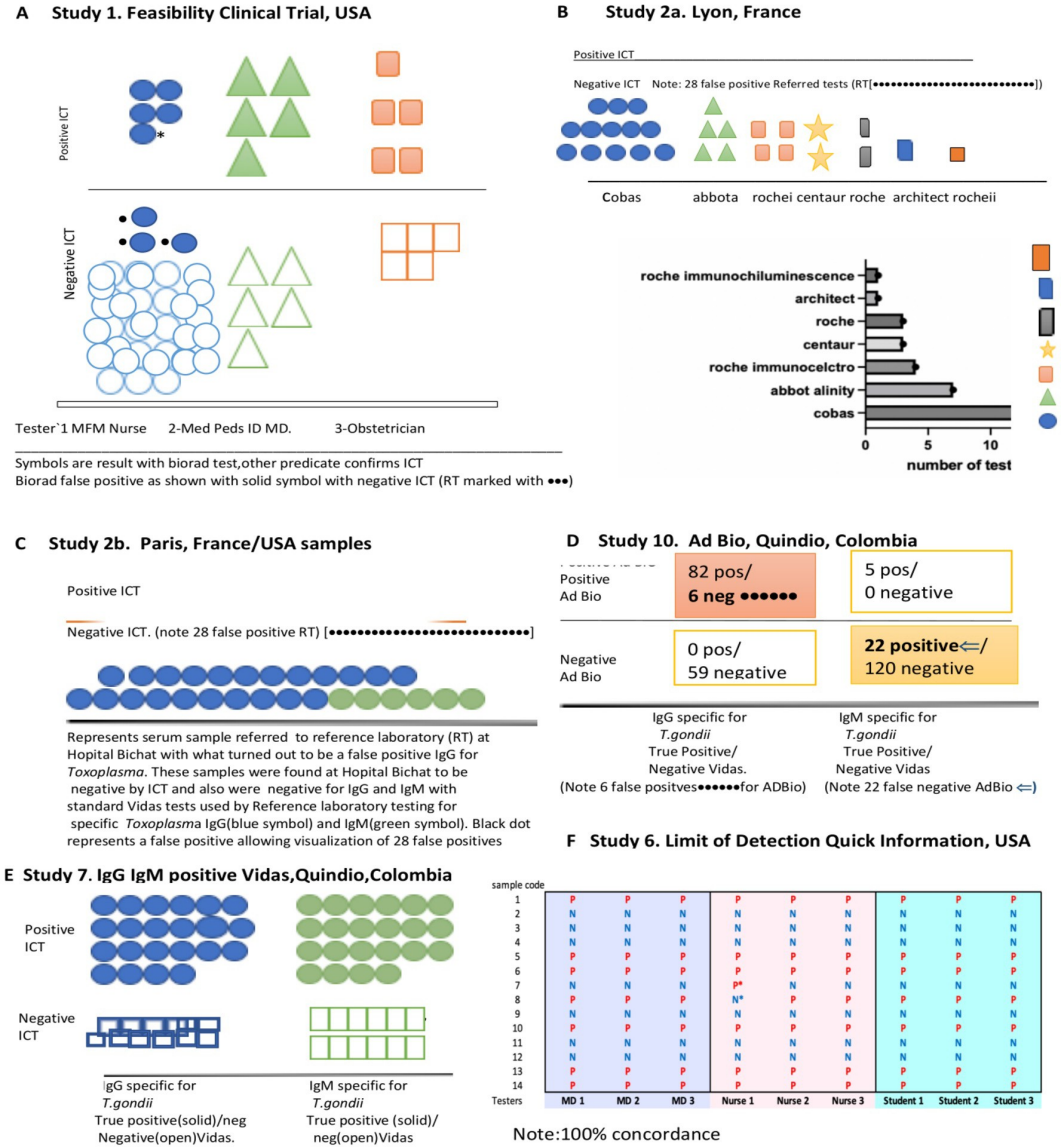
High performance of ICT. A. Clinical Feasibility Trial. Solid symbols represent positive predicate test, open circles represent negative predicate test. Small black dot indicates false positive predicate test. Solid colored symbols indicate a positive test. Open symbols indicate negative test result. Each of the three testers is indicated by a different color in this figure* False positive IgM predicate test for seropositive person. **B. ICT is negative with 32 false positive standard tests in Lyon. C. ICT is negative with false positive standard tests in Paris,** Blue IgG, green IgM. **D. ADBIO, a USA test, suffers from false negative and positive IgM results. E. ICT detects acute infections with positive IgG and IgM in Colombia. F LOD Quick Instructions and Limit of Detection Study 6 Results. Box 3 contains Design and detail of methods and results.** Quick instructions are able to teach 9 testers, 3 physicians, 3 medical students and 3 nurses to use test accurately with 7 samples that are negative and 7 samples at the limits of detection. Details of each study are also in Tables and Figures as follows in parentheses: **Fig 2A** (**Study 1**, **Fig 1A–1E, Table 1 and A in S1 Commentary); B.** (**Study 2a, Table 1 and Table B in S1 Commentary**); **C. (Study 2b and Box 2); D. (Study 10, Table 5); E. (Study 7, Table 5); F. Box 3 and its legend provide detail for Fig 2F (Study 6). Limits of Detection and Quick Information, Box 3-Part A** is the actual **Quick Information** (QI) provided, Photograph of hand and kit from [23] open access license. **Box 3 Part B** shows the **study design, Box 3 Part C** shows examples of test results for two testers, **Box 3D** shows the tabulated results of all the readers in real time and of the photographs). **Box 3 Legend provides Study 6’s. structure and results.** In this study we demonstrate the ease in learning to use a simple, carefully designed test description and that these specific instructions which were prepared in accordance with FDA and CLIA requirements and review (**A**), can function as an effective teaching tool (B, C). This study 6 is also pertinent to demonstrating repeatability and reproducibility of this test. In (B), whole blood was tested at the defined limits of detection of the sample, with 14 negative or positive samples. Positive or negative status is blinded for these 9 independent tester/readers plus additional blinded reader. For this study, these 9 testers were without other training with the ICT and worked in 3 different locations. (B, C). Performance was perfect for each of them after reading this page of instructions (**B, C**). This is consonant with extensive and rigorous testing of use of this ICT in a variety of USA settings, as well as in other countries in many settings with minimal instruction. Detail of objective, methods, data content of Study 6 also is in the accompanying Box. As outlined in the Box, Study 6 shown in 2F, demonstrates that it is easy for physicians, physicians in training, and nurses to learn to use a simple carefully designed description (Quick Information, QI) shown in (A). The QI is about use of the ICT. This study and its results demonstrate that this QI is an effective teaching tool. Also, we find that results with the ICT are robust, repeatable, reproducible, sensitive and specific in multiple settings. **Box 2 and Tables 1, 2** and **3** and **Box 3** and Perspectives section of Discussion, and Supplement (S1 Commentary) contain additional pertinent information and data.

Individual results in the ICT and the predicate test in this ongoing clinical trial study in the context of this design with each of the testers represented by a different color or symbol are in **Fig 2A** and **Table 1** and **Table A in S1 Commentary**. In addition to Study 1, **Table 1** summarizes Studies 2, 3, and 4. Study 4 detail is also in **Table 2**.

**Table 1:** Overview of Chicago formal feasibility implementation, Lyon referred false positives study, and Lyon Architect and Quindio Vidas G and M back up testing studies of Chicago monthly screening study, and comparison of red bead (pink line) and black bead (black line) test kits.

Title (Study No.)	Participant number	Positive / Negative	Testing site	POC tester	Laboratory	Line data Study no. and/or Fig no.
**Chicago formal implementation feasibility clinicaltrials.gov (1)**	1–38	5/33	Hyde Park, Chicago while waiting for MD, Outdoors drive by car, home visit, Pediatric and Obstetrical practice, Examination areas	KL	AA, VT, YZ, back up for US, and Lyon Reference laboratories	Table A in S1 Commentary*; Study 1 Fig 2A
	39–49	5/5	Hyde Park home outdoor site, Pediatric and Obstetrical practice, Examination areas, Conference rooms	SS	AA. VT, YZ	
	50–60	5/6	Obstetrician office in obstetrical practice	MS	AA (VT) YZ, back up for US, and Lyon Reference laboratories	
**Croix Rousse, False positives referred to Lyon (2)**	L1-32	0/32	Community local Referring Laboratories	local lab	Croix Rousse Reference	Table B in S1 Commentary**; Study 2A Fig 2B
**Chicago monthly screening study, Lyon architect, Quindío Vidas IgG, IgM (3)**	C-S 1–20 188 tests	0/188	Chicago Medicine Obstetrics Practice	JL, RMc, KL, YZ	Croix Rousse Reference	Table C in S1 Commentary***; Study
**Pink/Black (4)**	1074 tests	623/451	Ste Etienne, Marseille, Lyon Laboratories		Ste Etienne, Marseille, Lyon Laboratories	Table 2****; Study 4

Legend for Table 1. *Table A in S1 Commentary. Study 1. Design and Data for 3 Testers with 5 Sera-Positive Persons for each tester, Each in Three Settings with Results Showing Their Primary Data in the Chicago Clinical Feasibility Implementation Trial 2020 to 2021. Corresponds to Fig 2A. This Study is Performed in Accordance with FDA and CLIA Guidelines and Regulations.

**Table B in S1 Commentary. Study 2 Part 1 Lyon Reference Laboratory ICT Test Results for Sera from Pregnant Patients Referred by Local Physicians for *T*.*gondii* IgM with Predicate Tests in Local Laboratories and Negative Western Blot as Gold Standard Comparator.Organization and data correspond to Fig 2B.

***Table C in S1 Commentary. Study 3 Shows Concordance of ICT Results in Chicago Acceptability of Monthly Testing In Testing of Sera in Lyon Reference Laboratory Using Abbott Architect and for one person VIDAS IgG ELISA, and VIDAS G and M ELFA in Quindío Reference Laboratory. Initial tests in earlier months were all concordant with Abbott Architect and reported in Lykins et al [28]. This study corresponds to Fig 4 which shows results of USA Acceptability, Study 3.

****Table 2. Study 4 Shows comparability and high performance of pink and black line (red and black beads) test kits.

Line data are at: DOI: 10.17632/dkbzbntpbw.1. **Abbreviations** are the initials of testers; fp (false positive when compared to gold standard testing).

**Table 2:** Study 4 shows validation of the black version of the kit in head-to-head comparisons with pink version studied earlier.

**N of samples**	1074*
**Nature**	**Chosen, French**
**Predicate**	**Pink variant**
**Pos/Neg**	**623/451**
**Sensitivity**	**99.0% [95CI 98.2–99.8%]**
**Specificity**	**100% [95CI 98.9–100%]**

*1074 samples included samples from Lyon (N = 200, Wallon, Chapey), Marseille (N = 200, Lollivier), Ste Etienne (N = 374, FlorI, Mahinc), LDBIO Lyon (N = 300, Piarroux).

There was separate additional comparative testing taking place concurrently in Chicago. There was >99% concordance. Any discrepancies were resolved with Western Blot.

Line data are at DOI: 10.17632/dkbzbntpbw.1. This is part of formal CE Mark, French Laboratories and FDA/CLIA submissions of line data that also were reviewed in Chicago with concordance similarly documented. Architect G, M also performed.

Between August 2020 and December 2021, we performed this prospective clinical feasibility trial, Study 1, in which 43 seronegative and 15 seropositive persons were tested with the ICT using whole blood and sera. The 58 sera were also tested with the predicate test used by The University of Chicago Medicine Clinical Serology laboratory, the Automated Bio-Rad assay. All negative results for the ICT and Bio-Rad predicate test were concordant. This is illustrated by the open symbols below the horizontal line in **Fig 2A.**

This is also shown in **Table A in S1 Commentary** by participant lines for each of the three testers (Maternal fetal medicine nurse, Obstetrician, Medicine Pediatric Infectious Diseases specialist, each indicated in **Table A in S1 Commentary** by separate colors matching the Study 1 design). Any ICT positive results resulted in testing of the sera in Reference Laboratories. Results obtained by all readers for the ICT were uniformly concordant with true positive results found in the Reference laboratories. The specificity, sensitivity, NPV, and PPV were 100%. AUC was ~1. Integration into workflow and acceptability were strong. However. a study related Bio-Rad Bioplex 2200 automated IgM EIA problem arose with this NRL predicate test.

Specifically, results of the ICT, as shown in **Fig 2**, always had at least two independent observers to insure objectivity in their interpretation as well as to avoid any possible bias. Readings were documented by photographs 20–30 minutes after applying the sample and the buffer immediately after the sample. RMc always read the results independently after initial readers and never influenced the readings of the initial readers. Results of all readers were uniformly concordant. Any positive results were re-tested in Reference Laboratories.

As specified by the FDA, and as shown in **Fig 2A** and **Table A in S1 Commentary** the three testers of different professional backgrounds who might be among the categories of users of the test included an obstetrician, an infectious diseases specialist, and an obstetrics maternal fetal medicine nurse. All who tested participants were blinded to the participants serologic status. As shown in **Fig 2A** and **Table 2A**, each tester tested at least 5 persons who turned out to be seropositive and at least 5 persons who turned out to be seronegative. The different sites for testing in Chicago shown in **Fig 2A** and **Table 1A** included triage area and nurses examining area, the obstetricians examining area/office and sometimes in outdoor field settings during the SARS CoV-2 pandemic. Feasibility, accuracy, and one hundred percent correlation between the whole blood point of care test, the ICT with serum performed by a laboratory member, and a predicate and when needed gold standard test were identified.

### Practical problems with the predicate test and USA health care system that arose in this USA Clinical Trial feasibility Study 1 demonstrate a compelling need for novel, practical solutions to enable gestational screening to be implemented into the workflow of USA obstetrical practices

Within testing the initial 6 pregnant participants, we encountered false positive results for two participants in the IgM predicate test and three others later revealing a false positivity rate of 10%. Contacting Bio-Rad about their cut off value for a positive test, re-standardization of the automated machine, checking all reagents did not correct these false positive values above the cut off requiring reporting of a positive predicate test in this practical setting. Further, finding these frequent false positive predicate test results was disturbing for patient participants, providers, and investigators. Correcting the erroneous predicate test data in this **Study 1** with follow up gold-standard testing was time consuming, costly, and would result in delays in care in a real-world setting. The prior 1998 FDA advisory was cautionary but the confounding false positives were initially unexpected. This was because we had earlier used US and French reference laboratory backup testing. We had incorrectly considered that the FDA clearance of the predicate test that we were required to use for this clinical trial would have addressed and solved the problem of false positives.

In this real-life practical model in Study 1 that the IRB and FDA required the resource Reference laboratories in the USA and France provided high quality results providing an excellent solution to the problem of the false positive results, we encountered but they had associated substantial delays, cost and inconvenience. In this Study 1 to define practical functioning of the ICT, this problem with false positives occurred while we as investigators promptly saw the negative ICT result in whole blood and sera testing. We knew the results in the context of our earlier data showed very high performance, sensitivity, and specificity of the ICT with Reference laboratory back up. Our earlier work had demonstrated all negative results were accurate with the ICT. NRL erroneous predicate test results considered to be positive occurred earlier when samples were referred to the USA reference laboratory (27). This occurred when 33 patients with 60 sera were tested in our earlier work [27]. As our Study 1 enrollment reached the first 6 participants with 2 false positives identified by the US and Lyon France Reference laboratories, we recognized that if we could not solve this problem of false positive predicate tests, this de-railed the research study and its longer-term goal of proper testing in systematic gestational screening programs for the USA, and as a model for other countries. For the USA, along with their present high costs in the USA, false positive tests also are substantially harmful.

### New paradigm elucidated by this experience

**Fig 3:**
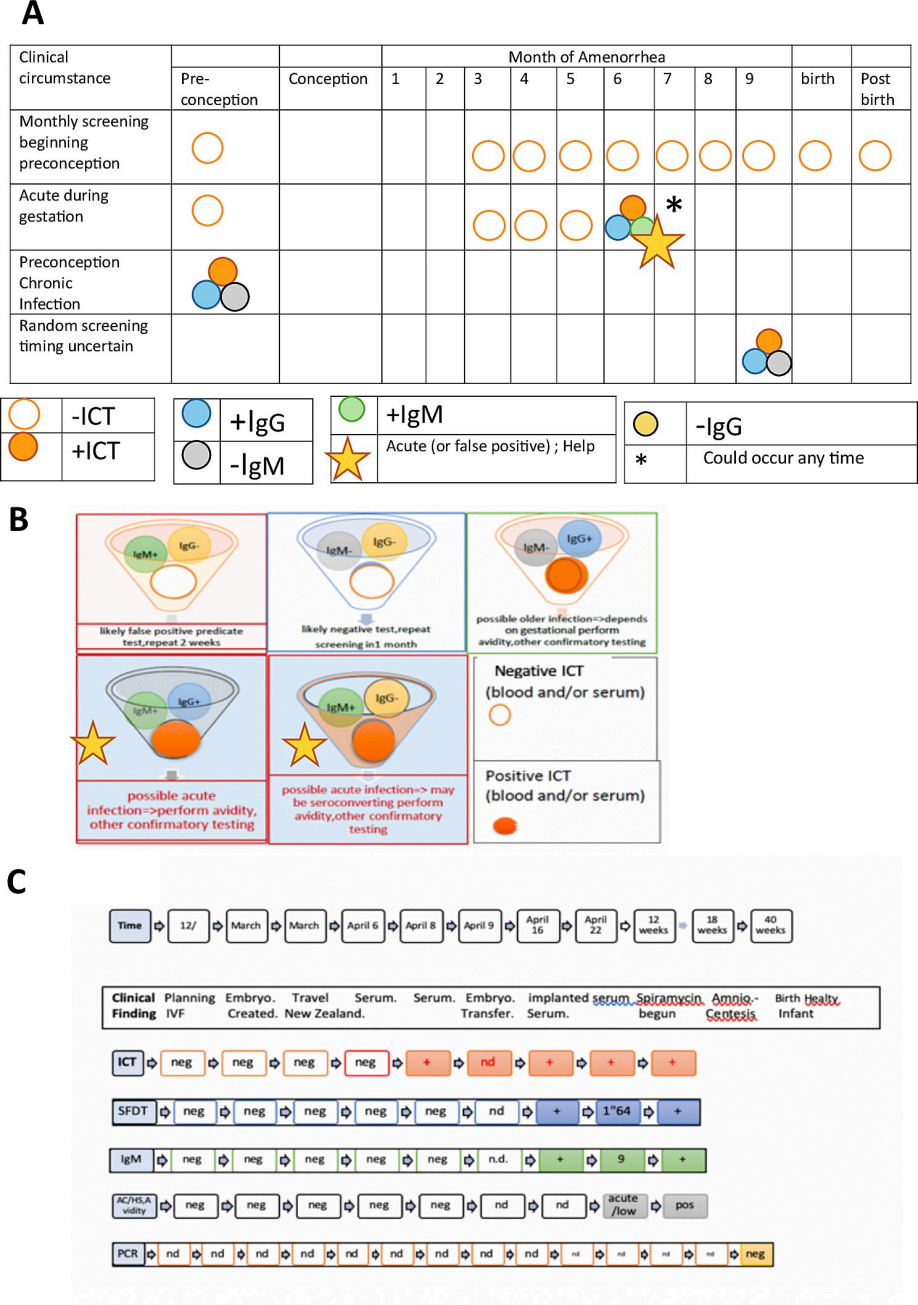
**A. Graphic showing monthly test method.** Use of ICT in NRL as considered originally in and modified from our earlier work (33). Help refers to seeking expert guidance and reference laboratory assistance. **B. Role of ICT in this algorithm shown diagrammatically.** C. **Representative example of ICT detecting very early seroconversion using sera originally stored for another purpose tested retrospectively, showing contrast with false positives.** First pregnancy by IVF. The testing was all done retrospectively after positive IgG, and IgM at ~12–14 weeks gestation was discovered, At this time the ICT was an experimental research laboratory test done prior to studies performed for consideration of FDA clearance, CLIA waiver process and not for clinical care. However, they demonstrated, in this unusual circumstance, sensitivity early in this true seroconversion. This was while the Sabin Feldman Dye test that detects IgG directed against *T*.*gondii* and other Palo Alto Specialty laboratory tests including the IgM ELISAs were negative (antedating the time that they later became positive documenting seroconversion). Details with dates were as follows: **December 2018**. Began planning for IVF-sera *Toxoplasma gondii* antibody negative (Neg). **March 2019.** IVF embryos harvested cryopreserved-sera *Toxoplasma gondii* antibody negative. **March 2019.** Patient traveled to New Zealand returned end of March to USA. **April 6-** Patient sera obtained pre-transfer of embryos. Palo Alto reported negative g and m but there was for the first-time background in IgM ELISA, (~ 0.2, cut off ~2 for an adult), ENZO lab at NYU hospital -commercial lab reported *T*.*gondii* specific IgM positive. Since the commercial IgM was not considered reliable because of known false positives, the serum sample was sent to Palo Alto where this was found to be negative(neg). **April 8** -Serum samples sent to University of Chicago Research laboratory, were tested retrospectively at a later time. ICT was weakly, but clearly positive *; Note this was pre-implantation of the embryo and IVF was in March before infection. This test is binary and not quantitative. **April 9-** Embryo transferred to woman. **April 16**-Sera hormones showed embryo implanted. ICT from this time was weakly positive when tested later, and was a little stronger than in the first sample. Five observers confirmed these readings. This was all with the same lot of the ICT. **April 22-** ICT a little stronger positive. Dye test 1.64, M~9, Avidity low, AC/HS acute. Subsequent sera also positive with significant rise in dye test titer and acute patterns for other tests. Pregnant woman began spiramycin at ~12 weeks gestation when the first IgG dye test, and IgM ELISA were positive. Other sera collected and tested retrospectively. Amniocentesis at 18 weeks, tested at Remington Specialty Laboratory. PCR was negative. All obstetrical ultrasounds were normal. Infant was uninfected. Abbreviations: neg is negative, + is positive, nd is not done. SFDT is Sabin Feldman Dye Test. IVF, in vitro fertilization. “wild screening” is a colloquial term referring to non-systematic testing.

Thus, as we tested the whole blood and sera with the ICT in parallel with testing the sera with the Bio-Rad test, we developed an easy, inexpensive, paradigm shifting approach to solve this problem of false positive tests for the USA. This approach showed the ICT could help to eliminate the problem of false positives both in the clinic and the clinical laboratory. This model **(Fig 3)** was to have a method for diagnosis with a test that meets WHO REASSURED criteria available promptly at the time the test was performed and to have a first backup of positive results in serum rapidly in the local laboratory. Our experience shown demonstrates that this ICT performs properly in clinical practice and field studies. We noted that it could be used correctly by previously untrained professional observers, meeting WHO REASSURED criteria. We found that this also could help to obviate the difficulties caused when a commercial predicate test has false positive result. This was while introducing a novel test that could be low cost and easy to use in the clinic. It became clear from the experience and data presented that this novel test and paradigm could be a useful new method for the clinical laboratory to identify true positives rapidly for this emergent problem/disease. This could help to determine whether there was need for further screening. It could help to clarify whether there was need for emergent care with life, sight, cognition saving medicine that should be initiated promptly while waiting for backup reference laboratory confirmation of a suspected true positive test (**Fig 3).**

### Study 2 with additional testing of erroneous false positive local predicate tests with ICT and gold-standard testing in reference laboratories demonstrates utility of the novel approach with ICT

Then, Lyon and Paris Reference laboratories’ identified erroneous false positive results reported for samples referred for testing from local private laboratories that used commercial tests. Thirty-two samples that were referred to the Lyon reference laboratory from September 2021 to February 2022 from private laboratories because of the detection of isolated IgM in the course of monthly prenatal retesting, which the main system used in France in this context (**Fig 2B and Table 1 and Table B in S1 Commentary**): The tests that had been used included Cobas Roche (n = 21), Abbott (Architect n = 1 or Alinity n = 7), Siemens (n = 3) (**Fig 2B and Table 1 and Table B in S1 Commentary**). None of the additional 32 samples gave positive results with ICT or in the reference laboratory with Abbott Architect despite the erroneous reports of positive IgM results (**Table 1**). Further, in Lyon France, none of the 32 false positive IgM tests with the predicate local laboratory tests used had false positive test results with the ICT or gold standard Western Blot (**Table 1 and Table B in S1 Commentary**). This was using the same referred serum that was tested and reported to be positive from the local private laboratory. These results are included in **Table 1 and Table B in S1 Commentary, and Box 2 and Fig 2.**

**Box 2. All Studies with ICT.**

**Table:** 

Study	Author 1	Year (study no.)^a^ [ref]	Place of Study	Nature of samples	Number of samples Total [pos, neg]^b^	Sensitivity^c^	Specificity^c^	PPV^c^	NPV^c^	AUC^c^	Number of false IgM	Test used	Specificity ICT on false IgM
Chicago feasibility clinical trial: Comparing ICT blood, serum, Bio-Rad clinical lab, back up Reference Lab Lyon, or Palo Alto for suspected false + Bio-Rad EIA automated	Zhou, Leahy, Grose, et al	2023 (1)	USA/ France	**serum/** whole blood	Total: 57/57, [15,42], [15,42] *774 [483, 291]	100%/ 100% *98.55%	100%/ 100% *99.66%	100%/ 100% *99.8%	100%/ 100% *97.6%	1.0/1.0 *0.991	**0 ICT of 5 in predicate Bio-Rad**	Bio-Rad, Architect Tests including Architect, VIDAS G/M, LdBio Toxo II WB and PAMF-TSL adult panel or see Table A in S1 Commentary for backup in reference lab	100%
Performances of ICT toxoplasma IgG IgM test in comparison with VIDAS toxo competition to deter-mine the immune status of patients against Toxoplasma gondii Paris referred false +, true negative ICT	Abraham	2023 (2b) [30]	France	**serum**	Total: 1384 [169:1215]	98.2%	99.8%	98.2%	99.9%	0.99	**0 ICT of 6 M 22 G** **repeated 3 weeks later**	Toxoscreen (MAT), VIDAS IgG/IgM competition, Architect, LdBio Toxo II WB	100%
Lyon lab referred false positives, true negative ICT	Zhou, Leahy, Grose/ Wallon	2023 (2a)	USA/ France	**serum**	Total: 32, [0,32]	NA	100%	NA	100%	NA	**0 in ICT of 32 referral to Lyon**	Architect, LdBio Toxo II WB (see Table 2B)	100%
Chicago acceptability compares ICT blood and serum with VIDAS GM, Architect GM, NCCCTS ICT blood compares serum Palo Alto tested earlier	Lykins, Zhou, Gomez Marin	2023 (3, 7, 8)	USA, Colombia France	**serum**/ whole blood	Total: 196/160 [44,152], [20,140]	100%/ 100%	100%/ 100%	100%/ 100%	100%/ 100%	1	**0 in ICT of 5 G in architect predicate**	Architect, VIDAS, Ldbio Toxo II WB	100%
Colombia Acceptability Also compares ICT with VIDAS GM, or AdBio. Other epidemiological and clinical studies, Cincinnati, Panama	Londono, Gomez Marin, Soberon, Felin, Britton	2022 (4, 9) [35, 47]	Colombia, Panama Cincinnatti	**serum**/ whole blood	Total: 666/783 [256, 410], [366;417]	NA*	NA*	NA*	NA*	NA*	NA*	In clinic, ELECYS IgG/M, VIDAS ELFA, Cincinnati, WBII LDBio 30 pos	ND
High performance of a novel point-of-care blood test for Toxoplasma infection in women from diverse regions of Morocco	El Mansouri	2021 [29]	Morocco	**serum**/ whole blood	Total: 349/632, [112,237], [226,406]	96.4%/ 96%	99.6%/ 99.5%	99.1%/ 99.1%	98.3%/ 97.8%	0.98/ 0.978	NA	Bio-Rad (Platelia) WB II	ND
Contribution of the Toxoplasma ICT IgG IgM test in determining the immune status of pregnant women against toxoplasmosis	Ben-Abdallah	2021 [31]	Tunisia	**serum**	Total: 39 [24;15]^b^	100%	73.3%	85.7%	100.0%	0.867	**13 false positive IgM including 3 also false positive in ICT**	LdBio Toxo II WB	13 false positive IgM in-clueing 3 also false positive ICT
Evaluation of Three Point-of-Care Tests for Detection of Toxoplasma Immunoglobulin IgG and IgM in the United States: Proof of Concept and Challenges	Gomez	2018 [27]	PAMF-TSL	**serum**	Total: 310 [170;140]	100%	98.8%	99.4%	100.0%	0.996	**33 persons 60 samples** **0 ICT of 60 referrals, referred from NRL**	PAMF-TSL: Dye test, IgM ELISA, IgM ISAGA, IgA and IgG ELISA, Immunosorbent assay, ACHS, avidity, Architect, VIDAS	100%
Rapid, inexpensive, fingerstick, whole-blood, sensitive, specific, point-of-care test for anti- Toxoplasma antibodies	Lykins	2018 [23]	Chicago Morocco	**Serum**/whole blood	Total: 244/244 [101;143], [101;143]	100%/ 100%	NA/NA	100%/ 100%	100%/ 100%	1.0/1.0	NA	PAMF-TSL: Dye test, IgM ELISA, IgM Isaga, IgA and IgG ELISA, Immunosorbent assay, ACHS, avidity, Architect, VIDAS	NA
Evaluation of a New Immunochromatography Technology Test (LDBio Diagnostics) To Detect Toxoplasma IgG and IgM: Comparison with the Routine Architect Technique	Mahinc	2017 [26]	France	**serum**	Total: 1002 [339;663]	100%	98.7%	97.4%	100.0%	0.993	**2 in ICT of 23 referrals in predicate**	Architect, ISAGA, western blot	91.3%
Point-of-care testing for Toxoplasma gondii IgG/IgM using Toxoplasma ICT IgG-IgM test with sera from the United States and implications for developing countries	Begeman	2017 [24]	Chicago	**serum**	Total: 180 [129;51]	100%	100%	100%	100%	1	NA	PAMF-TSL: Dye test, IgM ELISA, IgM ISAGA, IgA and IgG ELISA, Immunosorbent assay, ACHS avidity	NA
Evaluation of the LDBIO point of care test for the combined detection of Toxoplasma IgG & IgM	Chapey	2017 [25]	France	**serum**	Total: 400 [99:301]	97%	96%	88%	99%	0.963	NA	Architect	NA
collated data for sera combined	Zhou, Leahy, Grose	2023	multiple countries	**serum total:**	All tests *5633 [1941; 3692], **4967 [1685, 3282]**	99.2% [95CI 99.1–99.2%]	99.0% [95 CI 98.7– 99.2%]	97.5%	99.7%	0.991	**2 ICT of 137 serum samples; 0/3 whole blood with false** **positive predicate**	see above	see above
Collated data for whole blood combined	Zhou, Leahy, Grose	2023	multiple countries	whole blood total:	*1876 [728;1148] **1093 [362, 731]**	97.5% [95CI 97.4–97.6%]	99.7% [95CI 99.68–99.72%]	99.44%	98.78%	0.986	see above	see above	see above

**Note:** This concept for this Venn diagram created by Kanix Wang, PhD is based on the design of the Radially symmetrical five-set Venn diagram originally devised by Branko Gruenbaum and optimised for maximum area of the smallest regions and rendered by CMG Lee. This is an original drawing created for this manuscript but the Gruenbaum design is shown in Wikimedia open license. It is available to others under the same conditions as adapted herein. Each study also is an independent study.

Overall, backgrounds of testers included in the 6 nurses, 7 physicians with varying backgrounds, 4 medical students, 1 medical resident, as well as laboratory personnel. Earlier studies involved additional testers, including those in other countries besides the US. These included many nursing and medical students, nurses, obstetricians, other students and skilled laboratory scientists. Settings included the following locations: obstetrical triage, standard general obstetric and Maternal Fetal Medicine practice examination settings, pediatric specialty clinic settings, laboratory conference room and office settings, in employee health office settings, in ophthalmology private office setting used by NCCCTS for follow up visits, and during the SARSCov-2 pandemic outside (home backyard, car drive by) settings nearby the University that could resemble a home visit setting. The ICT with serum was read by physicians, laboratory personnel, undergraduate, graduate, and medical students. This was in the Toxoplasmosis research laboratory in Chicago, in reference laboratory settings at Stanford, in reference clinical labs in the US, in Morocco, at field sites, and in France in Lyon, Marseilles, Ste Etienne, and in Paris. In some cases, as in the Cincinnati epidemiology study, the ICT was performed in the LDBIO laboratory in Lyon by RP.


**Box 2. (continued). Legend. All studies with ICT:**

^a^ n this column, the numbers in the green font in parentheses indicate the Study number in this manuscript. In this column, the numbers in the blue font in brackets indicate the Reference number in the literature already published.

^b^ In this column, the numbers in the black font indicate data for ICT with serum. In this column, the numbers in red font indicate data for ICT with whole blood from finger prick.

^c^ For calculations: TP = true positives, FP = False positives, FN = False Negatives, TN = True Negatives; Sensitivity = probability that a test will give a correct answer, TP/(TP+FN) x 100; Specificity = TN/(TN+FP) x100; Positive Predictive Value, PPV = TP/(TP+FP) x 100; Negative Predictive Value, NPV = TN/(TN+FN) x 100; AUC = (sensitivity + specificity) divided by 2 (where sensitivity and specificity range from 0–1); NA, data not available.

*In Colombia, in this acceptability study, in this published manuscript, the comparison was between result in whole blood obtained by finger prick ICT and serum tested with ELECSYS-CLIA performed in clinical practice by the clinics. Total:783 (366; 417) whole blood, compared to results from 413 (199, 214) sera tested for those women who returned for serum test. Sensitivity was 96.3% and specificity was 95.9% for ELECYS IgG in this subset of sera. Serum was not tested with ICT. IgMs in sera were also tested with ELECSYS-CLIA IgM performed in clinical practice. Three discordant results between sera and whole blood samples were also tested with VIDAS ELFA G and M. Two acutely infected pregnant women were identified and treated. Using these tests and prior testing results available from earlier testing in clinical practice and follow up of the pregnant women and infants, persistent IgM from infections pre-conception and natural IgM antibodies also were identified. These data are not included in the tabulated overall data analyses because of the differences in study purposes, approaches and methodology, and the absence of serum ICT. Studies in Cincinnati and Panama/USA also had differences in purpose, without any or full confirmation with another Reference test method or substantial methodologic differences. Thus, they also could not be included in the overall calculations. The bolded total numbers are from those samples included in the overall calculations for sensitivity, specificity, NPV, PPV and AUC in Box 2. The performance in the USA hospitals/Reference Laboratory, including field settings, were slightly higher (~100%, 100%, 100%, 100%, 1) and also in the two French Reference laboratories. * In the south of France tabulated with Study 1, concordance for pink line, black line and Abbott Architect IgG and IgM for 774 sera and additional comparison of testing using pink-line and black-line kits for the same additional 300 samples, without knowledge of standard lab serologic results, was >99% (please see foot note to Table 2 for 2x2 Table). Sera from 44 persons who were seronegative for *T*.*gondii* with standard tests and who had malaria, had no false positives test results with ICT.

The following lists tests utilized which have links to full details in Google. All other than LDBIO tests CEMark with applications pending FDA review are CLIA approved (PAMF) or FDA cleared other than Architect IgM: Palo Alto Medical Foundation Remington Specialty Laboratory Adult and Infant panels, PCR, and Avidity; ABBOTT Architect IgG, IgM; Biomerieux Agglutination (IgG only), TXC(G and/or M), BIORAD PLATELIA ELISA IgG, IgM: BIORAD 2200 Bioplex ToRC; ELECYS ROCHE IgG, IgM; LDBIO IgG/IgM ICT; LDBIO Western Blot II IgG, IgM; LIASON IgG, IgM; VIDAS IgG, IgM, ELFA; VIDAS IGG IGM ELISA; with the manufacturers performance evaluations included. The use of these tests with high correlation with ICT is shown by region and test used in that region in the inset into this footnote that follows. The hand-written inset was written at a meeting with the FDA providing the instructions for feasibility efficacy studies in different settings with numbers of participants required for 510K “clearance” and CLIA “waiver” to be achieved.

**Box2_Figure:**
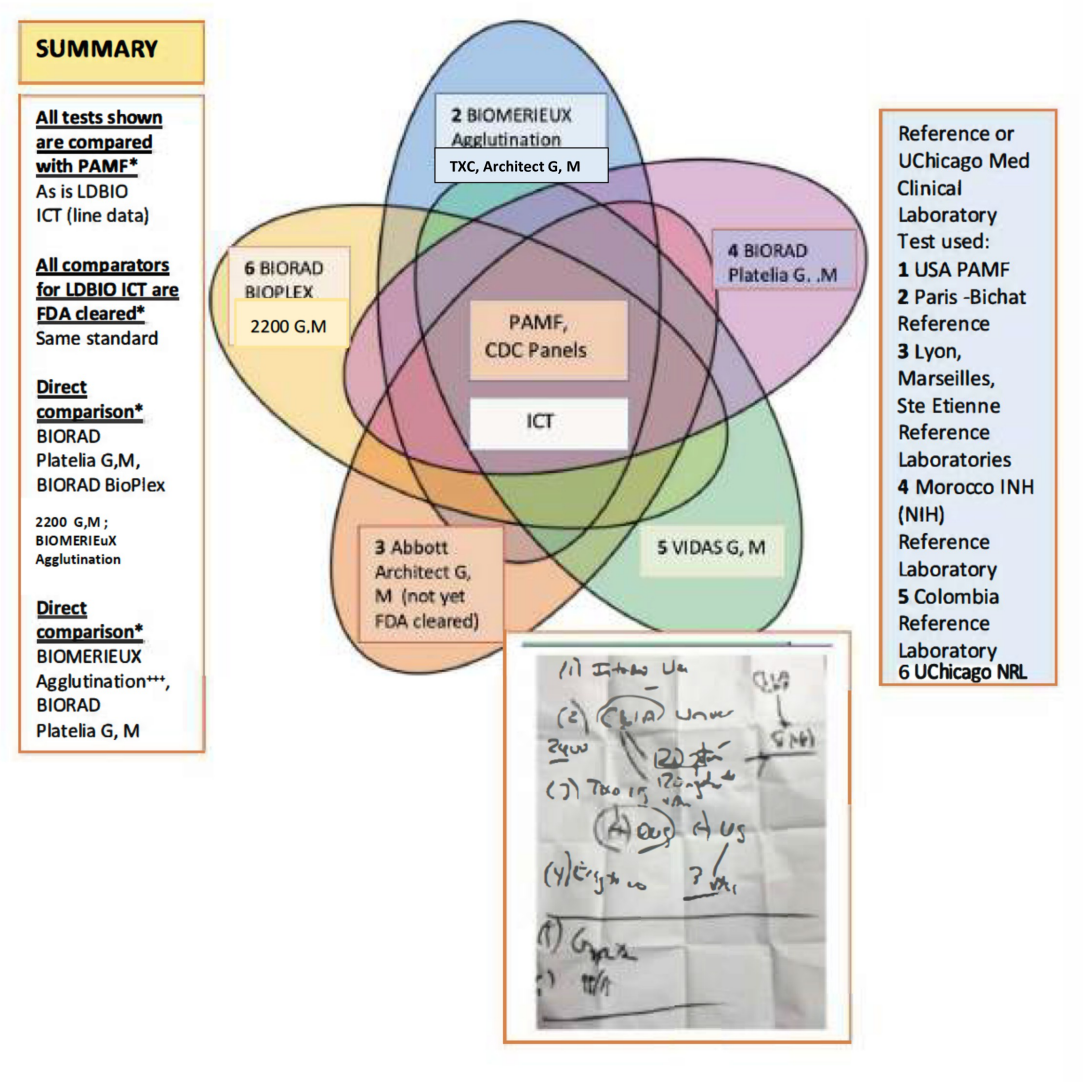

Data summarized in **Box 2** and **Table 3** place the results in the Clinical Trial and monthly screening acceptance studies (**Study 3**), in the context of other ongoing studies and our earlier published work. **Box 2** and **Table 3** address details of studies in the USA and elsewhere. **Box 2 and Table 3** also collate and address studies of false positive IgMs referred to reference laboratories in the USA and France. **Box 2** collates all these studies including those of other countries as a summary of all available results. Sensitivity, specificity, confidence intervals and details of studies are in **Box 2** and, **Table 3 and Tables A, B, and C in S1 Commentary**. Performance of the ICT is high, sensitivity ≥ 97.5%, specificity ≥ 99.4% (serum and/or blood).

**Table 3:** ICT in US and false positives with comparators.

A. Summary of Studies that have employed the LDBIO *Toxoplasma* ICT IgG/IgM test (ICT) on U.S. sera or whole blood samples, with performance results				
**Study**	**Begeman et al.** **(2017)** (1)^**1**^	**Lykins et al. (2018)** (2)^**5**^	**Gomez et al. (2018)** (3)^**8**^	**McLeod et al. (2020-present**^**10**^**; see [website for clinical trials.gov** NCT04474132]
**Comparator test(s) for LDBIO**	Sabin-Feldman IgG Dye Test (gold standard for *Toxoplasma*-IgG in the U.S.); IgM ELISA	Abbott ARCHITECT IgG and IgM assays (Abbott North, Chicago, IL, USA).Positive persons also with earlier dye test and IgM Elisa.	Sabin-Feldman IgG Dye Test; IgM ELISA	Automated Bio-Rad Immuno-assay Toxo IgG and IgM assays (Bio-Rad Laboratories, Inc., Hercules, CA, USA) Bioplex 2200
**Serum**	Yes	Yes^6^	Yes	Yes
**Whole blood**	No	Yes	No	Yes
**Number of U.S. patients represented in samples and serology results** ^**2**^	Infected mother and their newborns, N = 129^4^ Negative IgG and IgM: 51 **Total:** 180 Only 13 were acutely infected at the time of the analysis in this study.	Positive: 67^7^ Negative: 99 **Total:** 166 Only 3 were acutely infected at the time of the analysis in this study	PAMF-TSL Chronically infected: 85^9^ Acutely infected: 85 Negative: 80 IgG-/IgM+ false positives from ELISA (PAMF-TSL): 33 **Total:** 283	Positive: 15^11^ Negative: 43 (4fp) **Total: 58**
**ICT Results for IgM** ^**3**^	TP: NA TN: 51 Sensitivity: NA Specificity: 100%	TP: 3 TN: 99 FP: 0 FN: 0 Sens: 100% Spec: 100%	TP: 85 TN: 113 FP: 1 FN: 0 Sens: 100% Spec: 99.3%	TP: 0 TN: 43 FP: 0 FN: 0 Sens: NA Spec: 100%
**Overall performances of ICT in the US**	IgG+/IgM+ 100% (88/88) [94.8–100%](95CI done using Wilson’s method with correction of continuity) IgG+/IgM- 100% (164/164) [97.1–100%] IgG-/IgM- 99.7% (306/307) [97.9–99.9%]			
**B. Comparison of *Toxoplasma* ICT and comparator predicate tests with false positive results**				
**Source of Sample**	**Test that generate FP or** **“indeterminate” result**	**Number of FPs People**	**Number of FPs tests**	**Toxoplasma IgG/M ICTresult/** **confirmatory test**
PAMF-TSL, Palo Alto, California	IgM ELISA	33 people	60 tests	0/58;0/3 false positive +
Hôpital de la Croix-Rousse, Lyon, France	IgM* see Tables 2 and B and Fig 2B. Bio-Rad Platelia IgM,Abbott Architect	37 people*	32 M; 5 G*	M, 0/32; ++ G,; 0/5
University of Chicago	Bio-Rad Bioplex 2200 IgM	3 people	3	M 0/3 +, ++

**Legend Table 3.**

A. ^1^Prior to this study in the U.S., two studies in France, one by Chapey et al. [24]* and the other by Mahinc et al., [25] tested the LDBIO test and used the Abbott ARCHITECT IgG and IgM assays as comparator. Both studies were conducted before Begeman et al. [23], but Mahinc et al. [26] was published afterward. Chapey et al. [24] found 13 discrepant results between ICT and Architect, 3 being positive IgM Architect and negative ICT without future seroconversion, hence false Architect IgM results and 13 IgM being positive ICT with negative IgG and IgM Architect but those sera were not controlled using a confirmatory test so they could either be false positive for ICT or false negative for Architect. Such false negative Elisa (Architect and others) results with low titer of IgG below threshold has been described elsewhere [25,39–44]. Positive likelihood ratio and negative likelihood ratio were 97% (95% confidence interval (CI): 91–99%) and 96% (95% CI: 92.5–97.5%), respectively. Mahinc et al. [25], worked with 1002 samples mixing prospective (n = 767) and selected samples (n = 235). Among the 1002 samples, 13 were false positive with ICT, as proven by confirmatory Toxo II western blot and Isaga (for IgG and IgM, respectively) and patient’s follow-up. On the other hand, 32 were false positive for IgM (including 25 selected for false IgM). No false negative for ICT while 2 samples were false negative for Architect IgM.

^2^Samples with either IgG+/IgM-, IgG+/IgM+, IgG-/IgM+ according to reference technique as positives. Samples with IgG-/IgM- were considered as negative

^3^As Toxoplasma ICT IgG-IgM does not discriminate IgG and IgM, IgG+/IgG- samples were not included in this analysis. All IgG+/IgM+, IgG-/IgM+ samples (according to reference tests) were considered as positive. All IgG-/IgM- were considered negative.

^4^The Begeman [24] paper used duration since birth of an infected child to determine chronic (>2.7 month after birth, N = 116) and acute infection (<2.7 months after birth, N = 13). IgG and IgM results of positive patients were not available. For positive patients, proof of positivity was obtained because of positive samples (IgG and/or IgM) obtained before ICT. Therefore, sensitivity regarding IgM could not be assessed.

^5^Lykins et al. study [23] also tested 39 Moroccan sera and whole blood samples (33 positive and 6 negative), using the Bio-Rad Platelia Toxo IgG and IgM assays (Bio-Rad Laboratories, Inc., Hercules, CA, USA) as comparators. The ICT (LDBIO) also performed perfectly on these sera, with 33 TP, 0 FN,(false negatives) 6 TN (true negatives) and 0 FP.

^6^78 patients were tested for both serum and whole blood with 100% correlation. Others were only tested with whole blood.

^7^The Lykins paper [23] had 67 positive patients (68 samples) and 99 negative patients (137 samples). However, 64 patients (65 samples) were IgG+/IgM- and not included in IgM analysis. The 3 remaining were IgG+/IgM+.

^8^The Gomez et al. study [27] tested a total of 310 patient serum samples. Of these samples, 100 came from the Centers for Disease Control and Prevention *Toxoplasma* 1998 Human Serum

Panel (CDC-HSP). The precise source of these samples is not available. The other 210 samples, from 183 patients, came from patients referred to the Palo Alto Medical Foundation *Toxoplasma* Serology Laboratory (PAMF-TSL), now known as the Remington Specialty Laboratory. Number breakdown by location is as follows: from CDC-HSP, there were 35 chronically infected patients, 35 acutely infected patients, and 30 negative patients represented. From PAMF-TSL, there were 50 chronically infected patients (50 samples), 50 acutely infected patients (50 samples), 50 negative patients (50 samples), and 33 IgG-/IgM+ patients (60 samples).

^9^All chronically infected samples had IgG+/IgM- (N = 85) and were therefore not included in analysis. All acute (N = 85) samples were IgG+/IgM+. All 60 false + Samples (33 patients) were IgG- when initially tested with the Sabin-Feldman dye test, whereas they were positive when tested with an in-house IgM ELISA test (Table 3 part B). Follow-up of those patients showed no IgG seroconversion, proving false positive IgM

^10^Table A in S1 Commentary. Results are as of 31 December 2021.

^11^All positive were IgG+/IgM-. Four samples were IgG-/IgM+ with BioRad, BioPlex 2200 and negative with ICT. They were all proven seronegative at PAMF-TSL and/or *Toxoplasmosis* laboratory in Lyon, France In Tunisia there were also ~2 false positives with the ICT.

**B.** + Sabin-Feldman Dye Test/IgM ELISA/PAMF, ACHS, Avidity, IgA, follow-up

++ Four test Lyon Panel: BioRad Platelia, Abbott ARCHITECT, BioMerieux [X], [Siemens]

Additional data from another site and reported separately (Table C in S1 Commentary). These confirm these results and independently at another site add an additional 30 French patient samples making a total of 99 persons with negative IgG and with one person with a false positive IgM in the presence of IgG. This is a total N of 99 persons whose sera had false positive IgM by other commercial tests where ICT IgG-IgM was negative.

Current Chicago study:

Patient 3 (26 Aug 2020) had CLIA test result IgG “negative” IgM “indeterminate”

Patient 6 (28 Aug 2020) had CLIA tests IgG “negative” IgM “positive”

Patient 21 (01 Feb 2021) had CLIA tests IgG “negative” and IgM “positive (1.1)”

Initial outside Siemens IgM test was done in another site and was positive.

Additional testing of a set of samples with Architect, Vidas (**Table C in S1 Commentary)** and other tests was performed in an additional matrix analysis in **Study 3** and is pertinent to consideration of false positive test results. Back up testing of another set of sera from a monthly screening and acceptability of the monthly screening program was performed in the Lyon France and Quindio Armenia Colombia Reference laboratories (**Table C in S1 Commentary**). In these reference laboratory settings as well as in the Paris Hôpital Bichat reference laboratory [30] false positive test results were less problematic (**Fig 2C** and **Table C in S1 Commentary and Box 2**, [30]) than in the clinical trial. There were no false positives in the Quindio laboratory Vidas testing and only one patient with multiple consecutive IgG false positives in the Lyon Architect tests.

### Study 4 Comparing kits with pink and black bead and Analysis 4 placing US ICT in the context of other ongoing studies, including Study 3 and previously published studies, demonstrates high performance

One thousand seventy-four serum samples were tested with the pink (red bead) line and black line test kits and Abbott Architect in Lyon (N = 200; Wallon, Chapey), Marseille (N = 200; Lollivier), Ste Etienne (N = 374; Flori, Mahinc), LDBIO (N = 300; Piarroux, Limonne) with >99% concordance. Any discrepancies were resolved with Western blot. (Links to line data). This is part of the formal CE Mark, French Reference Laboratories, and FDA/CLIA file submissions of line data that also were reviewed in Chicago with concordance similarly documented (**Table 2**).

### Analysis 4 Bibliographical search confirms high performance and that data analysis herein includes all published studies

As the ICT is now commercially available following CE Mark approval in Europe, we also used a bibliographic search to attempt to identify results with which we might have been unfamiliar or with inferior performance of the ICT. **Box 2** details all studies performed to date: Number of persons (N), Sensitivity (Se)/ Specificity (Sp), country of samples, N of false positive IgM, ICT results on false positive IgM and confidence intervals, testing for serum and whole blood are in this Table. To date all studies have involved the authors of this manuscript. There were no additional studies identified that have been reported to date. **Table 3A** and **3B** highlight the data from the USA where multiple sequential studies consistently showed very high performance of the ICT with both sera and blood from fingerstick, and studies addressing M positivity.

**Overall, a total of 4967 sera, 1685 positive and 3282 negative, and 1093 whole blood tests, 362 positive and 731 negative tests have been performed, including all published, ongoing studies and those herein. Additional testing in an acceptability study in Colombia added another 413 whole blood paired with serum samples with high performance not included in the total. 4967 total sera (1685 positive and 3282 negative) and 1093 total (362 positive and 731 negative) whole blood finger prick samples were tested in matrix analyses correct for calculating sensitivity, specificity, NPV, PPV and AUC. There was high sensitivity and specificity, 99.2%/99.0% for serum and 97.5%/99.7% for whole blood** (**Box 2**). **Overall NPV is 99.7% (serum), 98.78% (whole blood), PPV is 97.5% (serum), 99.44% (whole blood). AUC is 1 for USA and 0.99 for serum and 0.99 for whole blood for all the tests calculated worldwide.**

### Study 2 a and b and earlier overall performance of ICT with NRL tests demonstrates that false positive IgM also is high

Overall, including our own results herein, we found 137 samples with false positive IgM in at least one NRL technique also tested with ICT, among which 132 were found negative in ICT. The specificity of ICT for false positive IgM was 96.4%. Three samples were from IgG negative pregnant persons in Chicago. Two seropositive persons also had false positive NRL IgM, was not found in Reference laboratory IgM tests. In addition 22 false positive IgG and later 6 false positive IgM results were correctly identified by ICT at Bichat-Claude Bernard Hôpital, Laboratory of Parasitologie, Paris, France [30] (**Fig 2C and Box 2**) and 5 false positive IgG identified in the predicate Abbot Architect in the University of Chicagomedicine samples tested in Institut des agents infectieux, Hôpital de la Croix-Rousse, Lyon, France (**Tables 1 and B in S1 Commentary, and Box 2**).

**Study 5 comparing** **time, cost of tests and approaches demonstrates ICT time/cost savings and aids in eliminating delays,** Approach and an analysis of relative time and cost is in **Table 4**. The ICT is substantially time and cost saving,

**Table 4:** Approach using ICT to gestational screening is cost and time-saving.

A. Approach to screening			
Serology and timing		Result	Course of action
Serologic tests pre-conception		Negative	Screen beginning in first trimester before 14 weeks, monthly
Serologic tests positive pre-conception		Positive	Gestational screening not needed
Serologic tests see seroconvert in gestation		Negative to Positive	Treat without delay
Wild screening positive result		Positive	Expert consultation, often nuanced
**B. Cost and Time saving just for fingerprick, all sample handling, reporting, and billing for ICT at Point of Care versus Predicate testing**			
**Parameter**	**Point of care test**		**Predicate test**
Time for test	<2 minutes to perform 20 minutes to read		Days
Location	POC		Requires Lab
Time to report	Minutes		Over days Multiple Levels
Cost	<$10		>$500 per test in the USA
Participant preference	Yes		“Ok but POC is preferred by some”
False positive results	Very rare; high sensitivity and specificity		Not infrequent; problematic
Solves problem of false positives	Can be very helpful; also as back up test in lab		Can cause problems in clinical lab

### Representative case summaries illustrate problems in care that false positive tests can cause and utility in identifying seroconversion in infection acquired prior to conception, called study 4b and “analysis 11” in methods)

**Box A** in **S1 Commentary** has brief summaries of some consequences of false positive and negative results in ongoing cases in USA clinical practices. These provide further evidence of problems that false positive test results cause, and additional practical operational difficulties encountered in the USA.

**Fig 3 (Study 4b)** shows this new approach diagrammatically and its exquisite sensitivity and therefore utility in identifying infection prior to conception. In **Fig 3C** the ICT detected seroconversion a day earlier than the Sabin Feldman Dye test and IgM ELISA in the USA Reference laboratory. There are a number of examples of patients who developed M alone then M and G [26]. In the Mahinc et al study ([26], **Box 2 and Table 3**) there were 50 serum samples from 24 women for whom there were 17 samples with IgM only and 33 samples with IgM and IgG; ICT was positive for all samples except one that had a borderline IgM ISAGA of 5 for a patient who later was found to have acute *Toxoplasma* infection. There were also another 144 acutely infected persons identified in the USA, France, Morocco and Colombia all identified as positive with the ICT [24,25,27,29,30] **Box 2 and Table 3**. It was unusual, however, to watch seroconversion with as much precision in narrow time intervals so early in infection as shown in **Fig 3C.**

**Box A and Tables A and B and Figs A and B in S1 Commentary** contrast the current status and consequences of congenital toxoplasmosis at earlier times in France and continuing to the present in the USA. This illustrates that the ICT and gold standard back up testing can help to solve a substantial health care problem. This is both in a historical context and at present, with potential spillover benefit for care for pregnant women and their families.

### Study 6, testing ability of written instructional materials to facilitate clinical use of the ICT by healthcare practitioners not skilled in using the ICT in a limit of detection quick information study per FDA and CLIA instructions, demonstrates high performance

Moving toward implementation, ability of written instructional material to be used in clinical practice with samples at limits of detection for positive whole blood samples and negative whole blood samples was found to have perfect performance. This perfect performance after they read the Quick Information (QI) materials was for all the 9 “blinded” readers and testers (3 nurses, nurse practitioners, resident and 2 medical students, and 3 practicing physicians) who were not previously trained to use this ICT (**Fig 2F and Box 3**). It was for all users and all samples when the cassette was evaluated in real time and when the smart phone photographs were evaluated for all 14 unknowns at the limits of detection samples (**Fig 2F and Box 3).** There was reproducible, replicable, robust, perfect performance in Study 6, with additional un-trained users reading the Quick information page of instructions as their learning material. In this setting, the ICT also worked perfectly with whole blood at the limits of antibody detection by the 9 users reading test kits in real time and photographs of those tests subsequently.

**Box 3. presents detail for CLIA required Study 6 that has overview of results summarized in brief in Fig 2F: Overview and detail of objective, methods, data content, and conclusions of Study 6 performed in accordance with FDA and CLIA regulations**

**Box3_Figure:**
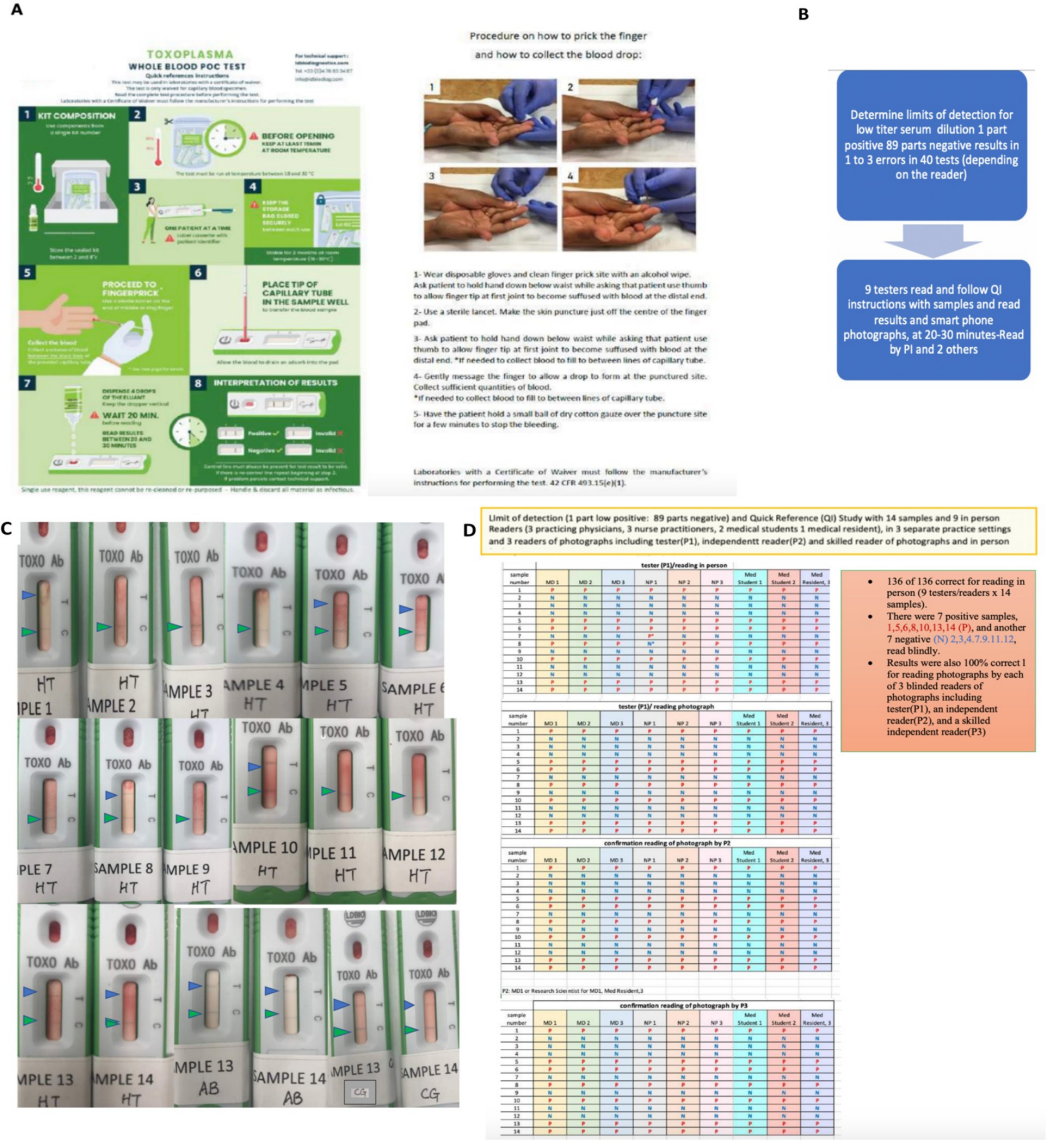


**Box 3. Continued.**

**Legend for Box 3.**

The Objective of Study 6 was to determine whether multiple relevant users could effectively learn from a “Quick Information (QI)”, single page with two sides, in accordance with FDA/CLIA regulations.

A.QI, regulatory processes, and participant eligibility. QI shown in A is not only an instruction page about how to perform a finger-prick but part of a specific required experiment germane to Federal regulations. These regulations require a study with experimental testing at the “cut off” limit of detection using this brief stand-alone QI (A). The FDA and CLIA required creation of and testing this QI in 2020, and iteratively reviewed the versions of this QI until it complied with all their guidelines. This review culminated in creation of (A) that was ready to test whether it could be an effective stand-alone learning tool for use of the ICT. Then multiple levels of IRB and research office approvals of this page and study were required. To comply with IRB approval, testers were asked to complete a suite of safety courses and tests providing information about blood borne pathogens and Toxoplasma. Certifications of completion of this course and testing had to be received by the IRB before beginning. Volunteer participants in 3 relevant categories of care providers who might use an ICT in the future were sought by word of mouth. No compensation was provided. An informed consent was signed. A log page with specific information and questions about eligibility was completed. (B) i.

Preparation of samples: A serum from a French blood bank was identified after testing the initial serum to know its IU. Then, in France the “cut off limit of detection” dilution was defined. This was a dilution that had to be found when tested to be incorrect one of a hundred times it was tested when characterized at multiple dilutions. Dilutions were tested 100 times each by an experienced (RP) and untrained user. A dilution of 1:89 was defined as the “cut off” dilution to be used to create the positive whole blood samples for the testers. Then, this serum was sent to the United States where the test had to be performed on 14 samples by each of the relevant US care provider personnel in the following study: Whole blood samples, either negative or the negative containing a 1:89 dilution of the French serum were prepared at the “cut off” dilution (called “positive”). Then the samples were aliquoted into separate tubes. Then a random number table was used to assign a number from 1 to 14 to each tube of blood. There was one set of numbered tubes for each tester. Each numbered tube contained either a negative or positive sample. Nine identical sets of tubes with the assigned numbers were prepared with one set for each tester.

B,C. i. Testing. After signing an informed consent pre-approved by the IRB (ethics committee), answering a set of questions concerning their background and confirming eligibility, being given a data sheet approved by the IRB, FDA/CLIA to complete, signing a log book, the testers each received a container with materials to use in a designated work space. In this container they received appropriate PPE, a barrier for the work space, a container to dispose their work materials, their set of samples in a small test tube rack, their own data sheet, a pen to record their results, a QI page, cassettes, buffer, marked capillary tubes, and a timer. They were then each able to begin testing these samples in sites approved for this testing and with appropriate PPE for handling human blood samples. The testers did not receive instructions other than their QI instructions. Testers were separated in time and space from each other and could not talk with or watch each other. When they completed their readings and recording of their results, a photographer documented the appearance of the cassettes at 20–30 minutes after loading the cassette with sample according to instructions. In this test, per FDA/CLIA instructions, there was no fingerstick performed. The testing first included two testers who checked the negative and prepared positive initially. They found discrimination of negative and positive samples was readily accomplished. Then, the blinded 9 testers, testing the replicate samples, performed the testing. This was to address who can learn to perform this test easily, reliably and how well with the QI, questions about reproducibility, repeatability, testing of learning and an objective, rigorous proof of results.

B, C. ii. Evaluation of photographs: The same blinded reading process of photographs provided to each tester also took place. The only person who knew code for labeling of tubes was the scientist who prepared them. No tester knew the code or what tubes contained.

D. Data and analysis: Concordance of tube contents and tester readings of cassettes in real time and photographs was 100%. This provides evidence that multiple users of various relevant backgrounds can learn to use this test with samples with IU at the established limit of detection. This is the US CLIA FDA process, and without it, this test cannot be available in the US. This testing demonstrated that physicians in training, in practice, and nurses can learn to use the ICT in 3 physically separate settings from reading the QI. This study also demonstrates reproducibility and repeatability of testing, in the results at the point of test with the ICT. It is also included to show the relevant user learning and testing in this mandated study, in addition to the QI showing how to do the finger-prick. Additional information in the new “Perspectives” section also presents data from over 1500 fingerstick tests with close to 100% accuracy in many diverse settings by many testers in 4 countries with varied backgrounds. In addition to this testing in Study 6, we include information about the wide variety of testers, settings, locations of testing, levels of education of testers.

In Conclusion, we demonstrate that it is easy for physicians, physicians in training, and nurses to learn to use a simple carefully designed description of use of the ICT and that this QI is an effective teaching tool. Also, we find that results with the ICT are robust, repeatable, reproducible, sensitive and specific in multiple settings. Box 2, Perspectives section of Discussion, and Supplement (S1 Commentary) contain additional pertinent information and data.

### Study 3 demonstrates feasibility and acceptibility of monthly gestational screening with ICT

#### Overview

**Fig 4:**
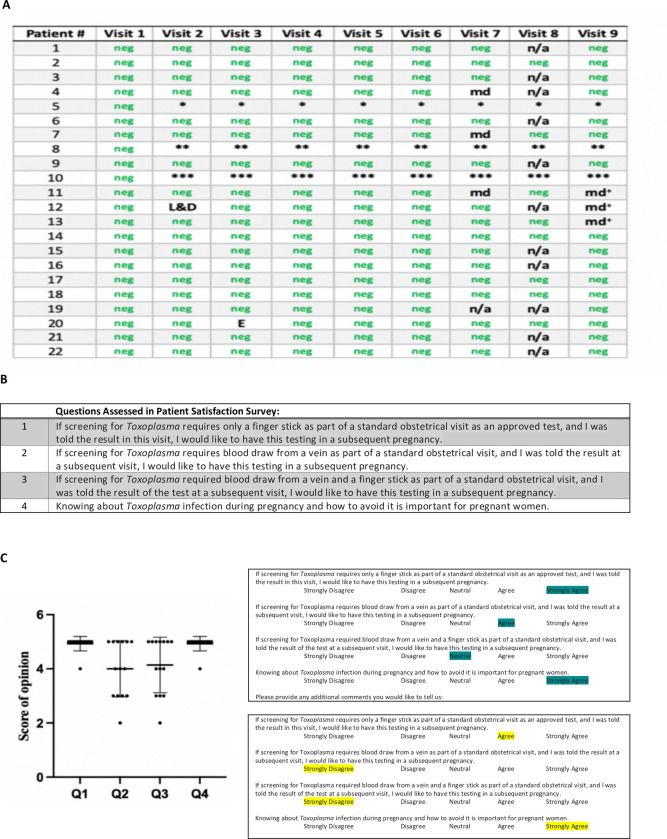
Feasibility and acceptability of monthly screening in U.S. Academic obstetric practice. Abbreviations in A. **“neg”** indicates negative results in tests. Note congruence of ICT and Reference Laboratory test conclusions. ^*^ Elective termination secondary to fetal anomalies, ^**^ Spontaneous abortion, ^***^ Elected to leave the study after first test time due to traditional beliefs regarding having blood drawn. “md” indicates a missed appointment, as patient did not attend her regularly scheduled appointment. Came at alternative time and we did not connect for testing and/or survey. “n/a” indicates not available. “L&D” indicates that the patient’s monthly test was missed due to concern for premature labor, which resulted in a visit to the emergency department and then labor and delivery. “E” indicates an equivocal result, in which a barely visible band appeared which was not reproducible upon photographing the test. Per manufacturer instructions, this test was interpreted as negative. +Survey sent later, not included on graph as was not at initially planned time of six-week postpartum visit^**a**^. **A. Times and results of monthly screening for each participant. B. Survey questions and Likert scale.** n the survey, number responses were considered as follows: strongly agree was 5, 4 was agree, 3 somewhat agree, 2 was somewhat disagree, 1 was strongly disagree **C. Results of satisfaction survey for 14 participants**. Each respondent’s answer is represented by the circles in the figure. The mean is indicated by the horizontal line, with error bars indicating standard deviation. In response to questions 1 and 4, 13/14 respondents indicated that they “strongly agree” that they would pursue testing for *T*. *gondii* in future pregnancies with POC testing, and that knowledge of *T*. *gondii* is important for pregnant women. Results were more mixed if testing required venipuncture, as indicated by the responses to questions 2 and 3, but most agreed that the test was important, would have it again in a subsequent pregnancy, and would recommend this to family and friends. It was noteworthy that other family members such as fathers of the fetus asked to be tested, relevant to the possibility of retinal disease. Right panel showed results after the time of the 6 week post-partum visit. `We did not conduct a formal survey at the time; however, we began with two providers, and other providers in the practice asked to join with their patients. Providers continued in the further analysis of the ICT as it moved toward clearance and waiver.

Early in the work with the ICT in 2017 to 2018 we performed this study to determine whether monthly gestational screening would be feasible in a research study setting (**Fig 4 and Table 1 and Table C in S1 Commentary**).

Results of the testing did not enter standard medical records or the EPIC system at that time and the testing took place earlier than Study 1 but was completed after that study. This study was initiated before the clinical trial and led to the initial meeting with the FDA when a program officer from the Thrasher Foundation emphasized the importance of FDA clearance for the test to be useful to help patients in the USA. We also asked participants at its completion whether these participants felt it was important and comfortable to have knowledge about *Toxoplasma* in pregnancy and whether they would want serologic testing and/or the finger stick point of care test in subsequent pregnancies if it were approved in the USA. The intent was to determine whether screening might be acceptable in standard academic obstetrical USA practice: Some parts of the study, e.g., the questionnaire and additional backup testing were performed in 2020. Results for the initial tests for the participants’ visits were included in the earlier 2018 publication [23]. Thus, numbers included for this study in the cumulative total of tests were subtracted from tests shown in **Fig 4** and for this entry in **Box 2.**

### Patient characteristics and testing details

Patients were all identified at between 8–12 weeks gestation. Patients had a median age of 31 years (range: 24–40 years). Seven of the 22 participants were nulliparous, while the remainder had been pregnant once or twice before. None reported having been tested for *T*. *gondii* infection in the past. Participants were enrolled in the study between September and November, 2017. The study initially concluded in September, 2018 with the birth of the last participant’s child and 6-week post-partum visit. Because five mothers were missed by our study group at their 6-week postpartum visit, an anonymized questionnaire was provided in 2022 for those participants. This was considered separately in our analyses. Patients were tested at monthly intervals after their initial enrollment and tested until their 6-week postpartum visit. A small subset of patients (3/22) were withdrawn from the study: One individual underwent elective termination due to fetal anomalies. One participant suffered a spontaneous abortion. The third patient chose to withdraw from the study due to traditional beliefs about dangers associated with venipuncture. No patients (0/22) had evidence of prior infection with *T*. *gondii* upon their initial testing with the whole blood POC test, and none seroconverted during gestation. One participant had a faint band in the ICT suggesting the possibility of a positive test on one test, but this was only visible to the naked eye and could not be independently confirmed with photography. Per manufacturer instructions, this test was interpreted as negative. There was 100% concordance between testing interpretations of the POC test and confirmatory testing, including the ARCHITECT/Vidas/Western blot systems and the serum-based POC test variant, commercially available and now CE mark approved in France. The course of gestational screening for each participant is presented in **Fig 4** and **Table C in S1 Commentary**.

### Patient perceptions of POC gestational screening are favorable

Initial response of patients and their families to educational materials screening was positive. No patient declined to participate. Patient participants asked whether other pregnant and non-pregnant family members and friends could join. Some fathers asked to be tested to know their own serologic status and if they might be at risk of retinal disease if infected. At the end of the consecutive screening tests, we administered the patient preferences survey to an available subset of the cohort (14 in total) at their 6-week postnatal visit. Two of those participating women who were missed completed the questionnaire in 2022. The POC testing and screening for acquisition of *T*. *gondii* in gestation was well received by all participants. In the post testing survey 100% strongly agreed with the value of education and testing and viewed the point of care fingerstick testing favorably. 100% agreed they would have testing in subsequent pregnancies and advise a friend or family member to do so. Testing by venipuncture and send out testing was viewed less favorably in contrast (p<0.05 at 6 weeks). Specifically, on average, patients appeared to prefer finger-stick POC screening to venipuncture, (POC- 4.93 ± 0.27 vs venipuncture- 4.00 ± 1.04) (**Fig 4B and 4C**). More than 100% (14/14) of enrolled patients “strongly agreed” or “agreed” with Question 1 that they would like to have POC testing in a future pregnancy if it involved ICT by fingerstick if this did not require monthly venipuncture. In contrast for Question 2, only 9 of 14 (64%) “strongly agreed” or “agreed” with screening with venipuncture (Chi squared equals 6.087 with 1 degree of freedom. The two-tailed P value equals 0.0136). 100% of patients “strongly agreed” that knowledge of *T*. *gondii* infection and ways to avoid exposure were important for pregnant women. The two participants who responded in 2022 also agreed that screening was worthwhile but only would want to do this by fingerstick and not venipuncture, a similar pattern. (**Fig 4C right panel**). In summary, in this post testing survey at 6 weeks post-partum or several years later, 100% (16 of 16) of participants “strongly agreed” or “agreed” with Question 1, having and recommending other pregnant women have finger stick ICT monthly during gestation. In contrast, for Question 2, 56% (9 of 16) of survey respondents “strongly agreed” or “agreed” with having venipuncture and send out testing in subsequent pregnancies and recommending this for other pregnant women (Chi squared equals 8.960 with 1 degree of freedom. The two-tailed P value equals 0.0028). All agreed that education and testing are valuable for obstetrical patients with their providers.

### Provider perceptions of POC gestational screening are favorable

There was not a formal questionnaire for providers. Rather, level of interest and enthusiasm was reflected by the following: All providers remained involved in the study with their patients. Additional providers in the practice noting and learning about the ongoing study with the initial providers asked to join with their patients. Those still practicing at the University of Chicago at a later time collaborated in the subsequent clinical trial **Study 1** presented herein. These objective measures demonstrate provider satisfaction. All providers indicated verbally that they found the finger-stick testing and monthly screening a constructive addition to their practice. The rapidity of obtaining the results was viewed positively.

### In Studies 4b, 7 and 8, ICT detects early seroconversion and distinguishes additional seropositive and seronegative samples in USA, France and Colombia

We noted, as shown in **Fig 3,** the ability of the ICT to detect very early seroconversion in a study of sera obtained at narrow intervals to monitor hormone levels during *in vitro* fertilization (IVF), that happened to occur during very early seroconversion. This was a USA patient whose *in vitro* fertilization had occurred 6 months prior to implantation of their embryo at a time that she was seronegative. In the interval between *in vitro* fertilization and implantation she had traveled to New Zealand where she likely acquired *T*.*gondii* infection in the weeks before implantation as shown in **Fig 3C**.

Our earlier studies (**Box 2 and Table 3**) have all identified perfect performance in detecting sera from those with acute infection in the USA. It was, therefore, of interest to determine whether the same would be found in sera from patients with acute infection with the genetically different parasites found in Colombia. **Fig 2E and Table 5 A-B** shows perfect ability, sensitivity, and specificity of the ICT to also identify acute infection (IgG, IgM) in Colombia (N = 22) and those who are seronegative (N = 12), (p<0.0001)(sensitivity 100% and specificity 100%).

**Table 5:** Results of the studies in Colombia with LDbio, Vidas G and M, and AdBio.

A. Contingency analysis of the results between positive IgG (A´) and IgM (A”) and seronegative samples in Colombian patients measured in Vidas and LDBio results in these patients. A’			
	**LDBio IgG/IgM qualitative (Positive or Negative)**		
**VIDAS IgG Result**	**Positive**	**Negative**	**Total**
**Positive**	22	0	22
**Negative**	0	12	12
**TOTAL**	22	12	34
**A´´**			
	**LDBio IgG/IgM qualitative (Positive or Negative)**		
**VIDAS IgM Result**	**Positive**	**Negative**	**Total**
**Positive**	22	0	22
**Negative**	0	12	12
**TOTAL**	22	12	34
**B. Contingency analysis of results of positivity and negativity for anti-*Toxoplasma* IgG (B´) and IgM (B´´) between VIDAS and rapid test Adbio and summary of diagnostic properties found in this group of serum (B´´´) including negative predictive (NPV) and positive predictive (PPV) values.** **B’**			
	**VIDAS IgG result**		
**AdBio IgG Result**	**Positive**	**Negative**	**Total**
**Positive**	82	6	88
**Negative**	0	59	59
**TOTAL**	82	65	147
**B´´**			
	**VIDAS IgM result**		
**AdBio IgM Result**	**Positive**	**Negative**	**Total**
**Positive**	5	0	5
**Negative**	22	120	142
**TOTAL**	27	120	147
B”‘			
**POC Adbio** **Anti-*Toxoplasma***	**Sensitivity %** **(95% CI)**	**Specificity % (95% CI)**	**NPV % (95% CI)/** **PPV % (95% CI)**
**IgG**	100 (94.4–10)	90.8 (80.3–9.2)	100 (92.4–100)/ 93.2 (85.2–97.2)
**IgM**	18.5 (7–38.7)	100 (96.1–100)	84.5 (77.3–89.8)/ 100 (46.3–100)

This brought the total to 144, as above. Further, serologic status was correctly identified for additional NCCCTS participants tested with finger-stick whole blood and serum between March and December 2018 herein (N = 20 positive chronically infected persons [times after infection years were known to be greater than 17 years for all except 3 persons], and 5 negative). We found perfect correlation of testing of whole blood obtained by fingerstick and serum testing in the United States, herein, and almost perfect correlation in Morocco this ICT using whole blood accurately distinguishes seronegative and seropositive status as occurs in seroconversion. In study 6 in which we tested whole blood (that contained serum that originally had 38 UI/ml of IgG and 63.89 ratio for IgM according to Roche *Toxoplasma* kits, diluted 1:89 in whole blood from a seronegative donor) at the limits of detection in the “QI study”, there was high accuracy in distinguishing positive and negative samples (**Fig 2F**). There were N = 63 negative and 63 positive, making a total of 126 tests of samples performed. There was 100% accuracy both with the cassette and with photographs read by the tester and two additional readers. All readings were congruent and consistent. All were blinded for 9 testers with 3 testers in each of three settings (physician office, nurse health care setting, and laboratory conference room), and with testers differing professional backgrounds (3 nursing, 3 medicine in training, 3 licensed physicians in practice previously unskilled in use of such a test) (**Fig 2F and Box 3)**.

### In Study 9, use of ICT for Cincinnati epidemiology study between 2017 and 2019 demonstrates that ICT is an efficient way to perform such studies and that prevalence is low in Cincinnati

**Fig 5:**
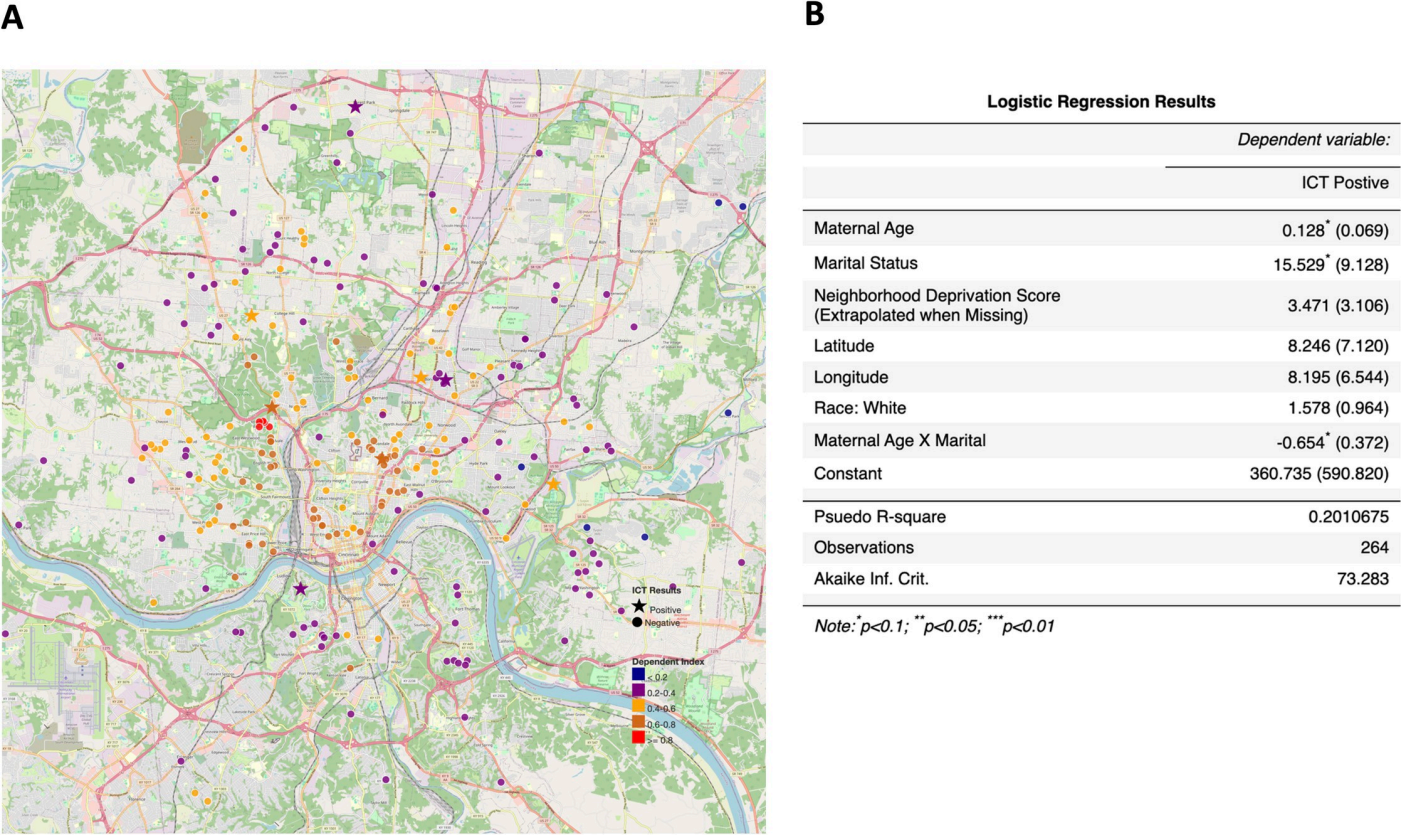
Location of seropositive persons in Cincinnati and associated demographic factors such as socioeconomic status, maximum educational level achieved, pet ownership, and ethnicity. Sera were collected between 2017 and 2019. The low prevalence of seropositivity did not allow testing for clustering by any known risk factors for infection, including proximity to watersheds associated with sewage water run-off. None of the sociodemographic parameters, neighborhood deprivation, nor residential latitude and longitude measures achieved statistical significance. The measures of neighborhood deprivation are indicated by the color of the symbols. **This map is generated from OpenStreetMap(https://www.openstreetmap.org/search?query=cincinnati#map=12/39.1506/-84.5007). OpenStreetMap data is available under the Open Database License, compatible with CC BY 4.0 license.**

Of the 265 mothers tested, 8 (3%) had a positive IgG for *Toxoplasma* infection. None of these had a positive IgM. Variables of interest were available for 264 of the mothers including residential address (longitude and latitude), age, education, race, income and pet ownership as part of the original cohort study. There were no significant associations of testing positive for *Toxoplasma* infection and any of these variables (**Fig 5**).

### In Study 10, results with ICT AdBio test (Onsite POC) that detects anti-*T*. *gondii* IgM and IgG separately has both substantial false negatives and false positive IgG (9%) and false negative IgM (18.5% true positives detected)

We had hoped that a test developed in the USA called ADBio that is purported to distinguish IgG and IgM specific for *Toxoplasma* might be useful in a field setting. This test had a high proportion of False negative and substantial number of False positive results (**Fig 2D and Table 5)**. A total of 147 samples were tested using the Onsite POC test (**Fig 2D** and **Table 5**) in Quindio, Colombia. By using *Toxoplasma*-IgG detection by VIDAS as reference test (**Fig 2D and Table 5**), the sensitivity of Adbio for *Toxoplasma*-IgG detection was 100% (95% confidence interval [CI], 94.4%–100%), and specificity for *Toxoplasma*-IgG detection was 90.8% (95% confidence interval [CI], 80.3%–96.2%). Of note, in 6 of 65 (9%) samples, the Onsite POC kit tested positive for IgG but were negative by VIDAS IgG. In 5 of these sera the bands observed in AdBio were visible. One serum with positive result for IgG in the AdBio but negative in the IgG VIDAS, was obtained from a boy with confirmed congenital toxoplasmosis who was being treated when the sample was obtained. For diagnostic accuracy of IgM detection, sensitivity for *Toxoplasma*-IgM detection was 18.5% (95% confidence interval [CI], 7.0%–38.7%; 5 of 27), and specificity for *Toxoplasma*-IgM detection was 100% (95% confidence interval [CI], 96.1%–100%; 120 of 120). Summary of the diagnostic properties found in this group of sera are shown in **Table 5**, including NPV and PPV values.

## Summary of results

**Fig 6:**
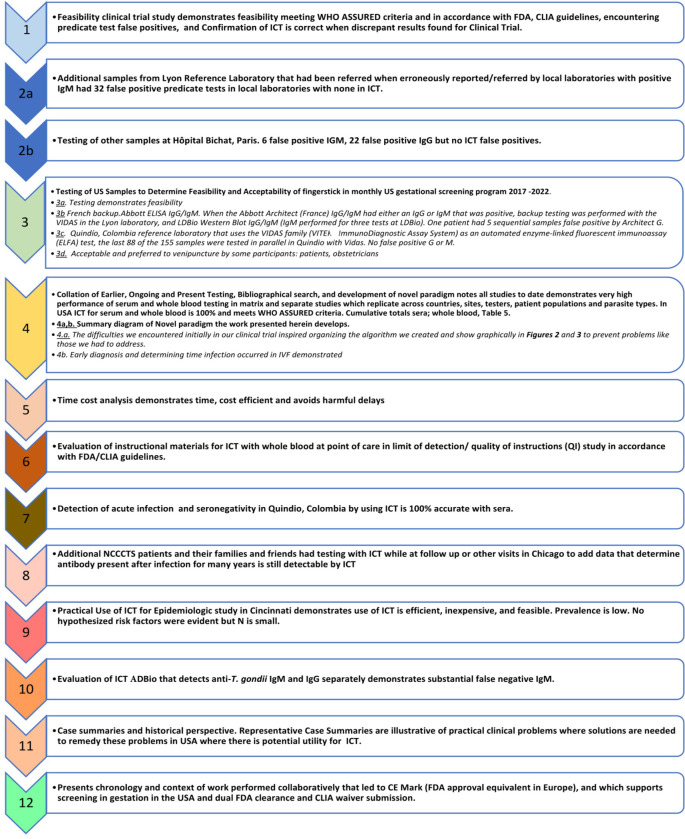
Summary of purpose and results of each study in a Roadmap of the Studies. Flow diagram of studies that provide roadmap to step changes leading to paradigm shift in prevention of congenital toxoplasmosis.

**Fig 6**. shows a Summary and flow chart of all Results showing Milestones achieved in this work. This shows the complexity of this decades long work that occurred in stepwise studies. **Fig 6** summarizes the results that corresponds with the Design of the “road map” in **Box 1**.

## Discussion (with S1 Commentary)

### Implications of main clinical findings In the 12 studies

#### Main clinical findings

The results above demonstrate that the ICT has proven effective at identifying sera and whole blood samples of USA and non-USA patients with known *T*. *gondii* infection with high performance for each circumstance/ patient/participant tested. The ICT recognizes both quadrivalent and pentavalent IgM and bivalent IgG bound by the antigens to the black beads and in a line at T on the chromatographic paper, functioning well for all persons in each site in north and Latin America, Europe and Africa. It detects seroconversion early in infection. It is also effective in identifying the false positive test results for *T*. *gondii* specific IgM of other currently FDA cleared tests of sera when no *T*. *gondii* specific IgG is present. It was well-accepted in a monthly screening program that was shown to be feasible in a USA academic obstetrical practice. It also functioned with high precision while meeting WHO REASSURED criteria even in whole blood samples at the limit of detection of specific anti-*Toxoplasma* antibody. It was found to be straightforward for physicians, nurses and medical students and a medical resident to easily learn to use the ICT and accurately interpret the ICT results using the Quick Information in simple written instructions.

Up through and including the current stages of the clinical feasibility trial at the University of Chicago Medical Center, diagnostic sensitivity has exceeded 99% and specificity has stayed at 100% with all samples of U.S. patients regardless of parasite or patient genetics. In addition, across several of these studies, this ICT has outperformed other NRL screening tests. Herein, out of 99 IgM false positive sample results, across multiple consecutive different USA and French sets of data recently, there have not been false positives or false negatives using the ICT. In addition, in two countries (the USA and Morocco [29]), the ICT has not had false positive or borderline bands when testing serum and/or whole blood in the USA and only very rarely in Morocco with whole blood. While it was already known that this test could perform accurately, this present work also has evaluated the ability of ICT to correct the errors of other carefully tested, commercially available screening assays [27], using prospectively and retrospectively collected sera in the USA, France and Morocco. The high specificity is a particular strength for the ICT IgG-IgM device, especially when compared to other currently available commercial tests for anti-*Toxoplasma* IgM.

The data from Abraham, Houze’ et al (ECMID and [30]) and Mahinc et al [26] increases the number up to 137 of such false positive IgM studied with the ICT. Mahinc et al also studied 23 false positive Architect and/or Bio-Rad Platelia IgM [26]. In the Mahinc study [26], false positive IgM in the Bio-Rad test were obviated by ICT testing 21/23 of the time. In Tunisia [31], recent results were similar adding additional data but with a higher proportion of false positives [31]. Ten of 13 false positives were negative in the ICT. Although there were no ICT false positives in these data sets in the US, the occasional false positives (5 of 36) in the work earlier in Marseilles and Tunisia emphasize the importance of confirmatory testing of positive results. The high-quality performance of some of the Reference tests emphasize that some tests seem to perform better than others, with greater or lesser sensitivity for IgM, when used in Reference laboratories.

Our studies, along with the earlier experience in the Palo Alto reference laboratory and collated recent results, demonstrate practical problems in the US with potential serious consequences for patient care using NRL tests [35] where the ICT can be helpful in a patient’s management. This has been confirmed in France making a total of 132 of 137 IgM and 27 of 27 IgG false positive results were corrected. False negatives are uncommon but would be detected by repeat testing in gestational screening programs. Any positive ICT during gestation would have confirmatory testing to differentiate IgG and IgM. The occasional false positives would be detected by back up testing in the reference laboratory in the USA or use of multiple tests including the Western blot in France. Reference laboratory gold-standard testing and certain commercially available test reagents have higher performance than testing in local laboratories [27,30]. The ICT has high precision with samples at the limit of detection. That the test is easy for medical students, a medical resident, practicing board certified physicians, nurses/nurse practitioners, without familiarity with the ICT, to perform and interpret is congruent with a recent experience with 30 practitioners in Armenia, Colombia [47]. This experience was with patients infected with genetically distinctive Colombian parasites [47]. Acceptability in a Colombian patient and obstetrical practitioner group was high [47], similar to acceptability in our USA experience presented herein.

Colombian sera also were tested in Colombia [47–50] with a different lateral chromatography test made in the USA called the ADBio. This test differentiates IgG and IgM and has a USA price more than ten times that expected for the ICT. Unfortunately, the performance of the USA manufactured ADBio test was problematic when compared in the Quindio Reference laboratory with Vidas IgG and IgM reference tests [47–50]. This is similar to our earlier results with this test with French, and USA [27], sera. For the Colombian sera specifically, there was a marked difference of the ICT and combined detection of IgG and IgM antibodies: The AdBio test resulted in lower sensitivity for IgM in stored samples from a biobank. ICT combined simultaneous detection of IgG and IgM can improve sensitivity for IgM because most of the IgM sera used for sensitivity analysis already have IgG [26,27,30} and the mechanism of the test with antigen coating the black bead reacting with both quadra/pentavalent IgM and bivalent IgG which react with the antigen placed in the line on the nitrocellulose. This combined detection of different isotypes also contributes to better specificity. The lysate antigen used in the ICT contains many proteins. The Western blot can accurately discriminate between and recognize IgG and IgM specific for *T*. *gondii*, as can the combination of other tests such as the Sabin Feldman Dye test which detects IgG and the double sandwich IgM ELISA or the IgM ISAGA. The IgM ISAGA is more sensitive and thus preferable for use for infants.

### Interpretation in view of the literature, and additional strengths and limitations

#### Interpretation

In the context of clinical protocols for prenatal *Toxoplasma* screening, the ICT insures that far fewer “false alarms” are generated and that less time and resources are spent on confirmatory testing for a pregnant woman who shows an isolated positive *Toxoplasma* IgM test. Risk that such sample may be a false negative IgM from the ICT test is very low, but cannot be excluded. To avoid any risk the patient should be retested for IgG and IgM 2 weeks later to ensure that IgG did not appear. This re-testing for isolated IgM, at this shorter interval, is also part of systematic gestational screening programs. It should be emphasized again that POC tests for anti-*Toxoplasma* IgG and IgM, such as the ICT, are merely a first step toward diagnosis, given that IgM antibodies can persist for up to several years after acute infection. For any woman who receives a false positive IgM test result, the next step of an evaluation with other tests can involve weeks of waiting for a blood sample to be tested using technology that runs at much higher costs than the point of care test [1,2,6,7,27,33,35]. Persistence of IgM in subacute or chronic infection, results just above the cut off or more, and true seroconversion all cause positive IgM results. This test does not distinguish persistent IgM and that present with true seroconversion. Ideally, testing is performed initially pre-conception. If testing in gestational screening programs was not performed pre-conception to identify serologically positive persons who would not require monthly screening to detect seroconversion, testing in gestational screening programs should take place no later than during the initial 14 to 16 weeks of amenorrhea, and preferably earlier at the first pre-natal visit. This enables use of the avidity test to attempt to date infection when IgM is detected. As mentioned above, IgM can be present from an old infection or early in seroconversion. A high avidity test result can indicate infection older than 14 to 16 weeks. A low avidity can occur in infection that is recent, within 14 to 16 weeks but also can remain positive for extended times (even years). So, with a positive IgM, an avidity test may be helpful, and should be utilized, to establish timing of infection. Extremely rarely, a woman infected before conception has transmitted infection to her fetus with reports of this being associated with an unusual strain of *Toxoplasma* that is different from the initial infection. There is not presently a serologic test that can identify this rare occurrence.

A hook study using the Abbott Architect by Mahinc et al [26] showed that the ICT performed well across a range of IU, with samples from <1 IU to much higher levels, detected IgM alone, IgG alone and combined IgG and IgM. The study of Chapey et al [25] showed a measured AUC close to 1, using quantitation with scanning. The ICT performed better than or as well as every predicate FDA cleared test using the FDA, CLIA cleared Remington Specialty *Toxoplasma* Serology PAMF Reference laboratory tests with the CDC set of approved samples.

#### Summary of additional strengths

Strengths include: (1) evaluation of a screening test for *Toxoplasma* infection that detects G, M or G plus M antibodies with high precision; (2) that meets WHO REASSURED criteria; and (3) creates a new algorithm for screening for *Toxoplasma* infection. This test identifies infection using whole blood or serum with NPV 100%, PPV 100%, AUC 1 in all studies in the US with thousands of sera and whole blood tests by multiple testers and participants in varied settings. Results with an earlier pink line (red bead) then black bead did not differ for serum samples. This work demonstrates that previously published pink line kit testing of sera and currently used black line kit testing of sera are comparable. This was documented with 1074 sera from 4 sites in the south of France, with similar findings of comparability in Chicago studies. This black bead kit then was used for testing whole blood. The sensitivity and specificity did not differ between serum and whole blood samples in the Chicago, USA studies. It lowers costs and improves acceptability of testing. Serial studies were designed and carried out to address regulations in a manner that meets FDA and CLIA criteria for clearance and waiver in US and CE mark in Europe. This is so this test can be used easily with confidence in its efficacy, safety and reliability.

This test creates a novel screening model for testing pre-pregnant and pregnant persons, for rapid confirmatory testing in a clinical lab. Adverse consequences of infection can progress rapidly *in utero* making prompt diagnosis to enable treatment of value [3]. As discussed below, this test is also useful for those at risk for or who have retinal disease, lymphadenopathy, in epidemic settings, for public health assessment, for testing medicines or vaccines that are curative or prevent infection and disease, and other clinical circumstances enabling prompt diagnosis and treatment, as also discussed under “Implications for Public Health” below.

#### Limitations

Limitations include the following: This test does not distinguish IgG and IgM, whether IgM is part of new, acute acquisition, residual, persistent IgM from earlier infection pre-pregnancy or late in gestation, but can distinguish a false positive result from a commercial test that is slightly or more above the cut off value in the commercial predicate tests. In low prevalence areas false positives can confound care more frequently. The performance in the US and Paris is so strong that it is not possible to present a proportion of positives with 1 primary infection per 1000 pregnancies. Differences in the percentages of sero-positives, reflects differing participant populations, with some whole blood tests for large numbers of participants occurring in high prevalence settings of Morocco and Colombia, and in some US studies in US enriched for seropositive persons. The Chicago/Paris and Cincinnati studies with very low prevalence with larger numbers of sera also influence those proportions. The sensitivity and specificity of the serum and blood testing did not differ significantly in any study with simultaneous testing in the USA in multiple studies. Test methodology names to obtain links on Google are in the Box 2 legend.

Experience in Tunisia is cautionary, and it may be more problematic as there were more false positive results. In the future there might be more variability in different field settings. Positive results that influence patient care should be confirmed in Reference laboratory or with Western Blot for this reason. Thirty three of 39 French Reference laboratories use the Western blot as the gold standard confirmatory tests but this is not available currently in the USA. The ICT is intended to involve medical personnel and should include those who are skilled in interpretation of serologic results and patient care for those with *Toxoplasma* infection and toxoplasmosis. Even a technically perfect test for these diseases requires clinically knowledgeable users to properly provide informed medical care for an infection and illnesses with serious consequences. Proper care can be life, sight, cognition and motor function saving lifelong. This is not a test for lay users for these reasons.

Testing before conception, to identify seropositive persons, and then testing regularly monthly through pregnancy for those who are IgG seronegative initially, would be ideal as it helps to obviate problems of persistent IgM, testing at the initial visit and often later times in gestation, anxiety provoking delays that can result in irreversible fetal damage, as well as false positive test results. Such damage in congenital toxoplasmosis, as well as in ocular toxoplasmosis can occur in very short times of less than a week, making diagnosis and initiation of treatment urgent and emergent. With clear seroconversion, treatment can be initiated presumptively while waiting for reference laboratory results. Positive results should always be confirmed. Minimizing the likelihood of false positive IgM while maintaining maximum sensitivity is a top priority for any point of care test candidate.

### Implications for clinical laboratory practice

The ICT also should be very useful in clinical laboratories testing with sera with a potential false positive IgM result without IgG as described herein. It could function as a second-line test to confirm or find IgM specific for *T*. *gondii* is not present before sending the sample to a reference center, while continuing to follow the patient while awaiting Reference laboratory results. This is a major advance as this will save time and reduce the need for gold standard tests. It can help reduce concern for patients and physicians. When the ICT test is used initially with whole blood the only predicate test for confirmation needed will be if the whole blood test is positive. ICT not only helped to obviate the problems with false positives but also can result in detection of true positives and very early seroconversion as described herein and also recently acquired infections described elsewhere [9,23,24,26,27,30].

### Implications for Public Health for Large Scale Gestational Screening Programs to reduce burden of congenital toxoplasmosis, to reduce the burden of eye disease from post-natal infections, and in a variety of other settings

We placed this work in the context of ongoing problems for healthcare (**Figs A and B in S1 Commentary**) and potential for direct and spillover benefit for the care of pregnant women and their families (**Table A in S1 Commentary**, [14]). We also placed these studies 1 to 12 herein in a historical context building parts of a toolbox working toward a role of screening using WHO REASSURED criteria compatible tests in the elimination of congenital toxoplasmosis (**Figs A and B in S1 Commentary)**.

As considered above for individual clinical circumstances and in the local clinical laboratory there are similar additional public health considerations in the same categories. There also are a variety of other clinical and epidemiologic circumstances where knowing *T*. *gondii* serologic antibody status can be of considerable clinical and public health utility and benefit [1,2,6,7,27,32–9,46–50]. Very high-quality, low-cost screening tests such as the ICT can improve infectious diseases care in gestation, eliminating perinatal infections. There are a variety of public health threats to pregnant women from wildtype *Toxoplasma* as well as parasites that can be infected with single stranded RNA viruses in fresh and sea water, venison, worsening with heavy rainfall, endemic and epidemic. Value extends beyond care for toxoplasmosis to other aspects of health care for pregnant women and other clinical and research settings. Large scale screening programs reduce the burden of congenital toxoplasmosis for individual suffering and societal costs.

### Implications and perspectives for further research

Congenital toxoplasmosis is a treatable and preventable disease, and physicians and other obstetrical providers now have the tools, in-hand, to improve outcomes and reduce patient and familial suffering. This screening, the standard of care in other countries, is now increasingly feasible in countries like the United States, where the primary argument against screening has been its economic burden. In the development of this test and other high-functioning point-of-care tests, there is potential for transformation in the provision of obstetrical care to improve maternal-child health. These benefits are amplified in subpopulation demographics in the USA [28] and regions of the world where the burden of disease is even higher. Examples of this occur in the Lancaster Amish population in the USA [8,12,32,46], parts of Florida, are likely in other US subpopulations [28], and occur in Central [36] and South America [47], and parts of Africa [29].

Use of the ICT for the Cincinnati maternal cohort study found ICT to be efficient (Study 9). Due to the small proportion who were seropositive, we were unable to test for any clustering by known risk factors for exposure: none of the individual socio-economic or location factors in a regression analysis achieved statistical significance. A larger overall sample size will be needed to evaluate risk factors in this population. Reasons for relatively low prevalence in Cincinnati in this cohort remain to be discovered. Even with the low prevalence found, it is likely still that gestational screening would be worthwhile.

Our recent study in Colombia also demonstrated high acceptability of a single use of the POC on a large scale of 783 women and 30 providers [47]. Although *Toxoplasma* infections occur in all demographics it was a particular problem in those who had lower education and socioeconomic status [31,47]. To understand risk factors during gestation and to develop programs to prevent such infection will require monthly screening in areas of high to low prevalence.

The implementation of this study in the clinical trial and the QI limit of detection study demonstrated that it should be easy to introduce this test into obstetrical or other practice taking little extra time or causing inconvenience. For example, when patients are evaluated for vital signs, blood pressure, glucose including by fingerstick, by a medical assistant or nurse, the test can be performed and the cassette can also be brought to the obstetrician or other health care practitioner for additional reading and entry into the medical record. Photography, using a smart phone for documentation, could easily be included for when the care provider requires assistance with interpretation and for additional documentation for the patient and incorporation into the medical record.

In France screening was mandated by law. In Austria those screened received additional health care benefits. In Colombia it was introduced through practice societies. In the USA those in advisory positions recommended that education, easy feasibility, low cost would result in those who would benefit choosing to have testing incorporated in medical practice and USA patient culture at many levels by personal preference. The acceptability study demonstrated that informed patients would want this and obstetricians could use this comfortably and without inconvenience in their practice. It could easily be introduced into family practice and adolescent pediatric care to identify seropositive patients at risk of this most common retina disease due to infection and loss of sight. Such screening in adolescence could also provide pre-pregnancy testing for young women to allow knowledge of who is seronegative and should be screened during pregnancy. Pre-marital/conception screening, as initially occurred in France, could also be helpful as families plan to have children. As *Toxoplasma* has been transmitted by organ donation and white blood cell transfusion and by sperm in domestic non-human animals, can relapse with immune suppression, and may be causative for epilepsy and some neurodegeneration, there are a number of other medical settings where knowledge of *Toxoplasma* serologic status may be useful.

A future possibility to obviate the potential limitation of use of saliva which does not perform well with ICT includes a test that might be used with saliva or serum or whole blood in conjunction with the ICT if developed further in the future. This nanogold NIRMIDAS test was studied with saliva, serum, and whole blood. It was found to have high sensitivity and specificity and dye test precision for the detection of IgG, and for IgM [37]. We had suggested earlier this might be an ideal test to use before conception or if cost were constrained and it was feasible for initial testing in gestation. Although finger stick for glucose is standard, easy, and familiar in obstetrical practices, obtaining saliva may be viewed as less difficult than whole blood. Thus, some might view testing using saliva a potential advantage. However, the nanogold for any sample, so far, would require transport to where the machine is, associated delays to reach a clinical laboratory, and electricity and a sophisticated machine for testing. Recently manufacture of this nanogold test was discontinued. NIRMIDAS has also used a gold bead ICT for SARS CoV-2, but nothing like this has been produced for *Toxoplasma* to date. The diagnosis and management of *Toxoplasma* infection requires a knowledgeable health care provider urgently making home testing of saliva less advantageous.

Another consideration for the future includes the evaluation of the potential for cost savings to individuals, health care providers and systems as well as limiting suffering for individual patients and their families. As a foundation for such analyses in a variety of settings, to understand if the ICT should be widely used in gestational screening programs, studies of cost efficacy were performed [14]. Initially, we, with economists, created a mathematical model which can be used for such analyses in various settings. We first demonstrated potential cost benefit using health care data in the US [14], which found a financial cost saving incentive for screening programs sponsored by government health care systems as took place in France. With this first adaptable mathematical model [14], we applied it to actual costs in Austria [15] and then in France [16,17] with economists Drs. Stillwaggon and Sawers. Cost/benefit was shown elegantly by Stillwaggon, Sawers, Prusa, and Kasper to occur even with low prevalence as in Austria. Gestational screening was found to be 14- fold cost saving for the health care system, as well as being good for people, saving life, sight, cognition and motor function in these countries [16,17]. The important study of Mandelbrot et al [3] demonstrated the benefit for improved gestation outcomes from prompt diagnosis and treatment, as a major consideration for a screening program. A well-functioning point of care test meeting WHO REASSURED criteria, adds time and cost savings as shown by our analyses herein. In the future this could be an additional cost savings and benefit consideration for gestational screening and treatment as well as in a variety of health care systems and in epidemiologic study.

In future studies, and perhaps ultimately in the future in clinical practice, the ICT also might be useful to demonstrate efficacy of candidate anti-parasitic agents [51]. This could be useful in establishing definitive cure of infection. This would be by demonstrating elimination of presence of any serum antibody to *T*. *gondii* with elimination of the three clinical forms of *T*. *gondii* (tachyzoites, bradyzoites in cysts, and “persister” organisms). This might be used to establish definitive cure in those treated in clinical trials, and with the possible goal of ICT use, ultimately, in clinical practice in this manner. It might be useful to establish vaccine efficacy [52]. The work herein provides part of the foundation for such studies. The ICT has also been utilized in studies of seroprevalence in wild-life and in characterizing epidemics taking place in zoos in primates and marsupials [53,54]. These latter studies are being performed in conjunction with studies of a new vaccine for zoo animals. In the future the ICT could be an efficient, low cost tool for one health and other epidemiologic studies of prevalence, and in epidemic as well as endemic settings.

### Implications of additional considerations, and perspectives, concerning this novel test, paradigm, implementation, and practical use of this approach

The following information addresses some of these special considerations: For context, we emphasize that our initial specific overall objectives of this study were to determine the performance of the ICT which we found to be sensitive and specific as described herein. When we found high performance in initial studies, our objectives were to carry out a series of well-controlled studies to assess whether the test met WHO REASSURED criteria for a point of care test, could meet all FDA and CLIA testing requirements to enable its use in the United States and elsewhere, and to determine feasibility of implementation and acceptability of use of the ICT in multiple real-life clinical settings and in an epidemiologic study. These objectives were achieved successfully as outlined above. Nonetheless, even such a high performing test was not developed and does not function well in a vacuum. Rather, this is part of a tool box beginning with knowledge of the diseases *Toxoplasma* causes and their consequences, and thereby whether, how, when and where to use the test. Certain special considerations concerning ease of use, learning to use the test, who could use it successfully, feasibility, ease of implementation, reproducibility, repeatability, and acceptability became evident in our studies. Herein, in all our studies in Chicago, per IRB regulation, 100% of our results were confirmed, and in practical use any positive result was also confirmed. Reference laboratory back up must be performed for any positive ICT result during gestation, especially seroconversion. We do not advocate home testing by patients, but believe testing should be performed with availability of a well-educated, knowledgeable physicians’/care givers’ advice because of the substantial impact of this congenital infection on outcomes lifelong.

The FDA/CLIA regulations required multiple settings and multiple testers in these separate stepwise studies. The variety of settings for point of care finger prick for whole blood, as shown in the individual patient/participant tables varied and the results were robust in all. Among all our US studies herein, testers included those with a wide variety of relevant backgrounds and a wide variety of relevant settings listed in Box 2 legend. All US tests were read by the testers and additional medical and laboratory personnel, and readings were documented by photograph. All readings in the US were congruent among multiple readers who worked independently and who did not influence each-others’ readings of the test kit or photographs of the test kits. Readings were consistently congruent by varied testers in different settings. In the US, every person who had a whole blood reading had serum results. The earlier pink line serum test was found to be congruent with the black line whole blood and serum tests herein. Photographic documentation was performed with all US study readings in Chicago, and at Stanford earlier, with multiple congruent readings both for whole blood and for serum in Chicago. As we did in these studies, we suggest that the results influencing clinical care, for example seroconversion or a positive test during gestation, be confirmed in a reference laboratory, and also be documented by smart phone or other photograph.

Concerning education, the test kit brochure has a 24/7 telephone number where photos can be reviewed for assistance. We have created a You tube movie linked to reference [23], and an instructional test set of photos we will place on our website. We have created a suite of educational materials for various audiences (high schoolers, when considering pregnancy, during pregnancy, medical students, residents, fellows, practicing physicians, in text book chapters) [6,7]. Educational materials have improved knowledge in all those settings [32–5]. Concerning educational materials, families of affected children (some of whom are now adults) want to introduce information at multiple levels. This was to try to build a robust system of informed persons and call systems when physicians, other care givers, or patients need advice. In the US, in June 2023, eight states (Hawaii, Arkansas, Nebraska, Kansas, Kentucky, Pennsylvania, Minnesota, Wisconsin and Delaware) have made it the law to report toxoplasmosis. They are creating a knowledgeable cohort of CDC epidemiologists in those state programs. They have created uniform reporting criteria which were approved by the full Council of CDC epidemiologists nationally and in all 50 states and territories in June 2023. To help to enable screening in those settings as noted above, we have made educational materials freely available [32–5]. We view the ICT as part of a “toolbox” to implement high quality care to prevent toxoplasmosis, where education and expertise in interpretation and advice are critical. **Tables 1, 2**, **3A and 3B and Box 2** and **Tables A, B and C in S1 Commentary** also illustrate feasibility, and successful implementation of testing. Although ICT result informs initial approach to care, a positive result should be confirmed and in certain clinical circumstances such as during gestation with differentiation of acute from chronic infection is essential. Exceptions for need for confirmation include epidemiologic prevalence studies where the test is not influencing direct patient care.

The high performance of the ICT and development of this new paradigm for care (as shown in **Fig 3** and **Table 6**) can help to improve outcomes for congenital toxoplasmosis substantially and contribute to its elimination. As noted above, this neglected disease causes a significant health burden with 190,000 infants or more infected each year causing 1.2 million disability adjusted life years. Obstetricians, nurse midwives, family practitioners, obstetrical nurses, and other obstetrical providers, neonatologists, pediatricians, infectious diseases specialists, policy makers, public health officials, and others are uniquely positioned to intervene to prevent this disease and improve the health of both mother and child. POC test based monthly gestational screening of seronegative patients for *T*. *gondii* infection provides a valuable tool in the obstetric armamentarium to ensure maternal-child wellness and lower its health-care burden around the world. Inclusion of POC-ICT into *Toxoplasma* diagnosis brings considerable promise.

**Table 6:** Summary of Role of ICT and Novel Paradigm in Approach to Screening and Actions Taken.

Reason for screening	Time of ICT	Back up Test	Enables diagnosis for Treatment
Pre-pregnancy Screen to establish serologic status	Adolescence Considering pregnancy	IgG IgM if positive ICT	Know serology Eye exam if positive Pre-pregnancy
To detect retina Infection, other	Symptomatic Late Adolescence.	IgG IgG	Rx Based on clinical findings. Pre-pregnancy Know serology, Eye exam if positive
Pregnancy	Before 14–16 Weeks Amenorrhea and Monthly if negative	Positive ICT or seroconversion confirmed = > IgG, IgM local laboratory,^a^ ICT can be used to consider whether isolated IgM is a false positive result with whole blood fingerstick sample and in clinical lab if ICT is negative ^b^ = >Reference M positive = >Avidity, AC/HS Consider amniocentesis; Management is based on clinical status	Diagnosis, seroconversion or if confirmed acute = > Treat pregnant woman to block transmission to fetus or treat fetus

^a^ The use of predicate tests in NRLs are not gold standard testing in reference laboratories.

^b^ Seroconversion documented with ICT aids in estimating timing of maternal infection. This point-of-care finger-prick test can be used for first-line screening.

Confirmatory tests are needed to determine whether IgM positive tests correspond to seroconversion or are non-specific (“false positives”) NRLs.

All negatives would have monthly follow-up, while all positives would require IgM testing and if positive, would require avidity testing and analysis of sequential sera to estimate the timing of maternal infection. The ICT complements and does not replace gold standard testing, and that finding seroconversion at the point of care with a highly accurate test can expedite initiation of treatment while confirmatory testing takes place.

**This new paradigm is to have a test that meets WHO REASSURED standards available promptly at the time the test is performed and to have a first backup of positive results in serum rapidly in the local laboratory in the context of knowledgeable medical care and reference laboratory backup testing. Table 6** contains a summary of approaches to testing in screening programs and some of the ways in which results for this novel paradigm are actionable for care providers. This includes and is especially focused for the pregnant woman and her fetus. Those who are seronegative are tested monthly until 6 weeks post-partum. Pre-conception testing is obtained when possible. Testing for IgG and IgM is obtained for those who are seropositive, and for anyone who has IgM an avidity and AC/HS test is potentially helpful to date infection. The ICT is also potentially useful in a variety of other clinical settings. When such tests have undergone appropriate evaluation by the FDA and CLIA, as they have undergone in CE-mark evaluation and approval in Europe, this testing can enable substantial improvements in management of risks associated with exposure to *T*. *gondii*.

## Conclusions

This ICT meets WHO REASSURED criteria, with extremely high performance in testing serum and/or whole blood at the point of care to rapidly provide evidence about whether a person is infected or not. The studies herein also demonstrate effective initial use of the ICT for rapid backup of positive results in the local laboratory thereby providing considerable benefit in concert with knowledgeable medical care and reference laboratory backup testing.

## Supporting information

S1 Commentary **Box A**. Case vignettes provide representative practical examples from false negative and false positive *Toxoplasma gondii* IgM tests in the USA that harm patients and patient care. **Table A.** Study 1. Design and Data for 3 Testers with 5 Sera-Positive Persons for each tester. Each in Three Settings with Results Showing Their Primary Data in the Chicago Clinical Feasibility Implementation Trial 2020 to 2021. Corresponds to Fig 2A. This Study is Performed in Accordance with FDA and CLIA Guidelines and Regulations. **Table B.** Study 2 Part 1 Lyon Reference Laboratory ICT Test Results for Sera from Pregnant Patients Referred by Local Physicians for *T*.*gondii* IgM with Predicate Tests in Local Laboratories and Negative Western Blot as Gold Standard Comparator. The organization and data correspond to Fig 2B. **Table C.** Study 3 Shows Concordance of ICT Results in Chicago Acceptability of Monthly Testing In Testing of Sera in Lyon Reference Laboratory Using Abbott Architect and for one person VIDAS IgG ELISA, and VIDAS G and M ELFA in Quindío Reference Laboratory. Initial tests in earlier months were all concordant with Abbott Architect and reported in Lykins et al [28]. This study corresponds to Fig 4 which shows the results of USA Acceptability, Study 3. **Fig A.** Overview Summary of studies herein in context of other work. Top presents context of current studies toward introducing screening in a global initiative. Red font shows work herein. Fig S2 summarizes the studies herein. **Fig B.** Historical perspectives on screening and treatment of *Toxoplasma gondii* acquired in gestation in France and the USA. This figure is to provide context of where we have come from to studies herein with considerable spillover benefit for patient care and well-being, with the goal that studies herein will provide a foundation for improvement of prevention and care for congenital toxoplasmosis.
(PDF)

S1 Text Disclosures and insuring Objectivity in Results. (DOCX)
