# Supplementary material for: Novel paradigm enables accurate monthly gestational screening to prevent congenital toxoplasmosis and more
Source: PLoS Negl Trop Dis. 2024 May 28;18(5):e0011335. doi: 10.1371/journal.pntd.0011335 (PMC11132520; doi:10.1371/journal.pntd.0011335)
Supplement: S1 Commentary — Box A. Case vignettes provide representative practical examples from false negative and false positive Toxoplasma gondii IgM tests in the USA that harm patients and patient care. Table A. Study 1. Design and Data for 3 Testers with 5 Sera-Positive Persons for each tester. Each in Three Settings with Results Showing Their Primary Data in the Chicago Clinical Feasibility Implementation Trial 2020 to 2021. Corresponds to Fig 2A. This Study is Performed in Accordance with FDA and CLIA Guidelines and Regulations. Table B. Study 2 Part 1 Lyon Reference Laboratory ICT Test Results for Sera from Pregnant Patients Referred by Local Physicians for T.gondii IgM with Predicate Tests in Local Laboratories and Negative Western Blot as Gold Standard Comparator. The organization and data correspond to Fig 2B. Table C. Study 3 Shows Concordance of ICT Results in Chicago Acceptability of Monthly Testing In Testing of Sera in Lyon Reference Laboratory Using Abbott Architect and for one person VIDAS IgG ELISA, and VIDAS G and M ELFA in Quindío Reference Laboratory. Initial tests in earlier months were all concordant with Abbott Architect and reported in Lykins et al [28]. This study corresponds to Fig 4 which shows the results of USA Acceptability, Study 3. Fig A. Overview Summary of studies herein in context of other work. Top presents context of current studies toward introducing screening in a global initiative. Red font shows work herein. Fig S2 summarizes the studies herein. Fig B. Historical perspectives on screening and treatment of Toxoplasma gondii acquired in gestation in France and the USA. This figure is to provide context of where we have come from to studies herein with considerable spillover benefit for patient care and well-being, with the goal that studies herein will provide a foundation for improvement of prevention and care for congenital toxoplasmosis. (PDF) [file pntd.0011335.s001.pdf]

S1: Commentary

Where have we been, where are we now, and where might we be in the future?

S1 Supporting information for: Novel paradigm enables accurate monthly gestational screening to prevent congenital toxoplasmosis and more

Running Title: Novel paradigm to prevent congenital toxoplasmosis and more

Zhou Y[1,]¶, Leahy K[2,]¶, Grose A[1, 3,,]¶,#, Lykins J[1,3,]¶, #, Siddiqui M[2], Leong N[2]. Goodall P[2], Withers S[1, 6], Ashi K[1,3,], Schrantz S[3,4,5,,6], #, Tesic V[3],[6, 7], Abeleda A P[6, 7], Beavis K[3, 6, 7], Clouser F[1], Ismail M[2, 3, 6], Christmas M[2, 3, 6], Piarroux R[8,]#, Limonne D[8], Chapey E[9], Abraham S[10], Baird I [11, 12], Thibodeau J[11,12], Boyer K[13], Torres E[14], Conrey S[15], Wang,K[16], Staat MA[15], Back N [157], L'Ollivier C[817, 18], Mahinc C[19], Flori P[19], Gomez Marin J[14], Peyron F[9], Houze S[10], Wallon M[9]*, McLeod R[1, 3, 5, 6, 11,12,]*

[1] Department of Ophthalmology and Visual Science, The University of Chicago, Chicago, Illinois,The United States of America
[2] Department of Obstetrics and Gynecology, The University of Chicago, Chicago, Illinois, The United States of America
[3] Pritzker School of Medicine, Division of Infectious Diseases, The University of Chicago, Chicago, Illinois, The United States of America
[4] Department of Medicine, The University of Chicago, Chicago, Illinois, The United States of America
[5] Department of Pediatrics, Division of Infectious Diseases, The University of Chicago, Chicago, Illinois The United States of America
[6]Chicago Medicine, The University of Chicago, Chicago, Illinois, The United States of America
[7] Department of Pathology, The University of Chicago, Chicago, Illinois, The United States of America
[8] LDBIO Diagnostics, Lyon, France
[9] Institut des agents infectieux, Hôpital de la Croix-Rousse, Lyon, France
[10]Laboratory of Parasitologie, Bichat-Claude Bernard Hôpital, Paris, France
[11] The College, The University of Chicago, Chicago, Illinois, The United States of America
[12] Global Health Center, The University of Chicago, Chicago, Illinois, The United States of America
[13]Department of Pediatrics, Division of Infectious Diseases, Rush Presbyterian Hospital and Medical Center, Chicago, Illinois, The United States of America
[14]Group of Molecular Parasitology (GEPAMOL) , Center of Biomedical Research, Faculty of Health Sciences, University of Quindio, Armenia (Quindio), Colombia
[15]University of Cincinnati and Cincinnati Children's Hospital Medical Center,The University of Cincinnati, , Cincinnati, Ohio, The United States of America
[16]Carl H. Lindner College of Business, The University of Cincinnati, , Cincinnati, Ohio, The United States of America
[17] Centre National de Référence Toxoplasmose - Pôle Sérologie, Hôpitaux Universitaires de Strasbourg, Strasbourg, France
[18] IHU-Méditerranée Infection, Assistance Publique Hôpitaux de Marseille (AP-HM), Marseille, France; Aix Marseille University, IRD, AP-HM, SSA, VITROME, IHU Méditerranée, Marseille, France
[19] Parasitology and Mycology Laboratory, Pôle de Biologie-Pathologie, University Hospital of Saint Etienne, Saint Etienne, France

#Current addresses: A. Grose, Departments of Medicine and Pediatrics, Duke University, Durham NorthCarolina, The United States of America ; J D.Lykins, Department of Emergency Medicine at Boston Medical Center and Boston University School of Medicine, Boston, Massachusetts, The United States of America; S.Schrantz, ICON, Evanston, Illinois, The united States R. Piarroux, Efor CVO, Champagne Aux Monts d'Or, France

*Corresponding authors: rmcleod@bsd.uchicago.edu; martine.wallon@chu-lyon.fr
¶Equal contribution

This **Commentary** addresses where approaches to prevention and treatment of congenital toxoplasmosis have been as the studies herein were developed and carried out and our goal that the studies herein provide a foundation to move beyond the current status of prevention and care in the US[1-54]. We provide tables and figures to summarize and to provide historical context. We present cases representative of those that we encounter regularly (**Box A**).

Box A. Case vignettes provide representative practical examples from false negative and false positive *Toxoplasma gondii* IgM tests in the USA that harm patients and patient care

1. **In 2022 problems persist for false negative results with commercially available ICT.This resulted in missed diagnoses with 2/3 false negative *Toxoplasma* IgM with ADBio** (Gomez C et al, 2017; Gomez Marin et al herein) *These are available commercially but can result in mis-diagnosis. Not FDA cleared or CLIA waived, accurate, sensitive, specific tests.*
2. **Loss of life, sight, cognition, motor function, seizures secondary to missed diagnoses continues in 2022** (Soberon-Felin et al, Part I and unpublished observation in 2022)
   A fetus with hydrocephalus was identified at 25 weeks gestation. The baby was born at 35 weeks gestation, at which time severe involvement from Toxoplasma infection involving the brain and eyes was identified. No screening for Toxoplasma had been performed for the mother.
3. **Lack of trust in positive result by obstetrician when patient had positive IgM, result ignored because of distrust. Infant born with symptomatic congenital toxoplasmosis in 2022.**
   An obstetrician ordered serologic tests for a pregnant woman. He found a positive IgG and IgM in the first trimester. He knew that there were many false positive test results and thus ignored the result. An infected infant was born 6 months later.
4. **Similar to the above case, an obstetrician did not trust a positive IgM, an infected infant was born, and 16 years later, the teenager faced significant problems**
   An obstetrician found positive IgM and did not trust the test. An infant was born with congenital toxoplasmosis, and at age 16, the teenager is facing a severe seizure disorder and loss of sight.
5. **False positive IgM very alarming to patient; earlier, pregnancies were terminated in the decades before 2022 iteratively.**
   This resulted in FDA guidance that sera with positive results be referred to reference laboratories (37).
6. **In 2022, false positive IgM very alarming to patient, causing considerable anxiety and confusion and inconvenience. Sera ultimately sent to reference laboratory with long delays. Patient was seronegative. In 2022.**
   A pregnant woman at 20weeks gestation was gardening without gloves and noticed cats in her backyard. She contacted her doctor, who ordered a standard predicate test for toxoplasma that was reported to be IgM positive. She contacted us distraught about this and was unable to reach her own doctors for months. With some delays and difficulties, sera were sent to the reference laboratory, where it was found that there was no IgM or IgG specific for Toxoplasma. This took about two weeks.
7. **Delays in diagnosis with what would have been preventable progressive harm to infant earlier and in 2022.**
   A 7-month old child was referred for strabismus and bilateral central macular scars. His and his mother's serologies identified recently acquired toxoplasma in the mother and congenital infection in the baby. Medicines to treat his congenital toxoplasmosis were initiated, and the family asked why no testing for Toxoplasma was done in the US by their obstetricianas done in the US by their obstetrician.

In **Table A** we present the line data and show the structure of our clinical Study 1. In **Table B** we show data demonstrating difficulties with false positives that ICT helps to solve. In **Table C** we present the line data from the use of two FDA cleared test systems we used for back up confirmation in a study with implementation of monthly screening and its acceptance. In **Table 2 in the main text** we show the perfect concordance of the earlier pink line ICT (Begeman et al,2017; Mahinc et al,2017; Chapey et al, 2017). We provide new unpublished studies in the South of France (**Tables 1, 2** and **Box 2**), herein in the main manuscript.

Table A. Study 1. Design and Data for 3 Testers with 5 Sera-Positive Persons for each tester, Each in Three Settings with Results Showing Their Primary Data in the Chicago Clinical Feasibility Implementation Trial 2020 to 2021. Corresponds to Fig 2A. This Study is Performed in Accordance with FDA and CLIA Guidelines and Regulations.

Subject ID	ICT blood test result	CLIA Test Result IgG	CLIA Test Result IgM	ICT serum test result	ICT Blood Tester	pregnancy status	gestation @consent	Subject ID	ICT blood test result	CLIA Test Result IgG	CLIA Test Result IgM	ICT serum test result	ICT Blood Tester	pregnancy status	gestation @consent
**1**	Negative	negative	negative	Negative	KL	P	37	**31**	Negative	negative	negative	Negative	KL	P	12
**2**	Negative	negative	negative	Negative	KL	P	28	**32**	Negative	negative	negative	Negative	KL	P	12
**3**	Negative	negative	inequivalent (ref lab neg)	Negative	KL	P	28	**33**	Negative	negative	negative	Negative	KL	P	11
**4**	Negative	negative	negative	Negative	KL	P	36	**34**	Negative	negative	negative	Negative	KL	P	14
**5**	Negative	negative	negative	Negative	KL	P	28	**35**	Negative	negative	negative	Negative	KL	P	11
**6**	Negative	negative	positive (ref lab neg)	Negative	KL	P	28	**36**	Negative	negative	negative	Negative	KL	P	10
**7**	Negative	negative	negative	Negative	KL	P	28	**37**	Positive	positive (>900 IU/ml)	negative	Positive	KL	NP	NA
**8**	Negative	negative	negative	Negative	KL	P	28	**38**	Positive	positive (131IU/ml)	negative	Positive	KL	NP	NA
**9**	Negative	negative	negative	Negative	KL	P	28	**39**	Negative	negative	negative	Negative	SS	NP	NA
**10**	Negative	negative	negative	Negative	KL	P	28	**40**	Negative	negative	negative	Negative	SS	NP	NA
**11**	Negative	negative	negative	Negative	KL	P	32	**41**	Negative	negative	negative	Negative	SS	NP	NA
**12**	N.D.				KL		28	**42**	Negative	negative	negative	Negative	SS	NP	NA
**13**	Negative	negative	negative	Negative	KL	P	30	**43**	Negative	negative	negative	Negative	SS	NP	NA
**14**	Negative	negative	negative	Negative	KL	P	8	**44**	Negative	negative	negative	Negative	SS	NP	NA
**15**	Negative	negative	negative	N.D.	KL	P	10	**45**	Positive	positive (405 IU/ml)	negative	Positive	SS	P	15
**16**	Negative	negative	negative	Negative	KL	P	10	**46**	Positive	positive (468 IU/ml)	negative	Positive	SS	NP	NA
**17**	Negative	negative	negative	Negative	KL	P	12	**47**	Positive	positive (227 IU/ml)	negative	Positive	SS	NP	NA
**18**	Positive	positive	negative	Positive	KL	NP	NA	**48**	Positive	positive (148 IU/ml)	negative	Positive	SS	NP	NA
**19**	Positive	positive (21IU/ml)	negative	Positive	KL	NP	NA	**49**	Positive	positive (310 IU/ml)	negative	Positive	SS	NP	NA
**20**	Positive	positive (64 IU/ml)	negative	Positive	KL	NP	NA	**50**	Positive	positive (22 IU/ml)	negative (0.8 AI)	Positive	MS	P	14
**21**	Negative	negative	positive (1.1)	Negative	KL	P	10	**51**	Negative	negative	negative	Negative	MS	P	11
**22**	Negative	negative	negative	Negative	KL	P	13	**52**	Positive	positive	negative	Positive	MS	P	10
**23**	Negative	negative	negative	Negative	KL	P	15	**53**	Negative	negative	negative	Negative	MS	P	15
**24**	Negative	negative	negative	Negative	KL	P	12	**54**	Negative	negative	negative	Negative	MS	P	12
**25**	Negative	negative	negative	Negative	KL	P	9	**55**	Negative	negative	negative	Negative	MS	P	5
**26**	Negative	negative	negative	Negative	KL	P	13	**56**	Negative	negative	negative	Negative	MS	P	13
**27**	Negative	negative	negative	Negative	KL	P	11	**57**	Negative	N.D.	N.D.	N.D.	MS	NP	NA
**28**	Negative	negative	negative	Negative	KL	P	10	**58**	Positive	positive (222 IU/ml)	Equivocal: IgM N.D.	Positive	MS	P	15
**29**	Negative	negative	negative	Negative	KL	P	9	**59**	Positive	positive (66 IU/ml)	negative	Positive	MS	NP	NA
**30**	Negative	negative	negative	Negative	KL	P	10	**60**	Positive	positive (617 U/ml)	negative	Positive	MS	NP	NA

Green column, MFM nurse, blue column Medicine Pediatrics Infectious Diseases MD, lavender column, Obstetrician; Pink marks test value for a false positive predicate test. ND, not done. Table corresponds to Figure 2A. Table corresponds with Figure 2A.

Table B. Study 2 Part 1 Lyon Reference Laboratory ICT Test Results for Sera from Pregnant Patients Referred by Local Physicians for T.*gondii* IgM with Predicate Tests in Local Laboratories and Negative Western Blot as Gold Standard Comparator. The organization and data correspond to Figure 2B.

Patient number	Sampling date	Gestation when tested (week)	ICT results	IgM	Test kit	WB IgM results	University hospital Lyon la Croix Rousse conclusion
1	9/8/21	unknown	Negative	3.84	Cobas	Negative	Negative
2	9/9/21	33	Negative	0.876	Cobas	Negative	Negative
3	12/28/21	34	Negative	3.26	Cobas	Negative	Negative
4	4/2/21	9	Negative	1.35	Cobas	Negative	Negative
5	12/20/21	32	Negative	1.19	Roche Immunoelectro	Negative	Negative
6	2/23/22	7	Negative	Equivocal	Roche Electrochimiluminescence	Negative	Negative
7	8/24/21	4	Negative	0.74	Abbot Alinity	Negative	Negative
8	9/20/21	4	Negative	1.3	Roche Immunoelectro	Negative	Negative
9	11/23/21	unknown	Negative	0.54	Abbot Alinity	Negative	Negative
10	12/30/21	11	Negative	0.98	Cobas	Negative	Negative
11	1/13/22	23	Negative	1.45	Roche Immunoelectro	Negative	Negative
12	9/16/21	unknown	Negative	Positive	Cobas	Negative	Negative
13	9/14/21	16	Negative	Positive	Cobas	Negative	Negative
14	4/8/21	11	Negative	0.72	Abbot Alinity	Negative	Negative
15	10/12/21	36	Negative	0.92	Abbot Alinity	Negative	Negative
16	2/17/22	9	Negative	1.44	Roche Immunoelectro	Negative	Negative
17	10/26/21	15	Negative	1.11	Centaur	Negative	Negative
18	10/15/21	15	Negative	1.68	Cobas	Negative	Negative
19	10/21/21	14	Negative	1	Centaur	Negative	Negative
20	10/27/21	10	Negative	0.867	Cobas	Negative	Negative
21	9/22/21	7	Negative	11.67	Roche	Negative	Negative
22	7/28/21	32	Negative	0.66	Abbot Alinity	Negative	Negative
23	8/11/21	10	Negative	Positive	Cobas	Negative	Negative
24	9/8/21	unknown	Negative	1.48	Cobas	Negative	Negative
25	8/20/21	3	Negative	0.6	Abbot Alinity	Negative	Negative
26	4/17/21	30	Negative	0.6	Architect	Negative	Negative
27	11/23/21	given birth	Negative	0.9	Cobas	Negative	Negative
28	8/3/21	3	Negative	4.14	Cobas	Negative	Negative
29	7/10/21	post birth	Negative	2.68	Centaur	Negative	Negative
30	10/13/21	17	Negative	1.01	Abbot Alinity	Negative	Negative
31	5/18/21	3	Negative	1.35	Roche	Negative	Negative
32	10/26/21	29	Negative	Equivocal	Roche	Negative	Negative

Table C. Study 3 Shows Concordance of ICT Results in Chicago Acceptability of Monthly Testing In Testing of Sera in Lyon Reference Laboratory Using Abbott Architect and for one person VIDAS IgG ELISA, and VIDAS G and M ELFA in Quindío Reference Laboratory. Initial tests in earlier months were all concordant with Abbott Architect and reported in Lykins et al [28]. This study corresponds to Figure 4 which shows the results of USA Acceptability, Study 3.

Colombia Reference Laboratory					France Reference Laboratory						
Patient Code	TOXO IgG VIDAS (IU/ml)	Result	TOXO IgM VIDAS (RFV)	Result	Architect IgG (IU/ml)*	Result**	LDBIO western blot Result	VIDAS Toxo G (IU/ml)	VIDAS Result	Architect IgM Index	Result
1-4	0	Neg	0.05	Neg	0.4	Neg				0.05	Neg
1-5	0	Neg	0.2	Neg	0.5	Neg				0.05	Neg
1-6	0	Neg	0.06	Neg	0.6	Neg				0.06	Neg
1-7	0	Neg	0.05	Neg	0.5	Neg				0.05	Neg
1-8	0	Neg	0.09	Neg	0.7	Neg				0.07	Neg
2-4	N/A				0.2	Neg				0.05	Neg
2-5	N/A				0.5	Neg				0.07	Neg
2-6	0	Neg	0.04	Neg	0.5	Neg				0.07	Neg
2-7	0	Neg	0.04	Neg	0.5	Neg				0.04	Neg
2-8	0	Neg	0.04	Neg	0.2	Neg				0.06	Neg
2-9	0	Neg	0.04	Neg	0.3	Neg				0.03	Neg
3-4	0	Neg	0.04	Neg	2.8	Eq		0	Neg	0.06	Neg
3-5	0	Neg	0.04	Neg	2.2	Eq		0	Neg	0.06	Neg
3-6	0	Neg	0.04	Neg	2.1	Eq	Neg	56	IQ	0.06	Neg
3-7	0	Neg	0.04	Neg	2.9	Eq		0	Neg	0.05	Neg
3-8	0	Neg	0.05	Neg	7.2	Pos		0	Neg	0.03	Neg
4-4	0	Neg	0.04	Neg	0.1	Neg				0.06	Neg
4-5	0	Neg	0.04	Neg	0.1	Neg				0.03	Neg
4-6	0	Neg	0.04	Neg	0.1	Neg				0.03	Neg
4-7	0	Neg	0.04	Neg	0.1	Neg				0.03	Neg
5-4	0	Neg	0.05	Neg	0.3	Neg				0.04	Neg
5-5	0	Neg	0.05	Neg	IQ		Neg			IQ	
5-6	0	Neg	0.05	Neg	0.3	Neg				0.04	Neg
5-7	0	Neg	0.04	Neg	0.4	Neg				0.07	Neg
5-8	0	Neg	0.14	Neg	0.4	Neg				0.04	Neg
6-4	2	Neg	0.05	Neg	0.2	Neg				0.04	Neg
6-5	2	Neg	0.05	Neg	0.3	Neg				0.04	Neg
6-6	2	Neg	0.05	Neg	0.2	Neg				0.06	Neg
6-7	2	Neg	0.04	Neg	0.2	Neg				0.07	Neg
7-4	0	Neg	0.04	Neg	0.4	Neg				0.06	Neg
7-5	N/A				0.3	Neg				0.05	Neg
7-6	0	Neg	0.04	Neg	0.5	Neg				0.04	Neg
7-7	0	Neg	0.03	Neg	0.4	Neg				0.03	Neg
7-8	0	Neg	0.03	Neg	0.5	Neg				0.05	Neg
8-4	0	Neg	0.05	Neg	IQ		Neg			0.05	Neg
8-5	0	Neg	0.06	Neg	0.4	Neg				0.07	Neg
8-6	0	Neg	0.06	Neg	0.4	Neg				0.04	Neg
8-7	0	Neg	0.06	Neg	0.3	Neg				0.07	Neg
9-4	0	Neg	0.08	Neg	IQ		Neg			IQ	
9-5	0	Neg	0.03	Neg	IQ		Neg			0.03	Neg
9-6	0	Neg	0.04	Neg	0.4	Neg				0.05	Neg
9-7	0	Neg	0.12	Neg	0.2	Neg				0.05	Neg
9-8	0	Neg	0.03	Neg	0.5	Neg				0.04	Neg
10-4	0	Neg	0.06	Neg	IQ		Neg			0.06	Neg
10-5	0	Neg	0.06	Neg	0.4	Neg				0.05	Neg
10-6	0	Neg	0.06	Neg	IQ		Neg			0.05	Neg
10-7	0	Neg	0.06	Neg	0.2	Neg				0.04	Neg

Colombia Reference Laboratory					France Reference Laboratory						
Patient Code	TOXO IgG VIDAS (IU/ml)	Result	TOXO IgM VIDAS (RFV)	Result	Architect IgG (IU/ml)*	Result**	LDBIO western blot Result	VIDAS Toxo G (IU/ml)	VIDAS Result	Architect IgM Index	Result
10-8	0	Neg	0.08	Neg	0.3	Neg				0.03	Neg
11-4	0	Neg	0.07	Neg	0.2	Neg				0.04	Neg
11-5	0	Neg	0.07	Neg	0.4	Neg				0.05	Neg
11-6	0	Neg	0.07	Neg	0.2	Neg				0.04	Neg
11-7	0	Neg	0.05	Neg	IQ		Neg			0.09	Neg
11-8	N/A	N/A	N/A	N/A	0.2	Neg				0.06	Neg
12-4	0	Neg	0.04	Neg	IQ		Neg			0.07	Neg
12-5	0	Neg	0.04	Neg	IQ		Neg			IQ	
12-6	0	Neg	0.04	Neg	0.2	Neg				0.07	Neg
12-7	0	Neg	0.04	Neg	0.5	Neg				0.07	Neg
13-4	0	Neg	0.05	Neg	0.3	Neg				0.04	Neg
13-5	0	Neg	0.04	Neg	IQ		Neg			0.03	Neg
13-6	0	Neg	0.05	Neg	IQ		Neg			0.05	Neg
13-7	0	Neg	0.06	Neg	0.2	Neg				0.05	Neg
13-8	0	Neg	0.05	Neg	0.2	Neg				0.06	Neg
13-9	0	Neg	0.03	Neg	0.3	Neg				0.06	Neg
14-4	0	Neg	0.03	Neg	0.1	Neg				0.03	Neg
14-5	0	Neg	0.05	Neg	IQ		Neg			0.06	Neg
14-6	0	Neg	0.04	Neg	IQ		Neg			0.03	Neg
14-7	0	Neg	0.04	Neg	IQ		Neg			0.05	Neg
14-8	0	Neg	0.16	Neg	0.2	Neg				0.02	Neg
15-4	0	Neg	0.04	Neg	0.3	Neg				0.07	Neg
15-5	0	Neg	0.03	Neg	IQ		Neg			0.04	Neg
15-6	0	Neg	0.04	Neg	0.2	Neg				0.07	Neg
15-7	0	Neg	0.03	Neg	0.2	Neg				0.04	Neg
15-8	N/A	N/A	N/A	N/A	0.2	Neg				0.07	Neg
16-4	0	Neg	0.05	Neg	0.1	Neg				0.07	Neg
16-5	0	Neg	0.05	Neg	IQ		Neg			IQ	
16-6	0	Neg	0.04	Neg	0.2	Neg				0.08	Neg
16-7	0	Neg	0.05	Neg	0.2	Neg				0.08	Neg
16-8	0	Neg	0.05	Neg	0.1	Neg				0.07	Neg
16-9	0	Neg	0.04	Neg	0.1	Neg				0.07	Neg
17-4	0	Neg	0.02	Neg	0.3	Neg				0.07	Neg
17-5	0	Neg	0.02	Neg	0.4	Neg				0.07	Neg
17-6	0	Neg	0.02	Neg	0.5	Neg				0.03	Neg
17-7	0	Neg	0.06	Neg	IQ		Neg			IQ	
17-8	0	Neg	0.02	Neg	IQ		Neg			IQ	
18-4	1	Neg	0.03	Neg	0.2	Neg				0.02	Neg
18-5	0	Neg	0.04	Neg	0.3	Neg				0.06	Neg
18-6	0	Neg	0.03	Neg	0.3	Neg				0.03	Neg
18-7	0	Neg	0.03	Neg	0.3	Neg				0.06	Neg
19-4	0	Neg	0.04	Neg	0.2	Neg				0.04	Neg
19-5	0	Neg	0.05	Neg	0.3	Neg				0.07	Neg
19-6	0	Neg	0.05	Neg	0.1	Neg				0.07	Neg
19-7	0	Neg	0.05	Neg	0.2	Neg				0.04	Neg
19-8	0	Neg	0.05	Neg	0.2	Neg				0.05	Neg

We provide an historical overview with a timeline (**Fig A**) and a Figure showing what we encountered for many decades before carrying out the studies herein (**Fig B**). Our goal in performing the studies described herein is to move beyond the harm we have observed from untreated congenital toxoplasmosis.

Fig A. Overview Summary of studies herein in context of other work. Top presents context of current studies toward introducing screening in a global initiative. Red font shows work herein. **Fig B** summarizes the studies herein.

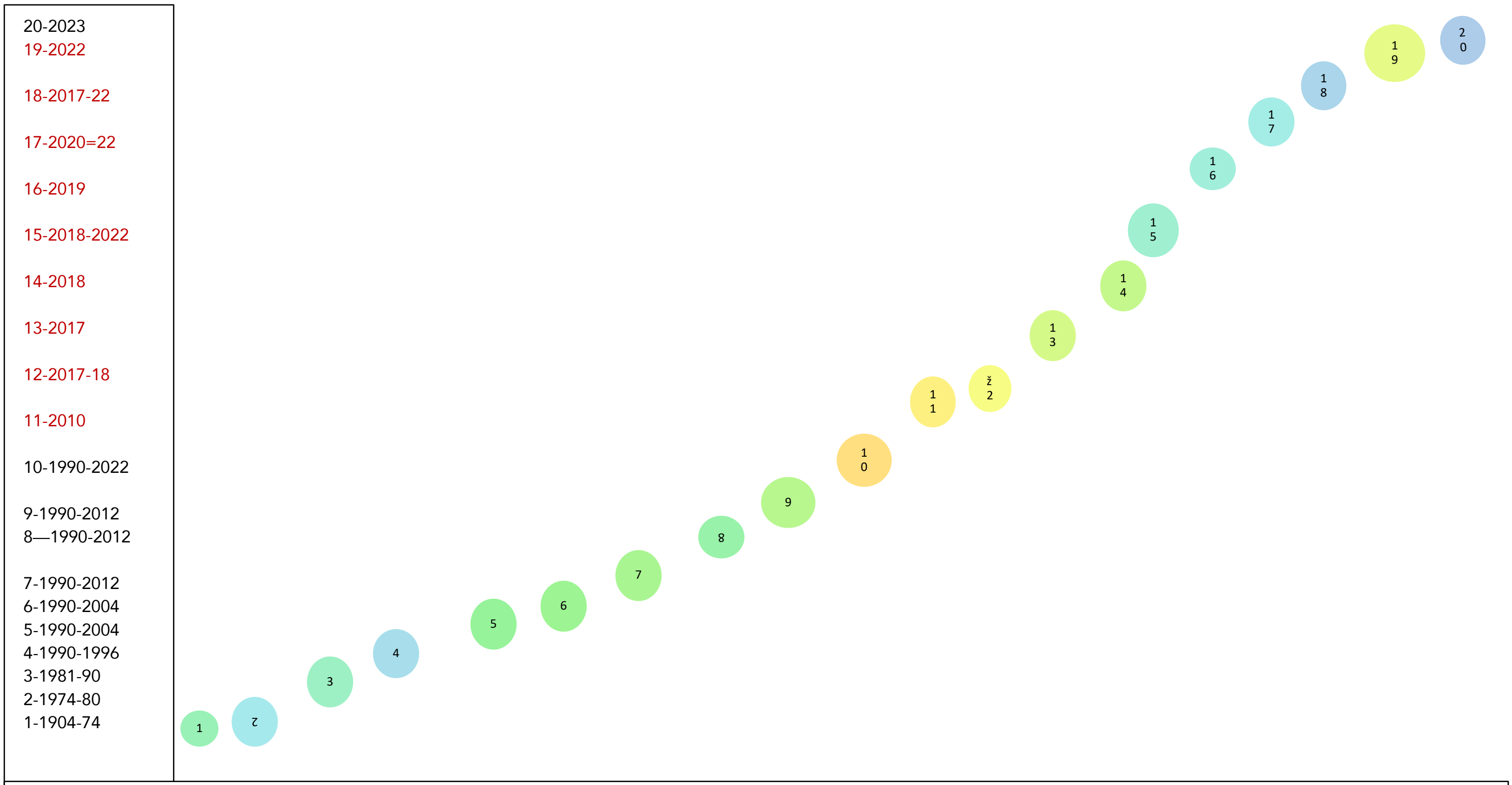

20-2023 **Goal: Possible initiation of prenatal screening and treatment systematically in USA**

19-2022. **Submitting to FDA CLIA; Effect on brain, Signature pathways. Alzgerm award re microbial pathogenesis of Alzheimer's disease.**

18-2017-22 **Reduced to practice USA-New paradigm."Revolutionary" (Studies 1 to 12*,*see Box 2*)**

17-2020-22. **Acceptability USA, Colombia (Study 3)**

16-2019 **CE Mark;; Large data analysis, Epidemiology, Education, Public health programs; WB highly sensitive specific as back up test**

15-2018-2022 **Studies 1-12 with ICT toward FDA CLIA;** POC implementation to facilitate rx in USA, Morocco, Colombia, France-results replicate

14-2018. **RCT France; early PSL vs spiramycin prevents neurologic damage significantly; Published late 2018; discontinued -trend reduced infection**

13-2017. **Rapid serum tests; ASSURED POC on whole blood. Nano gold saliva; spillover benefit -especially when used in health care setting**

12-2017-18 **EMSCOT leads to stop newborn/ gestational screening some Europe; UK not screen,close Institut Puericulture; Kieffer,Wallon early benefit**

11-2010. **Springfield proposed law.Suggestion cost/benefit mathematical model, USA; Later applied to Austria,**
France (2022) **Benefit in all. Implementation.**

10-1990-2022 **Amish, Brazil, Florida, Hawaii, California; Canada, Guiana, hypervirulent, Amazon tributaries;**
Differences by genetics at birth,later obviated with Rx

9-1990-2012 **Severe disease on all continents.Epidemic,endemic, waterborne,areas of higher and lower prevalence,not monomorphic by country;;**

8--1990-2012 **Parasite Genetic diversity in USA,South America**

7-1990-2012 **Genetic susceptibility associated with prematurity, severity, patterns of hydrocephalus, lower socioeconomic status,family clusters**

6-1990-2004. **Gestational screening**-once a trimester, introduce monthly, rx first trimester,14 weeks, amniocentesis PCR, continuous PSL

5-1990-2004 **Randomized control trial, pre-specified endpoints** higher, lower dose Pyrimethamine with SL

4-1990-1996 **Understanding** infection: acute acquired, pre-natal, congenital, ocular, immune compromise;Rx PSL Phase I CT

3-1981-90 **Congenital infection (CT)** France alternate spiramycin, PSL, Austria courses of PSL **Immune compromise;deficiency** PSL

2-1974-80 Laws in France and Austria mandate prenatal diagnosis-(not systematic monthly yet), and treatment(less standardized).

1-1904-74 Discovery of *Toxoplasma;* recognition that acquired prenatally causes severe disease in the USA, Europe, South America

[illegible]

Fig B. Historical perspectives on screening and treatment of *Toxoplasma gondii* acquired in Gestation in France and the USA. This figure is to provide context of where we have come from to studies herein with considerable spillover benefit for patient care and well-being, with the goal that studies herein will provide a foundation for improvement of prevention and care for congenital toxoplasmosis.

"There is a parasite which is harmless for you [now not certain that is accurate, RMc] but which can cause severe impairments in your child if you become infected during your pregnancy. You will notice nothing but we could tell you if you are immune. If you are not we can monitor you during pregnancy. If you become infected we can detect the infection in your infant even if he looks normal; we can even detect it in utero and treat without delay. Yet, we will do nothing, since all that is too expensive. But do not worry. Perhaps you are immune--and if you are not, you will probably escape infection when pregnant. If the worst does happen, your fetus might escape. And if your infant is severely impaired, there is no risk in having another baby because you will now be immune. Nevertheless, try not to get infected. Eat well cooked meat and wash your hands. Good luck."

Georges Desmonts, Preventing Congenital Toxoplasmosis. The Lancet . 336:1017-18.October 20, 1990

"Toxoplasmosis is a serious disease in persons with the congenital infection and in immunocompromised patients. The tragedy of toxoplasmosis as a cause of blindness or near-blindness or of psychomotor or mental retardation in children and the fact that it can be prevented have never concerned the medical community in the United States as much as in Europe. Almost 15 years ago Desmonts and Couvreur of Paris defined in the Journal the risk and range of severity of congenital toxoplasmosis, the value of systematic screening and patient education, and the value of treatment of the mother during pregnancy to prevent transmission."

R. McCabe and JS Remington, Toxoplasmosis: The Time has Come NEJM, 1988

"Imagine with me a young mother, happily expecting her first child. She does everything she knows to secure the baby's health, then everything goes wrong. Good morning Ladies and Gentleman .My family and I are here to show you the reality of what toxoplasmosis can do if it goes untreated. We offer our story in hope that you will.....do all you can to prevent the overwhelming effects…."…; And I could have had a simple test" J.Morel, et al *Springfield* (Regarding Prenatal and Neonatal Congenital Toxoplasmosis Prevention and Treatment Act SB3667: Submitted to the Committee on Public Health Illinois State Senate) 2010; and J.Morel in captioned photograph in Begeman et al 2017 , PLoS NTD

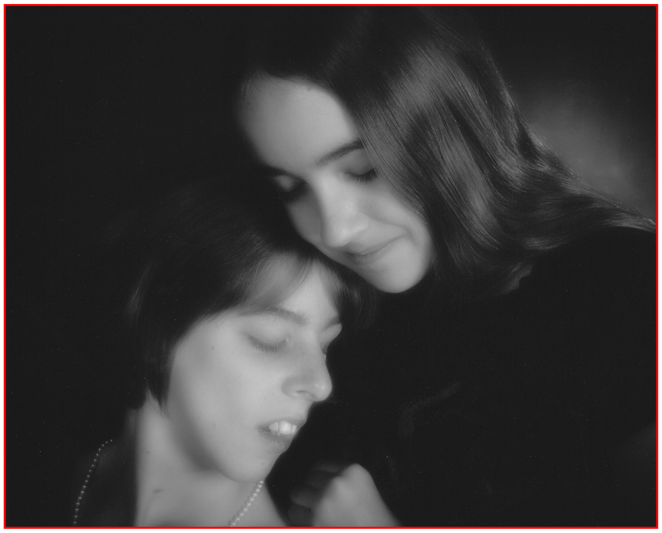

"Toxoplasmosis stole my daughter's full potential. With current possibilities for screening, vaccination and treatments, beautiful dreams could have become reality." J. Morel

2022

"Implementation of this one testing protocol can promote spillover benefits that break a vicious cycle of poor access to health care and poor outcomes." Stillwaggon in Begeman et al PLoSNTD 2017

In France screening was mandated by law. In Austria those screened received additional health care benefits. In Colombia it was introduced through practice societies. In the USA those in advisory positions recommended that education, easy feasibility, low cost would result in those who would benefit choosing to have testing incorporated in medical practice and USA patient culture at many levels by personal preference. The acceptability study demonstrated that informed patients would want this and obstetricians could use this comfortably and without inconvenience in their practice. It could easily be introduced into family practice and adolescent pediatric care to identify seropositive patients at risk of this most common form of retina disease due to infection and loss of sight. Such screening in adolescence could also provide pre-pregnancy testing for young women to allow knowledge of who is seronegative and should be screened during pregnancy. Pre-marital/conception screening as initially occurred in France could also be helpful as families plan to have children. As *Toxoplasma* has been transmitted by organ donation and white blood cell transfusion and by sperm in domestic non-human animals, and can relapse with immune suppression and may be causative for epilepsy, and some neurodegeneration, has caused epidemics in North, Central, and South America, and has been categorized by NIH as a potential bioterrorism pathogen, and a neglected tropical disease, there are a number of other medical settings where knowledge of *Toxoplasma* serologic status may be useful.

Obstetricians, nurse midwives, family practitioners, obstetrical nurses, and other obstetrical providers are uniquely positioned to intervene to prevent this disease, to improve the health of both mother and child. POC test-based monthly gestational screening of seronegative patients for *T. gondii* infection provides a valuable tool in the obstetric armamentarium to ensure maternal-child wellness. When such tests have undergone appropriate evaluation by the FDA and CLIA, as they have undergone in the CE-mark evaluation and approval in Europe, this testing can enable a paradigm shift in our management of the risks associated with exposure to *T. gondii.*

Funding: Funding was from the Medical Student Award, the National Institute of Diabetes and Digestive and Kidney Diseases for their Grant #T35DK062719-30 (AG), the National Institutes of Health for their Division of Microbiology and Infectious Diseases Grant to RMc #R01 AI2753,RO1 16945, AI08749-01A1 BIOL-3 , U01 AI77887, U01 AI082180, TMP R01-AI071319, the Thrasher Children's Charity Research Fund for their E.W. "Al" Thrasher Award, the Kiphart Global-Local Health Seed Fund Award (to RMc), University of Chicago). We are grateful to Taking out Toxo, Network for Good, Toxoplasmosis Research Institute, The Cornwell Mann Family Foundation, The Rodriguez family, The Samuel family and Running for Fin, the Morel, Rooney, Mussalami, Kapnick, Taub, Engel, Harris, Drago, Longfellow/Van Dusen families, and the study participants.

References

1. McLeod R, Kieffer F, Sautter M, Hosten T, Pelloux H. Why prevent, diagnose and treat congenital toxoplasmosis? Mem Inst Oswaldo Cruz 2009; 104:320-44.
2. McLeod R, Lykins J, Noble AG, Rabiah P, Swisher C, Heydemann P, *et al* Management of Congenital Toxoplasmosis. Curr Pediatr Rep 2014; 2:166-194
3. Mandelbrot L, Kieffer F, Sitta R, Laurichesse-Delmas H, Winer N, Mesnard L, *et al.* Prenatal therapy with pyrimethamine + sulfadiazine vs spiramycin to reduce placental transmission of toxoplasmosis: a multicenter, randomized trial. Am J Obstet Gynecol 2018; 219(4): 386.e1-386.e9. https://doi.org/10.1016/j.ajog.2018.05.031 PMID: 29870736
4. Pappas G, Roussos N, Falagas ME. Toxoplasmosis snapshots: global status of Toxoplasma gondii seroprevalence and implications for pregnancy and congenital toxoplasmosis. Int J Parasitol 2009; 39:1385-1394. https://doi.org/10.1016/j.ijpara.2009.04.003 PMID: 19433092
5. Torgerson PR, Mastroiacovo P. The global burden of congenital toxoplasmosis: a systematic review. Bull World Health Organ 2013; 91:501-508. https://doi.org/10.2471/BLT.12.111732 PMID: 23825877
6. McLeod R, Van Tubbergen C, Boyer KM. Toxoplasmosis (Toxoplasma gondii). In: Kliegman R, St. Geme J, eds. Nelson Textbook of Pediatrics. 20th ed. Elsevier, 2016: 1723-1733. New edition in press 20*24.*
7. McLeod R, Cohen W, Dovgin S, Finkelstein L, Boyer KM. Human Toxoplasma Infection. In: Weiss L, Kim K, eds. Toxoplasma Gondii. 3rd ed. Elsevier, 2020:117-228.
8. El Bissati K, Levigne P, Lykins J, Adlaoui EB, Barkat A, Berraho A, *et al*. Global initiative for congenital toxoplasmosis: an observational and international comparative clinical analysis. Emerg Microbes Infect 2018; 7:165. https://doi.org/10.1038/s41426-018-0164-4 PMID: 30262847
9. Wallon M, Peyron F, Cornu C, Vinault S, Abrahamowicz M, Kopp CB, *et al*. Congenital Toxoplasma Infection: Monthly Prenatal Screening Decreases Transmission Rate and Improves Clinical Outcome at Age 3 Years. Clin Infect Dis 2013; 56:1223-1231. https://doi.org/10.1093/cid/cit032. PMID: 23362291
10. Gomez-Marin JE, Montoya-de-Londono MT, Castano-Osorio JC. A maternal screening program for congenital toxoplasmosis in Quindio, Colombia and application of mathematical models to estimate incidences using age-stratified data. Am J Trop Med Hyg. 1997; 57(2):180-6. https://doi.org/10.4269/ajtmh.1997.57.180 PMID: 9288813.
11. Carneiro AC, Machado AS, Béla SR, Costa JG, Andrade GM, Vasconcelos-Santos DV, *et al*. Reply to Gómez Marín. J Infect Dis 2016; 214(4):656. https://doi.org/10.1093/infdis/jiw211 PMID: 27357342.
12. McLeod R, Boyer KM, Lee D, Mui E, Wroblewski K, Karrison T, *et al*. Prematurity and severity are associated with Toxoplasma gondii alleles (NCCCTS, 1981-2009). Clin Infect Dis. 2012; 54(11):1595-605. https://doi.org/10.1093/cid/cis258 PMID: 22499837

13. Peyron F, McLeod R, Ajzenberg D, Contopoulos-Ioannidis DG, Kieffer F, Mandelbrot L, *et al*. Congenital Toxoplasmosis in France and the United States: One Parasite, Two Diverging Approaches. PLoS Negl Trop Dis 2017; 11:e0005222. https://doi.org/10.1371/journal.pntd.0005222 PMID: 28207736

14. Stillwaggon E, Carrier CS, Sautter M, McLeod R. Maternal Serologic Screening to Prevent Congenital Toxoplasmosis: A Decision-Analytic Economic Model. PLoS Negl Trop Dis 2011; 5:e1333. https://doi.org/10.1371/journal.pntd.0001333 PMID: 21980546

15. Prusa A-R, Kasper DC, Sawers L, Walter E, Hayde M, Stillwaggon E. Congenital toxoplasmosis in Austria: Prenatal screening for prevention is cost-saving. PLoS Negl Trop Dis. 2017; 11:e0005648. https://doi.org/10.1371/journal.pntd.0005648 PMID: 28692640

16. Binquet C, Lejeune C, Seror V, Peyron F, Bertaux AC, Scemama O, *et al*. The cost-effectiveness of neonatal versus prenatal screening for congenital toxoplasmosis. PLoS One. 2019; 14(9):e0221709. https://doi.org/10.1371/journal.pone.0221709 PMID: 31532766

17. Sawers L, Wallon M, Mandelbrot L, Villena I, Stillwaggon E, Kieffer F. Prevention of congenital toxoplasmosis in France using prenatal screening: A decision-analytic economic model. PLoS One. 2022 Nov 4;17(11):e0273781. doi: 10.1371/journal.pone.0273781. PMID: 36331943; PMCID: PMC9635746.

18. Maldonado YA, Read JD, Committee on Infectious Diseases. Diagnosis, treatment, and prevention of congenital toxoplasmosis in the United States. Pediatrics 2017; 139(2):e20163860. https://doi.org/10.1542/peds.2016-3860 PMID: 28138010

19. Kosack CS, Page AL, Klatser PR. A guide to aid the selection of diagnostic tests. Bull World Health Organ. 2017;95(9):639-45.

20. Liesenfeld O, Press C, Montoya JG, Gill R, Isaac-Renton JL, Hedman K, *et al*. False-positive results in immunoglobulin M (IgM) toxoplasma antibody tests and importance of confirmatory testing: the Platelia Toxo IgM test. J Clin Microbiol. 1997 Jan;35(1):174-8. doi: 10.1128/JCM.35.1.174-178.1997. PMID: 8968902; PMCID: PMC229533.

21. Garry DJ, Elimian A, Wiencek V, Baker DA. Commercial laboratory IgM testing for Toxoplasma gondii in pregnancy: a 20-year experience. Infect Dis Obstet Gynecol. 2005 Sep;13(3):151-3. doi: 10.1080/10647440500148024. PMID: 16126500; PMCID: PMC1784571.

22. Dhakal R, Gajurel K, Pomares C, Talucod J, Press C, Montoya JG. Significance of a Positive *Toxoplasma* Immunoglobulin M Test Result in the United States. J Clin Microbiol 2015; 53(11):3601-05. https://doi.org/10.1128/JCM.01663-15 PMID: 26354818

23. Lykins JD, Li X, Levigne P, Zhou Y, El Bissati K, Clouser F, *et al*. Rapid, inexpensive, fingerstick, whole-blood, sensitive, specific, point-of-care test for anti-Toxoplasma antibodies. PLoS Negl Trop Dis 2018; 12:e0006536. https://doi.org/10.1371/journal.pntd.0006536 PMID: 30114251

24. Begeman IJ, Lykins J, Zhou Y, Lai BS, Levigne P, El Bissati K, et al. Point-of-care testing for Toxoplasma gondii IgG/IgM using Toxoplasma ICT IgG-IgM test with sera from the United States and implications for developing countries. PLoS Negl Trop Dis 2017; 11:e0005670. https://doi.org/10.1371/journal.pntd.0005670 PMID: 28650970

25. Chapey E, Wallon M, Peyron F. Evaluation of the LDBIO point of care test for the combined detection of toxoplasmic IgG and IgM. Clin Chim Acta 2017; 464:200–201. https://doi.org/10.1016/j.cca.2016.10.023 PMID: 27765564

26. Mahinc C, Flori P, Delaunay E, Guillerme C, Charaoui S, Raberin H, *et al*. Evaluation of a New Immunochromatography Technology Test (LDBio Diagnostics) To Detect Toxoplasma IgG and IgM: Comparison

with the Routine Architect Technique. J Clin Microbiol 2017; 55:3395-3404. https://doi.org/10.1128/JCM.01106-17 PMID: 28954897

27. Gomez CA, Budvytyte LN, Press C, Zhou L, McLeod R, Maldonado Y, et al. Evaluation of Three Point-of-Care Tests for Detection of Toxoplasma Immunoglobulin IgG and IgM in the United States: Proof of Concept and Challenges. Open For Inf Dis 2018; 5:ofy215. https://doi.org/ 10.1093/ofid/ofy215 PMID: 30393749

28. Lykins J, Wang K, Wheeler K, Clouser F, Dixon A, El Bissati K, *et al*. Understanding Toxoplasmosis in the United States Through "Large Data" Analyses. Clin Inf Dis 2016; 63(4): 468-75. https://doi. org/10.1093/ci/ciw356 PMID: 27353665

29. El Mansouri B, Amarir F, Peyron F, Adlaoui EB, Piarroux R, El Abassi M, *et al*. Performance of a novel point-of- care blood test for Toxoplasma infection in women from diverse regions of Morocco. Microbes Infect 2021 Dec;10(1):1675-1682. doi:10.1080/22221751.2021.1948359.

30. Abraham S, Piarroux R. Zhou Y, Tesic V, Abeleda A , Houhou-Fidouh N,et al European Journal of Clinical Microbiology & Infectious Diseases : Official Publication of the European Society of Clinical Microbiology, 26 Sep 2023, 42(11):1327-1335 https://doi.org/10.1007/s10096-023-04669-8 , PMID: 37749274

31. Ben-Abdallah R, Kalboussi Y, Bellali H, Issaoui N, Souissi O, Maatoug R et al. , Contribution of the *Toxoplasma* ICT IgG IgM® test in determining the immune status of pregnant women against toxoplasmosis. J Laboratory Clinical Analysis.35, 2021 e23749 DOI 10.1002/jcla 23749 2021

32. Felin MS . Wang K . Moreira A . Grose, A . Leahy K . Zhou Y,et al Building Programs to Eradicate Toxoplasmosis Part I: Introduction and Overview. Curr Pediatr Rep. 2022;10(3):57-92. doi: 10.1007/s40124-022-00269-w. Epub 2022 Aug 22. PMID: 36034212; PMCID: PMC9395898.

33. Felin MS . Wang K, Moreira A, Grose3, A . Leahy K . Zhou Y,et al. Building Programs to Eradicate Toxoplasmosis Part II: Education. Curr Pediatr Rep. 2022 Sep;10(3):93-108. doi: 10.1007/s40124-022-00267-y. Epub 2022 Aug 1. PMID: 36969368; PMCID: PMC10035399

34. Felin MS. Wang K . Raggi C, Moreira A , Pandey A, Grose, A et al . Building Programs to Eradicate Toxoplasmosis Part III: Epidemiology and Risk Factors. Curr Pediatr Rep. 2022 Sep;10(3):109-124. doi: 10.1007/s40124-022-00265-0. Epub 2022 Jun 22. PMID: 37744780; PMCID: PMC1051631

35. Felin MS . Wang K . Moreira A . Grose, A . Leahy K . Zhou Y,et al Building Programs to Eradicate Toxoplasmosis Part IV: Understanding and Development of Public Health Strategies and Advances "Take a Village". Curr Pediatr Rep. 2022;10(3):125-154. doi: 10.1007/s40124-022-00268-x. Epub 2022 Aug 16. PMID: 35991908; PMCID: PMC9379243

36. Flores C, Villalobos-Cerrud D, Borace J, Mcleod R, Fábrega L, NoreroX, *et al*. Prevalence and risk factors associated with *T. gondii* infection in pregnant women and newborns from Panama Pathogens. 2021 Jun17;10(6):764. doi: 10.3390/pathogens10060764.

37. Pomares C, Zhang B, Arulkumar S, Gonfrier G, Marty P, Zhao S, et al. Validation of IgG, IgM multiplex plasmonic gold platform in French clinical cohorts for the serodiagnosis and follow-up of *Toxoplasma gondii* infection.Diagn Microbiol Infect Dis. 2017 Mar;87(3):213-218. doi:10.1016/j.diagmicrobio.2016.09.001. Epub 2016 Sep 8. PMID: 28040304

38. Public Health Service, Department of Health and Human Services (US), Food and Drug Administration. (1997). *FDA public health advisory: limitations of toxoplasmosis IgM commercial test kits (letter)*Washington Department of Health and Human Services (US)

39. McLeod R., Sautter M,, Rooney T, Morel J, Taub L, Taub D, *et al* Submission to the Committee on Public Health Illinois State Senate. Regarding: Prenatal and Neonatal Congenital Toxoplasmosis Prevention and Treatment Act, SB3667 in the context of the National Collaborative Chicago Based Congenital Toxoplasmosis Study. April 13. 2010.

40. Franck J, Garin YJ-F, Dumon H. LDBio-Toxo II Immunoglobulin G Western Blot Confirmatory Test for Anti-T Toxoplasma Antibody Detection. J Clin Microbiol. 2008 Jul 1;46(7):2334–8.

41. Maudry A, Chene G, Chatelain R, Patural H, Bellete B, Tisseur B, et al. Bicentric Evaluation of Six Anti-Toxoplasma Immunoglobulin G (IgG) Automated Immunoassays and Comparison to the Toxo II IgG Western Blot. Clin Vaccine Immunol. 2009 Sep 1;16(9):1322–6.

42. Jost C, Touafek F, Fekkar A, Courtin R, Ribeiro M, Mazier D, et al. Utility of immunoblotting for early diagnosis of t. oxoplasmosis seroconversion in pregnant women. Clin Vaccine Immunol CVI. 2011 Nov;18(11):1908–12.

43. Villard O, Cimon B, L'Ollivier C, Fricker-Hidalgo H, Godineau N, Houze S, et al. Serological diagnosis of *Toxoplasma gondii* infection: Recommendations from the French National Reference Center for Toxoplasmosis. Diagn Microbiol Infect Dis. 2016 Jan;84(1):22–33.

44. Douet T, Armengol C, Charpentier E, Chauvin P, Cassaing S, Iriart X, et al. Performance of seven commercial automated assays for the detection of low levels of anti-Toxoplasma IgG in French immunocompromised patients. Parasite. 2019;26:51.

45. Simon L, Fillaux J, Guigon A, Lavergne R-A, Villard O, Villena I, et al. Serological diagnosis of *Toxoplasma gondii*: analysis of false-positive IgG results and implications. Parasite Paris Fr. 2020;27:7.

46. Boyer K, Hill D, Mui E, Wroblewski K, Karrison T, Dubey JP, et al. Unrecognized Ingestion of Toxoplasma gondiiOocysts Leads to Congenital Toxoplasmosis and Causes Epidemics in North America Clin Infect Dis. 2011 Dec 1; 53(11): 1081–1089. Published online 2011 Oct 21. doi: 10.1093/cid/cir667; PMCID: PMC3246875 PMID:

47. Londoño-Mrtinez JC, Velasco -Velasquez S, Cordero-Lopez S, Osorio MF, Celis-Giraldo D, Thiobodeau J, et al. Evaluation of the acceptability of point of care diagnostic test for prenatal toxoplasmosis(Translational Research Phase II) Journal of Infection and Public Health 22 November 2022.16(1):15-24. PMID: 36446203 https://www.sciencedirect.com/science/article/pii/S1876034122003185

48. Mejia-Oquendo M, Marulanda-Ibarra E, Gomez-Marin JE, Mejia-Oquendo M, Marulanda-Ibarra E, Gomez-Marin JE. Evaluation of the impact of the first evidence-based guidelines for congenital toxoplasmosis in Armenia (Quindío) Colombia: An observational retrospective analysis-NC-ND license (http://creativecommons. org/licenses/ by-nc-nd/4.0/) 2021.
https://doi.org/10.1016/j.lana.2021.10

49. Plazas MI, Marín JS, Torres E, Londoño JC, Celis-Giraldo D, Marín JEG. Frequency of natural antibodies and concordance analysis for anti-TOXOPLASMA IgM tests in Colombian sera of pregnant women. Diagnostic Microbiology and Infectious Disease 2022;103:115733. https://doi.org/10.1016/J.DIAGMICROBIO.2022.115733

50. Torres-Morales E, Gómez-Marín JE. Evaluating a toxoplasma IgG avidity ELISA test for diagnostic purposes during pregnancy and correlating it with toxoplasma IgM and IgA in the Biomedical Research Centre's laboratory at the Universidad del Quindío, 2008. Revista Colombiana de Obstetricia y Ginecologia 2008;59.

51. McPhillie MJ, Zhou Y, Hickman MR, Gordon JA, Weber CR, Li Q, et al. Potent Tetrahydroquinolone Eliminates Apicomplexan Parasites. Front Cell Infect Microbiol. 2020 Jun 9;10:203. doi: 10.3389/fcimb.2020.00203. PMID: 32626661; PMCID: PMC7311950.

52. El Bissati K, Zhou Y, Paulillo SM, Raman SK, Karch CP, Reed S, et al. Engineering and characterization of a novel Self Assembling Protein for Toxoplasma peptide vaccine in HLA-A*11:01, HLA-A*02:01 and HLA-B*07:02 transgenic mice. Sci Rep. 2020 Oct 12;10(1):16984. doi: 10.1038/s41598-020-73210-0. PMID: 33046728; PMCID: PMC7552409.

53. Strules J, Dawant T, Riese K, Gerhold R, Brown J, Olfenbuttel C, et al. Use of point of care test to determine the prevalence of antibodies to Toxoplasma gondii in black bears from North Carolina and Pennsylvania. J Parasitol. 2023 May 1;109(3):221-224. doi: 10.1645/22-72. PMID: 37327396.

54. Fasquelle F, Scuotto A , Vreulx A-C , Petit T , Charpentier T, Betbeder D. Nasal vaccination of six squirrel monkeys (Saimiri sciureus): Improved immunization protocol against *Toxoplasma gondii* with a nanoparticle-born vaccine. International Journal for Parasitology: Parasites and Wildlife International Journal for Parasitology: Parasites and Wildlife. 22, 2023: 69-74.

<u>List of Tables and Figures and their titles</u>

Box A. Case vignettes provide representative practical examples from false negative and false positive *Toxoplasma gondii* IgM tests in the USA that harm patients and patient care

Table A. Study 1. Design and Data for 3 Testers with 5 Sera-Positive Persons for each tester, Each in Three Settings with Results Showing Their Primary Data in the Chicago Clinical Feasibility Implementation Trial 2020 to 2021. Corresponds to Figure 2A. This Study is Performed in Accordance with FDA and CLIA Guidelines and Regulations.

Table B. Study 2 Part 1 Lyon Reference Laboratory ICT Test Results for Sera from Pregnant Patients Referred by Local Physicians for T.*gondii* IgM with Predicate Tests in Local Laboratories and Negative Western Blot as Gold Standard Comparator. The organization and data correspond to Figure 2B.

Table C. Study 3 Shows Concordance of ICT Results in Chicago Acceptability of Monthly Testing In Testing of Sera in Lyon Reference Laboratory Using Abbott Architect and for one person VIDAS IgG ELISA, and VIDAS G and M ELFA in Quindío Reference Laboratory. Initial tests in earlier months were all concordant with Abbott Architect and reported in Lykins et al [28]. This study corresponds to Figure 4 which shows the results of USA Acceptability, Study 3.

Figure A. Overview Summary of studies herein in context of other work. Top presents context of current studies toward introducing screening in a global initiative. Red font shows work herein. Figure S7 summarizes the studies herein

Figure B. Historical perspectives on screening and treatment of *Toxoplasma gondii* acquired in Gestation in France and the USA. This figure is to provide context of where we have come from to studies herein with considerable spillover benefit for patient care and well-being, with the goal that

studies herein will provide a foundation for improvement of prevention and care for congenital toxoplasmosis.
