# Supplementary material for: Novel paradigm enables accurate monthly gestational screening to prevent congenital toxoplasmosis and more
Source: PLoS Negl Trop Dis. 2024 May 28;18(5):e0011335. doi: 10.1371/journal.pntd.0011335 (PMC11132520; doi:10.1371/journal.pntd.0011335)
Supplement: S1 Text — (DOCX) [file pntd.0011335.s002.docx]

**Disclosures and Insuring Objectivity in Results**:

LDBio Diagnostics provided the ICT and Western Blots used in the studies. ANNAR Labs (Colombia) donated the AdBio kits. For the predicate test, costs for the comparison test for 58 persons for the feasibility, clinical trial the cost of performing the Bio-Rad IgG and IgM tests was provided by the Susan and Richard family Kiphardt Seed Fund and The Thrasher Children’s Charity. At LDBIO Diagnostics, Denis Limonne Pharm D. is the scientist and CEO share holder and Raphael Piarroux PharmD, PhD was the R&D Director Scientist until January 13, 2023. A patent application was submitted by D. Limonne with the scientists at the University of Chicago and in France in August 2018. This application is pending review in the United States in accordance with US Bayh Dole laws. This is for the development of the whole blood point of care test and the practical clinical utility of the ICT to guide treatment for gestational infection to prevent congenital toxoplasmosis. This is to insure its continued high-quality performance and reproducibility of the results described herein. It is pending in review at the US patent office.

In this collaborative work, the scientists D. Limonne and R. Piarroux (DL, RP) at LDBIO provided insights and knowledge from their earlier work in creating the ICT, and collaboratively with RMc discussed FDA and CLIA requirements with RMc and the FDA during an IDE and “presubQ” phase of this study. In this phase, the FDA Program provided guidance for this academic / Biotek collaboration to prepare materials to allow FDA review for dual 510K clearance and CLIA waiver for use of the ICT in the USA. RP of LDBIO performed the analysis of the French Blood bank serum to establish that the correct dilution required by CLIA instructions was 1:89. DL and RP designed the instruction sheet with input from FDA, CLIA, and RMc to be tested in the “QI at limits of detection study”. This was perfected in the “presubQ” process with advice from the FDA and CLIA as the FDA indicated that a 510K clearance and dual CLIA waiver might be the appropriate application mechanism. RP performed testing of 300 samples comparing the red and black beads using sera from clinical sites in Lyon and ICT and western blots for the Cincinnatti epidemiologic study. Three western blots for Chicago samples were performed at LDBIO. The scientists at LDBio did not interfere with the performing of the tests, the recording, interpretation of the results nor the reported conclusions of any work at any academic site. All these studies were performed independently in the academic centers. There was no payment to the scientists. At Hôpital Bichat, Paris and in Morocco studies were/are being reported separately. LDBIO did provide resources to support operating expenses and reagents, but not in the USA or Colombia. RP and DL participated in editing initial and final drafts of the manuscript. All other authors have declared no conflict of interest.
